# Synthesis of Enantiomerically Pure N-Boc-Protected 1,2,3-Triaminopropylphosphonates and 1,2-Diamino-3-Hydroxypropylphosphonates

**DOI:** 10.3390/molecules24213857

**Published:** 2019-10-25

**Authors:** Aleksandra Trocha, Dorota G. Piotrowska, Iwona E. Głowacka

**Affiliations:** Bioorganic Chemistry Laboratory, Faculty of Pharmacy, Medical University of Lodz, Muszynskiego 1, 90-151 Lodz, Poland; aleksandra.trocha@umed.lodz.pl (A.T.); dorota.piotrowska@umed.lodz.pl (D.G.P.)

**Keywords:** aziridinephosphonates, aziridine ring opening, aminopropylphosphonates

## Abstract

All possible isomers of 1,2,3-tri(*N*-*tert*-butoxycarbonylamino)propylphosphonate **6** were synthesized from the respective diethyl [*N*-(1-phenylethyl)]-1-benzylamino-2,3-epiiminopropylphosphonates **5** via opening the aziridine ring with trimethylsilyl azide (TMSN_3_) followed by hydrogenolysis in the presence of di-*tert*-butyl dicarbonate (Boc_2_O). [*N*-(1-phenylethyl)]-1-benzylamino-2,3-epiiminopropylphosphonates (1*R*,2*R*,1′*S*)-**5a** and (1*S*,2*S*,1′*R*)-**5c** were smoothly transformed into diethyl 3-acetoxy-1-benzylamino-2-[*N*-(1-phenylethyl)amino]propylphosphonates (1*R*,2*R*,1′*S*)-**9a** and (1*S*,2*S*,1′*R*)-**9c**, respectively by the opening of the aziridine ring with acetic acid. Transformations of [*N*-(1-phenylethyl)]-1-benzylamino-2,3-epiiminopropylphosphonates (1*S*,2*R*,1′*S*)-**5b** and (1*R*,2*S*,1′*R*)-**5d** into diethyl 3-acetoxy-1-benzylamino-2-[(1-phenylethyl)amino]propylphosphonates (1*S*,2*R*,1′*S*)-**9b** and (1*R*,2*S*,1′*R*)-**9d** were accompanied by the formation of ethyl {1-(*N*-benzylacetamido)-3-hydroxy-2-[(1-phenylethyl)amino]propyl}phosphonate (1*S*,2*R*,1′*S*)-**10b** and (1*R*,2*S*,1′*R*)-**10d** and 3-(*N*-benzylacetamido)-4-[*N*-(1-phenylethyl)]amino-1,2-oxaphospholane (3*S*,4*R*,1′*S*)-**11b** and (3*R*,4*S*,1′*R*)-**11d** as side products. Diethyl (1*R*,2*R*)-, (1*S*,2*S*)-, (1*S*,2*R*)- and (1*R*,2*S*)-3-acetoxy-1,2-di(*N*-*tert*-butoxycarbonylamino)propylphosphonates **7a**–**7d** were obtained from the respective 3-acetoxy-1-benzylamino-2-[*N*-(1-phenylethyl)amino]propylphosphonates **9a**–**9d** by hydrogenolysis in the presence of Boc_2_O.

## 1. Introduction

**Figure 1:**
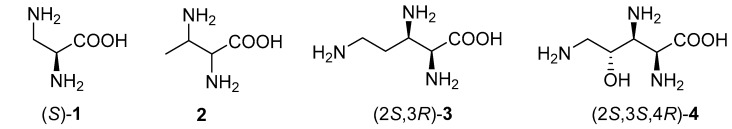
Examples of naturally occurring polyamino acids.

Aside from their fundamental role as components of proteins, amino acids are also of great interest in organic synthesis since they may serve as chiral building blocks for the preparation of new pharmacologically valuable compounds, and they may also be used in peptide synthesis. Non-proteinogenic amino acids are an important class of compounds since they are structural fragments of complex natural products and possess interesting biological properties. Among them, the amino acids functionalized with an additional amino group appear especially interesting (Figure 1). Important diamino acids include (*S*)-2,3-diaminopropanoic acid (*S*)-**1**, which is found in the structure of capreomycin [1,2] and dipeptides Sch3717 and A19009 exhibiting antifungal properties [1,3]. The 3-(*N*-methyl) derivative of (*S*)-**1** [β-*N*-methylamino-L-alanine] is a strong neurotoxin found in the majority of cyanobacterial genera [4,5,6,7,8,9,10], while its 3-(*N*-oxalyl) analogue has been isolated from the grass pea, *Lathyrus sativus* [1,11,12]. On the other hand, alanosine [(*S*)-2-amino-3-[hydroxy(nitroso)amino] propanoic acid] produced by the fermentation of *Streptomyces alanosinicus*, has been reported to possess antineoplastic properties [13,14,15,16]. Isomeric 2,3-diaminobutanoic acids **2** have been recognized as structural fragments of several polypeptide antibiotics [4,5,6,7,8,9,10]. Similarly, the 3-(*N*-methyl) derivative of 2,3-diaminobutanoic acid is an important subunit in uridyl peptide antibiotics [4,5,6,7,8,9,10]. 2,3-Diaminopropanoic and 2,5-diaminopentanoic acids are both part of the structure of cyclotheonamide B, a cyclopeptide isolated from the marine sponge *Theonella swinhoei*, which acts as an inhibitor of serine proteases [13,17,18,19]. (2*S*,3*R*)-2,3,5-Triaminopentanoic acid **3** and (2*S*,3*S*,4*R*)-2,3,5-triamino-4-hydroxypentanoic acid **4** are triamino acids found in the structure of streptothricin and capreomycin type antibiotics [20,21].

**Scheme 1:**
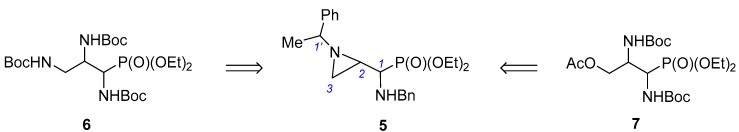
Retrosynthesis of diethyl 1,2,3-triaminopropylphosphonates and 3-acetoxy-1,2-diaminopropylphosphonates.

The interest in the synthesis of phosphonates as bioisosteres of amino acids has been growing over recent decades, as many examples of biologically active compounds have been identified [22,23]. Recently, we succeeded in the synthesis of four enantiomers of diethyl 1,2-diaminopropylphosphonates by the application of the Kabachnik‒Fields reaction of enantiomerically pure and configurationally stable *N*-(1-phenylethyl)aziridine-2-carbaldehydes followed by catalytic hydrogenolysis of the resulting isomeric 1-benzylamino-2,3-epiiminopropylphosphonates **5** [24]. Prompted by previous studies on reactivity of the aziridinephosphonates [24,25,26,27] we concluded that compound **5** would serve as a convenient substrate for the synthesis of the N-Boc-protected 1,2,3-triaminopropylphosphonates **6** and 1,2-diamino-3-acetoxypropylphosphonates **7** when the respective nitrogen and oxygen nucleophiles are applied for the opening of the aziridine ring in phosphonate **5** (Scheme 1).

## 2. Results and Discussion

Enantiomerically pure 1-benzylamino-2,3-epiiminopropylphosphonates (1*R*,2*R*,1′*S*)-**5a** and (1*S*,2*R*,1′*S*)-**5b** as well as (1*S*,2*S*,1′*R*)-**5c** and (1*R*,2*S*,1′*R*)-**5d** were prepared as described previously [24] by the application of enantiomerically pure *N*-(1-phenylethyl)aziridine-2-carbaldehydes in the one-pot three-component Kabachnik‒Fields reaction.

**Scheme 2:**
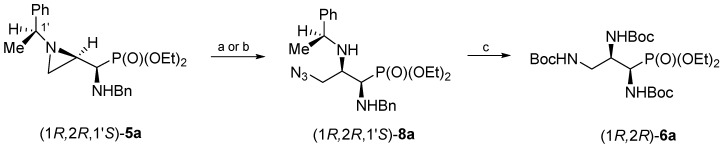
Reactions and conditions: a. trimethylsilyl azide, CH_2_Cl_2_, rt, 15 d, 81%; b. TMSN_3_, rt, 3 d, 76%; c. H_2_, Pd/C, di-*tert*-butyl dicarbonate, EtOH, 3 d, 86%.

The treatment of a dichloromethane solution of aziridinephosphonate (1*R*,2*R*,1′*S*)-**5a** with trimethylsilyl azide at room temperature led to the formation of 3-azidophosphonate (1*R*,2*R*,1′*S*)-**8a** after 15 days (Scheme 2). Phosphonate (1*R*,2*R*,1′*S*)-**8a** was obtained in 81% yield after chromatographic purification. However, the same aziridine ring opening of (1*R*,2*R*,1′*S*)-**5a** with trimethylsilyl azide was accomplished in 3 days and in good yield (76%) when the reaction was carried out without solvent. Regioselectivity of the aziridine ring opening at C3 was evident from ^1^H and ^13^C NMR spectral data of 3-azidophosphonates **8** with chemical shifts of *H*–C3 and the appearance of a two-bond H–C3–H coupling. Careful analysis of crude reaction mixtures showed that a minor regioisomer (opening at C2) was not formed. Then subsequent catalytic reduction of the azide function in (1*R*,2*R*,1′*S*)-**8a** together with the simultaneous hydrogenolytic removal of (*S*)-1-phenylethyl and benzyl groups in the presence of di-*tert*-butyl dicarbonate (Boc_2_O) gave (1*R*,2*R*)-tri(*N*-Boc-amino)propylphosphonate (1*R*,2*R*)-**6a** in 86% yield.

**Scheme 3:**
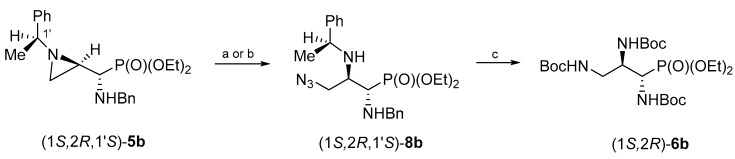
Reactions and conditions: a. TMSN_3_, CH_2_Cl_2_, rt, 3 d, 71%; b. TMSN_3_, rt, 3 d, 72%; c. H_2_, Pd/C, Boc_2_O, EtOH, 3 d, 67%.

The synthesis of (1*S*,2*R*)-tri(*N*-Boc-amino)propylphosphonate (1*S*,2*R*)-**6b** was accomplished in an analogous manner starting from the isomeric aziridinephosphonate (1*S*,2*R*,1′*S*)-**5b** (Scheme 3). In this case, a complete conversion of (1*S*,2*R*,1′*S*)-**5b** into (1*S*,2*R*)-**8b** was achieved in 3 days regardless of whether the reaction was performed with trimethylsilyl azide in dichloromethane or without a solvent. Hydrogenolysis of (1*S*,2*R*)-**8b** proceeded smoothly leading to the formation of (1*S*,2*R*)-**6b** in 67% yield.

**Scheme 4:**
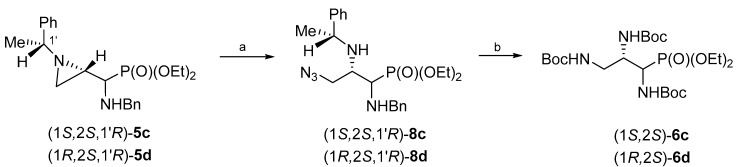
Reactions and conditions: a. TMSN_3_, rt, 3 d, 74% for **8c** and 77% for **8d**; b. H_2_, Pd/C, Boc_2_O, EtOH, 3 d, 91% for **6c** and 70% for **6d**.

We reasoned that the syntheses of the other two enantiomers of tri(*N*-Boc-amino)propylphosphonates, namely (1*S*,2*S*)-**6c** and (1*R*,2*S*)-**6d**, could be accomplished using aziridinephosphonates **5** having the chiral auxiliary with the opposite configuration, i.e., (*R*)-1-phenylethyl moiety (Scheme 4). Thus, when aziridinephosphonates (1*S*,2*S*,1′*R*)-**5c** and (1*R*,2*S*,1′*R*)-**5d** were treated with neat trimethylsilyl azide azidophosphonates (1*S*,2*S*,1′*R*)-**8c** and (1*R*,2*S*,1′*R*)-**8d** were formed in 74% and 77% yield, respectively. The catalytic hydrogenolysis of (1*S*,2*S*,1′*R*)-**8c** and (1*R*,2*S*,1′*R*)-**8**d in the presence of Boc_2_O produced tri(*N*-Boc-amino)phosphonates (1*S*,2*S*)-**6c** and (1*R*,2*S*)-**6d** in 91% and 70% yield, respectively.

We noticed that isomeric aziridinephosphonates **5** could also serve as convenient substrates for synthesis of the protected 1,2-diamino-3-hydroxypropylphosphonates **7** when an appropriate oxygen nucleophile is applied to open the aziridine ring (Scheme 1).

**Scheme 5:**
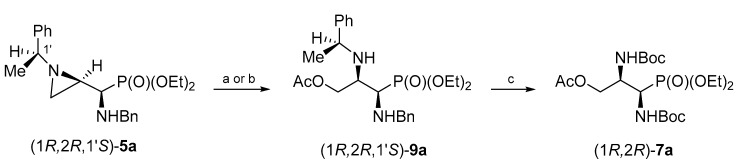
Reactions and conditions: a. glacial AcOH, rt, 24 h, 59%; b. glacial AcOH, microwave, 35‒40 °C, 320 W, 1.5 h, 61%; c. H_2_, Pd(OH)_2_/C, Boc_2_O, EtOH, 18 h, 15 bar, 74%.

When phosphonate (1*R*,2*R*,1′*S*)-**5a** was treated with glacial acetic acid at room temperature for 24 h opening of the aziridine ring at C3 was observed. Phosphonate (1*R*,2*R*,1′*S*)-**9a** was isolated in 59% yield after the chromatographic purification. However, irradiation of the reaction mixture in a microwave oven allowed us to shorten the reaction time to 1.5 h. Thus, phosphonate (1*R*,2*R*,1′*S*)-**9a** was obtained in 61% yield after purification on a silica gel column. Again, regioselectivity of the aziridine ring opening at C3 was evident from ^1^H and ^13^C NMR spectral data of 3-acetoxyphosphonates **9,** with chemical shifts of *H*–C3 and the appearance of a two-bond H–C3–H coupling. Careful analysis of crude reaction mixtures showed that a minor regioisomer (opening at C2) was not formed. Hydrogenolytic removal of *N*-(1-phenylethyl) and *N*-benzyl groups in (1*R*,2*R*,1′*S*)-**9a** performed in the presence of Boc_2_O gave *O*-acetyl-*N,N*-diBoc-protected phosphonate (1*R*,2*R*)-**7a** in 74% yield after purification by column chromatography (Scheme 5).

**Scheme 6:**
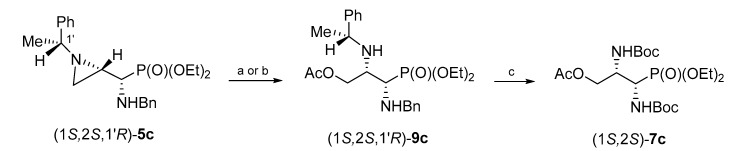
Reactions and conditions: a. glacial AcOH, rt, 24 h, 53%; b. glacial AcOH, microwave oven, 35‒40 °C, 320 W, 1.5 h, 54%; c. H_2_, Pd(OH)_2_/C, Boc_2_O, EtOH, 18 h, 15 bar, 75%.

Similarly, phosphonate (1*S*,2*S*,1′*R*)-**9c** was synthesized from aziridinephosphonate (1*S*,2*S*,1′*R*)-**5c** and glacial acid as a nucleophile. Pure 3-acetoxyphosphonate (1*S*,2*S*,1′*R*)-**9c** was obtained in 53% yield when the reaction was conducted at room temperature for 24 h, or in 54% when microwave irradiation was applied for 1.5 h. The hydrogenolysis of (1*S*,2*S*,1′*R*)-**9c** in the presence of Boc_2_O gave *O*-acetyl-*N,N*-diBoc-protected phosphonate (1*S*,2*S*)-**7c** in 75% yield after column chromatography (Scheme 6).

By analogy, in order to obtain enantiomerically pure phosphonates (1*S*,2*R*,1′*S*)-**9b** and (1*R*,2*S*,1′*R*)-**9d**, the aziridinephosphonates (1*S*,2*R*,1′*S*)-**5b** and (1*R*,2*S*,1′*R*)-**5d** were used as starting materials. Surprisingly, when phosphonates (1*S*,2*R*,1′*S*)-**5b** or (1*R*,2*S*,1′*R*)-**5d** were treated with acetic acid under similar conditions as described above for stereoisomers (1*R*,2*R*,1′*S*)-**5a** or (1*S*,2*S*,1′*R*)-**5c**, the formation of complex mixtures of products was observed.

In the attempts to optimize reaction conditions for the reaction of phosphonate (1*S*,2*R*,1′*S*)-**5b** with acetic acid, several parameters were changed including the reaction time, temperature, solvent/neat and the application of microwave irradiation.

**Scheme 7:**
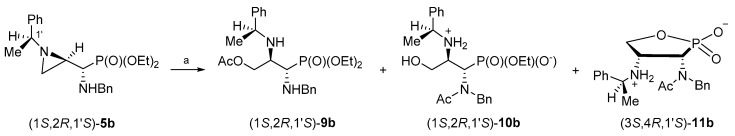
Reactions and conditions: a. AcOH, CH_2_Cl_2_, reflux, 9 h, 24% for **9b**, 22% for **10b**, 4% for **11b**.

When a dichloromethane solution of aziridinephosphonate (1*S*,2*R*,1′*S*)-**5b** was heated with glacial acetic acid for 9 h, a mixture of phosphonate (1*S*,2*R*,1′*S*)-**9b** (41%), the starting phosphonate (1*S*,2*R*,1′*S*)-**5b** (16%), monophosphonate ester (1*S*,2*R*,1′*S*)-**10b** (22%), 1,2-oxaphospholane (3*S*,4*R*,1′*S*)-**11b** (5%), and other unidentified by-products (ca. 16%) was formed (Scheme 7). After the purification of the crude reaction mixture on a silica gel column, phosphonate (1*S*,2*R*,1′*S*)-**9b** was obtained in 24% yield. Additional purification of selected fractions on a HPLC column allowed us to isolate a monophosphonate ester (1*S*,2*R*,1′*S*)-**10b** and 1,2-oxaphospholane (3*S*,4*R*,1′*S*)-**11b** in 10% and 4% yields, respectively.

**Scheme 8:**
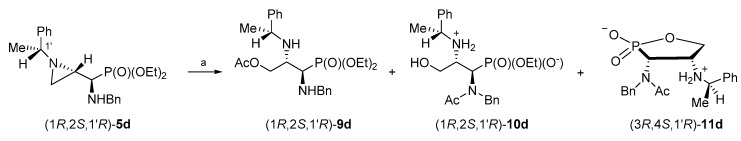
Reactions and conditions: a. AcOH, CH_2_Cl_2_, reflux, 9 h, 30% for **9d**, 7% for **10d**, 12% for **11d**.

Similarly, treatment of aziridinephosphonate (1*R*,2*S*,1′*R*)-**5d** with glacial acetic acid in dichloromethane at reflux for 9 h resulted in the formation of a mixture of phosphonate (1*R*,2*S*,1′*R*)-**9d** (54%), the starting phosphonate (1*R*,2*S*,1′*R*)-**5d** (14%), monophosphonate ester (1*R*,2*S*,1′*R*)-**10d** (10%), 1,2-oxaphospholane (3*R*,4*S*,1′*R*)-**11d** (5%), and with other unidentified by-products (ca. 17%) (Scheme 8). The purification of the crude reaction mixture on a silica gel column allowed us to obtain (1*R*,2*S*,1′*R*)-**9d** in 30% yield. The additional purification of selected fractions by HPLC gave a monophosphonate ester (1*R*,2*S*,1′*R*)-**10d** and 1,2-oxaphospholane (3*R*,4*S*,1′*R*)-**11d** in 7% and 13% yields, respectively.

**Scheme 9:**
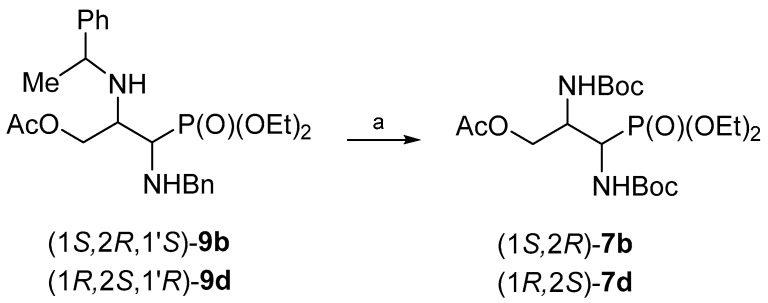
Reactions and conditions: a. H_2_, Pd(OH)_2_/C, Boc_2_O, EtOH, 18 h, 15 bar, 40% for **7b**, 25% for **7d**.

Finally, the hydrogenolysis of (1*S*,2*R*,1′*S*)-**9b** and (1*R*,2*S*,1′*R*)-**9d** in the presence of Boc_2_O gave *O*-acetyl-*N,N*-diBoc-protected phosphonates (1*S*,2*R*)-**7b** and (1*R*,2*S*)-**7d**, in 40% and 25% yield respectively (Scheme 9).

**Scheme 10:**
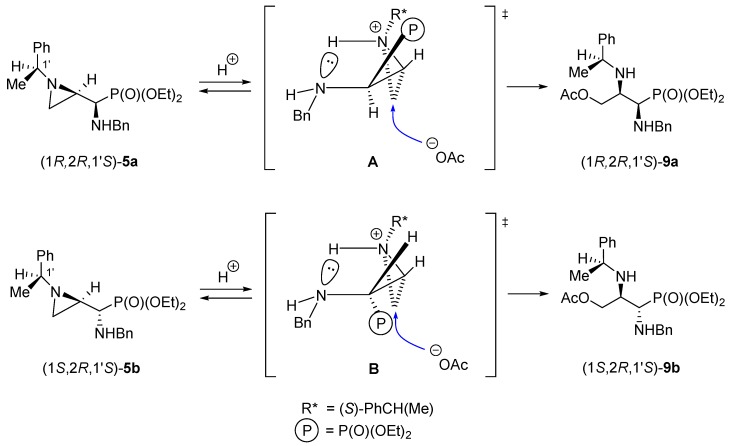
Plausible mechanism of the aziridine ring opening with acetic acid in diastereoisomeric phosphonates (1*R*,2*R*,1′*S*)-**5a** and (1*S*,2*R*,1′*S*)-**5b**.

We observed a significant difference in the aziridine ring openings with acetic acid in diastereoisomeric 1-aminophosphonates (1*R*,2*R*,1′*S*)-**5a** and its enantiomer (1*S*,2*S*,1′*R*)-**5c** when compared with phosphonates (1*S*,2*R*,1′*S*)-**5b** and its enantiomer (1*R*,2*S*,1′*R*)-**5d**. The former pair was cleanly transformed into 3-acetoxypropylphosphonates (1*R*,2*R*,1′*S*)-**9a** and (1*S*,2*S*,1′*R*)-**9c** while the latter pair provided 3-acetoxypropylphosphonates (1*S*,2*R*,1′*S*)-**9b** and its enantiomer (1*R*,2*S*,1′*R*)-**9d** contaminated with the starting materials and other organophosphorus compounds. Undoubtedly, in the presence of acetic acid, protonation of the ring nitrogen atom occurred and formed aziridinium ions **A** and **B** (Scheme 10). If we assume that these ions are additionally stabilized by hydrogen bonding with the BnNH–C1 nitrogen atoms, a spatial disposition of the diethoxyphosphoryl group would have been responsible for the observed differences in reactivity of diastereoisomeric pairs. Thus, the approach of the acetate anion to the less substituted carbon atom (C3) from the opposite side of the protonated nitrogen atom in **A** is sterically facilitated while in **B** the sterically bulky diethoxyphosphoryl group retards the attack of the nucleophile. Similar observations were made several years ago on diastereoisomeric 2,3-epiimino-1-hydroxypropylphosphonates [27].

**Scheme 11:**
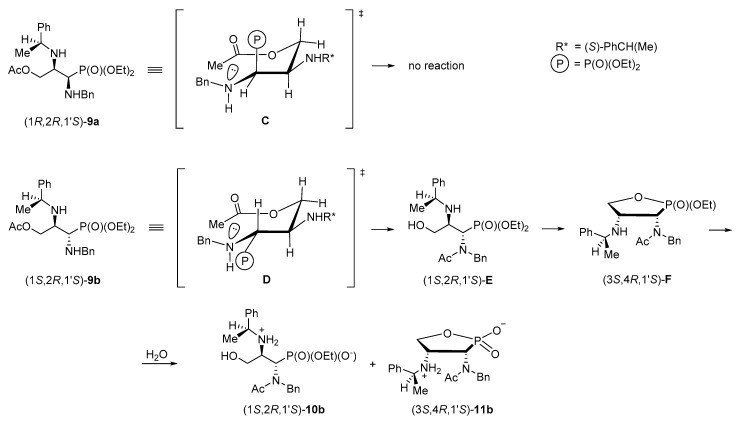
Plausible mechanism of O→N acetyl transfer in (1*S*,2*R*,1′*S*)-**9b** and formation of (1*S*,2*R*,1′*S*)-**10b** and (3*S*,4*R*,1′*S*)-**11b**.

To rationalize the problem of the oxygen to nitrogen transfer of the acetyl group observed during the acetolysis of phosphonate (1*S*,2*R*,1′*S*)-**5b,** possible transition states were discussed. First, 3-acetoxypropylphosphonate (1*R*,2*R*,1′*S*)-**9a** should be transformed into a chair-like (six membered) conformer **C** which is energetically unfavoured since the bulky diethoxyphosphoryl group is positioned axially and thus the O→N acetyl transfer was not noticed under the reaction conditions applied (Scheme 11). On the other hand, in an analogous chair-like conformer of 3-acetoxypropylphosphonate (1*S*,2*R*,1′*S*)-**9b** (**D**) the diethoxyphosphoryl group is located in the equatorial position minimizing steric interactions. Under these circumstances the acetyl transfer provides (1*S*,2*R*,1′*S*)-**E** as a primary product (not isolated). The 3-hydroxypropylphosphonate readily underwent cyclization to form substituted 1,2-oxaphospholane (3*S*,4*R*,1′*S*)-**F** (also not isolated). However, their prior formation was proved by isolation of monophosphonate ester (1*S*,2*R*,1′*S*)-**10b** [by the ring opening of (3*S*,4*R*,1′*S*)-**F**] and (3*S*,4*R*,1′*S*)-**11b**.

## 3. Experimental Section

### 3.1. General

^1^H NMR spectra were taken in chloroform-*d* (CDCl_3_), benzene-*d6* (C_6_D_6_) or deuterium oxide (D_2_O) on a Bruker Avance III (600 MHz). For spectra recorded in CDCl_3_ and C_6_D_6_ tetramethylsilane (TMS) was used as an internal standard; chemical shifts δ are given in ppm with respect to TMS and coupling constants *J* in Hz. Chemical shifts in D_2_O are referenced to the residual solvent peak, δ = 4.80 ppm. ^13^C NMR and ^31^P NMR spectra were recorded in a ^1^H-decoupled mode for CDCl_3_, C_6_D_6,_ and D_2_O solutions on the Bruker Avance III (600 MHz) spectrometer at 151 and 243 MHz, respectively. IR spectral data were measured on a Bruker Alpha-T FT-IR spectrometer. Melting points were determined on a Boetius apparatus and are uncorrected. Elemental analyses were performed by the Microanalytical Laboratory of the Faculty of Pharmacy (Medical University of Lodz) on a Perkin Elmer PE 2400 CHNS analyzer and their results were found to be in good agreement (±0.3%) with the calculated values. Polarimetric measurements were conducted on an Optical Activity PolAAr 3001 apparatus. Microwave irradiation experiments were carried out in a Plazmatronika RM 800 microwave reactor. The reaction was carried out in a 50 mL glass vial.

The following adsorbents were used: column chromatography, Merck silica gel 60 (70–230 mesh), analytical thin layer chromatography (TLC), Merck TLC plastic sheets silica gel 60 F_254_. TLC plates were developed in dichloromethane‒ethyl acetate and dichlorometane‒methanol solvent systems. Visualization of spots was effected with iodine vapors. All solvents were purified by methods described in the literature.

No special protection measures were taken when working with liquid (oily) aziridinephosphonates even under solvent free conditions.

### 3.2. Ring Opening of Aziridinephosphonates 5 with trimethylsilyl azide

#### 3.2.1. Reaction of Aziridinephosphonates 5 with Neat Trimethylsilyl Azide (General Procedure, Method A)

A mixture of aziridinephosphonate **5** (1.00 mmol) and TMSN_3_ (3.00 mmol) was stirred at room temperature for 3 days. The crude product was chromatographed on a silica gel column (12 g of silica gel) with dichloromethane‒ethyl acetate (4:1, *v*/*v*, 500 mL) to give the protected 1,2-diamino-3-azidopropylphosphonates **8**.

#### 3.2.2. Reaction of Aziridinephosphonates 5 with Trimethylsilyl Azide with CH_2_Cl_2_ (General Procedure, Method B)

To a solution of aziridinephosphonate **5** (1.00 mmol) in dichloromethane (4 mL), TMSN_3_ (3.00 mmol) was added at room temperature. The reaction mixture was stirred at ambient temperature for 15 days [for (1*R*,2*R*,1′*S*)-**5a**] or for 3 days [for (1*S*,2*R*,1′*S*)-**5b**] and then was chromatographed on a silica gel column (12 g of silica gel) with dichloromethane‒ethyl acetate (4:1, *v*/*v*, 500 mL) to give the protected 1,2-diamino-3-azidopropylphosphonates **8**.

*Diethyl (1R,2R)-3-azido-1-benzylamino-2-*[*(S)-1-phenylethylamino*]*propylphosphonate* [(1*R*,2*R*,1′*S*)-**8a**]: According to method A from aziridinephosphonate (1*R*,2*R*,1′*S*)-**5a** (0.213 g, 0.529 mmol), phosphonate (1*R*,2*R*,1′*S*)-**8a** (0.179 g, 76%) was obtained as a yellowish oil.

According to method B from aziridinephosphonate (1*R*,2*R*,1′*S*)-**5a** (0.736 g, 1.829 mmol), phosphonate (1*R*,2*R*,1′*S*)-**8a** (0.664 g, 81%) was obtained as a yellowish oil. *R_f_* = 0.47 (dichloromethane‒ethyl acetate, 1:2, *v*/*v*). IR (film): ν = 3449, 3314, 3028, 2978, 2927, 2101, 1453, 1234, 1025, 762, 701 cm^−1^.${\left[\alpha \right]}_{D}^{20}$ = −73.1 (*c* 1.46, CHCl_3_). ^1^H NMR (600 MHz, C_6_D_6_): δ = 7.29−7.27 (m, 4H), 7.19−7.15 (m, 4H), 7.11−7.08 (m, 1H), 7.07−7.04 (m, 1H), 3.99−3.92 (m, 2H, CH_3_C*H*_2_OP), 3.89 (d, ^2^*J*_(HCH)_ = 13.0 Hz, 1H, *H*_a_CH_b_Ph), 3.85−3.77 (m, 2H, CH_3_C*H*_2_OP), 3.76 (d, ^2^*J*_(HCH)_ = 13.0 Hz, 1H, H_a_C*H*_b_Ph), 3.72 (q, ^3^*J*_(HCCH)_ = 6.6 Hz, 1H, *H*CCH_3_), 3.36 (ddd, ^2^*J*_(HCH)_ = 12.3 Hz, ^3^*J*_(HCCH)_ = 4.6 Hz, ^4^*J*_(HCCCP)_ = 1.7 Hz, 1H, *H*_a_CH_b_CCP), 3.29 (dd, ^2^*J*_(HCH)_ = 12.3 Hz, ^3^*J*_(HCCH)_ = 6.4 Hz, 1H, H_a_C*H*_b_CCP), 3.07 (dd, ^2^*J*_(HCP)_ = 14.5 Hz, ^3^*J*_(HCCH)_ = 4.4 Hz, 1H, *H*CP), 3.02 (dddd, ^3^*J*_(HCCP)_ = 13.6 Hz, ^3^*J*_(HCCH)_ = 6.4 Hz, ^3^*J*_(HCCH)_ = 4.6 Hz, ^3^*J*_(HCCH)_ = 4.4 Hz, 1H, *H*CCP), 1.27 (d, ^3^*J*_(HCCH)_ = 6.6 Hz, 3H, HCC*H*_3_), 1.00 (t, ^3^*J*_(HCCH)_ = 7.1 Hz, 3H, C*H*_3_CH_2_OP), 0.95 (t, ^3^*J*_(HCCH)_ = 7.1 Hz, 3H, C*H*_3_CH_2_OP). ^13^C NMR (151 MHz, CDCl_3_): δ= 144.8, 139.8, 128.5, 128.4, 128.3, 127.2, 127.1, 126.8, 62.4 (d, ^2^*J*_(COP)_ = 7.2 Hz, *C*OP), 61.9 (d, ^2^*J*_(COP)_ = 7.2 Hz, *C*OP), 55.2, 55.1 (d, ^1^*J*_(CP)_ = 149.3 Hz, *C*P), 54.3 (d, ^3^*J*_(CNCP)_ = 5.6 Hz, *C*H_2_Ph), 53.4 (d, ^2^*J*_(CCP)_ = 3.8 Hz, *C*CP), 51.1 (d, ^3^*J*_(CCCP)_ = 8.7 Hz, *C*CCP), 24.8 (C*C*H_3_), 16.6 (d, ^3^*J*_(CCOP)_ = 5.8 Hz, *C*COP), 16.4 (d, ^3^*J*_(CCOP)_ = 5.8 Hz, *C*COP). ^31^P NMR (243 MHz, C_6_D_6_): δ = 26.51. Anal. Calcd. for C_22_H_32_N_2_O_3_P: C, 59.31; H, 7.24; N, 15.72. Found: C, 59.25; H, 7.23; N, 15.62.

*Diethyl (1S,2S)-3-azido-1-benzylamino-2-*[*(R)-1-phenylethylamino*]*propylphosphonate* [(1*S*,2*S*,1′*R*)-**8c**]: According to method A from aziridinephosphonate (1*S*,2*S*,1′*R*)-**5c** (0.201 g, 0.499 mmol), phosphonate (1*S*,2S,1′*R*)-**8c** (0.165 g, 74%) was obtained as a yellowish oil. ${\left[\alpha \right]}_{D}^{20}$ = +70.7 (*c* 1.23, CHCl_3_). Anal. Calcd. for C_22_H_32_N_2_O_3_P: C, 59.31; H, 7.24; N, 15.72. Found: C, 59.03; H, 7.45; N, 15.62. NMR spectra of (1*S*,2S,1′*R*)-**8c** identical with its enantiomer (1*R*,2*R*,1′*S*)-**8a**.

*Diethyl (1S,2R)-3-azido-1-benzylamino-2-*[*(S)-1-phenylethylamino*]*propylphosphonate* [(1*S*,2*R*,1′*S*)-**8b**]: According to method A from aziridinephosphonate (1*S*,2*R*,1′*S*)-**5b** (0.207 g, 0.514 mmol), phosphonate (1*S*,2*R*,1′*S*)-**8b** (0.164 g, 72%) was obtained as a yellowish oil.

According to method B from aziridinephosphonate (1*S*,2*R*,1′*S*)-**5b** (0.100 g, 0.248 mmol), phosphonate (1*S*,2*R*,1′*S*)-**8b** (0.078 g, 71%) was obtained as a yellowish oil. *R_f_* = 0.58 (dichloromethane‒ethyl acetate, 1:2). IR (film): ν = 3448, 3317, 3028, 2980, 2099, 1453, 1234, 1026, 756, 701 cm^−1^. ${\left[\alpha \right]}_{D}^{20}$ = −26.3 (*c* 1.20, CHCl_3_). ^1^H NMR (600 MHz, C_6_D_6_): δ = 7.32−7.02 (m, 10H), 4.00−3.96 (m, 2H, CH_3_C*H*_2_OP), 3.90−3.85 (m, 2H, CH_3_C*H*_2_OP), 3.69 (q, ^3^*J*_(HCCH)_ = 6.5 Hz, 1H, *H*CCH_3_), 3.64 (d, ^2^*J*_(HCH)_ = 13.0 Hz, 1H, *H*_a_CH_b_Ph), 3.55 (d, ^2^*J*_(HCH)_ = 13.0 Hz, 1H, H_a_C*H*_b_Ph), 3.52 (dd, ^2^*J*_(HCH)_ = 12.5 Hz, ^3^*J*_(HCCH)_ = 5.3 Hz, 1H, *H*_a_CH_b_CCP), 3.35 (dd, ^2^*J*_(HCH)_ = 12.5 Hz, ^3^*J*_(HCCH)_ = 5.6 Hz, 1H, H_a_C*H*_b_CCP), 3.03 (dd, ^2^*J*_(HCP)_ = 19.9 Hz, ^3^*J*_(HCCH)_ = 4.7 Hz, 1H, *H*CP), 2.99 (dddd, ^3^*J*_(HCCP)_ = 20.6 Hz, ^3^*J*_(HCCH)_ = 5.6 Hz, ^3^*J*_(HCCH)_ = 5.3 Hz, ^3^*J*_(HCCH)_ = 4.7 Hz, 1H, *H*CCP), 1.26 (d, ^3^*J*_(HCCH)_ = 6.5 Hz, 3H, HCC*H*_3_), 1.03 and 1.02 (2 × t, ^3^*J*_(HCCH)_ = 7.0 Hz, 6H, 2 × C*H*_3_CH_2_OP). ^13^C NMR (151 MHz, CDCl_3_): δ = 145.5, 139.7, 128.5, 128.4, 128.3, 127.1, 126.9, 62.4 (d, ^2^*J*_(COP)_ = 7.1 Hz, *C*OP), 61.9 (d, ^2^*J*_(COP)_ = 7.1 Hz, *C*OP), 55.8, 55.5 (d, ^1^*J*_(CP)_ = 156.9 Hz, *C*P), 55.1 (d, ^3^*J*_(CNCP)_ = 5.6 Hz, CH_2_Ph), 52.6 (d, ^3^*J*_(CCCP)_ = 6.4 Hz, *C*CCP), 51.7 (d, ^2^*J*_(CCP)_ = 2.1 Hz, *C*CP), 24.4, 16.6 (d, ^3^*J*_(CCOP)_ = 5.9 Hz, *C*COP), 16.5 (d, ^3^*J*_(CCOP)_ = 5.9 Hz, *C*COP). ^31^P NMR (243 MHz, CDCl_3_): δ= 26.19. Anal. Calcd. for C_22_H_32_N_2_O_3_P: C, 59.31; H, 7.24; N, 15.72. Found: C, 59.05; H, 7.41; N, 15.79.

*Diethyl (1R,2S)-3-azido-1-benzylamino-2-[(R)-1-phenylethylamino]propylphosphonate* [(1*R*,2*S*,1′*R*)-**8d**]: According to method A from aziridinephosphonate (1*R*,2*S*,1′*R*)-**5d** (0.221 g, 0.549 mmol), phosphonate (1*R*,2*S*,1′*R*)-**8d** (0.189 g, 77%) was obtained as a yellowish oil. ${\left[\alpha \right]}_{D}^{20}$ = +25.6 (*c* 1.33, CHCl_3_). Anal. Calcd. for C_22_H_32_N_2_O_3_P: C, 59.31; H, 7.24; N, 15.72. Found: C, 59.19; H, 7.32; N, 15.57. NMR spectra of (1*R*,2*S*,1′*R*)-**8d** identical with its enantiomer (1*S*,2*R*,1′*S*)-**8b**.

### 3.3. Hydrogenolysis of Diethyl 3-Azido-1-Benzylamino-2-[1-Phenylethylamino]propylphosphonates 8 (General Procedure)

A solution of diethyl 3-azido-1-benzylamino-2-(1-phenylethylamino)propylphosphonate **8** (1.00 mmol) in ethanol (5 mL) containing Boc_2_O (3.3 mmol) was stirred under an atmospheric pressure of hydrogen over 20% Pd/C (50 mg) at room temperature for 3 days. The suspension was filtered through a layer of Celite, then the solution was concentrated and chromatographed on a silica gel column (20 g of silica gel) with dichloromethane‒methanol (100:1, *v*/*v*, 400 mL and then 50:1, *v*/*v*, 100 mL). The appropriate fractions were collected and crystallized from hexane to produce corresponding enantiomerically pure diethyl 1,2,3-tri(*N*-*tert*-butoxycarbonylamino)propylphosphonate **6**.

*Diethyl (1R,2R)-1,2,3-tri(N-tert-butoxycarbonylamino)propylphosphonate* [(1*R*,2*R*)-**6a**]: From phosphonate (1*R*,2*R*,1′*S*)-**8a** (0.106 g, 0.238 mmol), (1*R*,2*R*)-**6a** (0.108 g, 86%) was obtained as a white solid. *R_f_* = 0.46 (dichloromethane‒methanol, 10:1, v/v). Mp = 131‒134 °C. IR (KBr): ν = 3355, 2980, 2931, 1710, 1680, 1520, 1172. ${\left[\alpha \right]}_{D}^{20}$ = +19.6 (*c* 1.14, CHCl_3_). ^1^H NMR (600 MHz, CDCl_3_): δ = 5.44 (brs, 1H, *H*NCHP), 5.33 (brs, 1H, *H*NCHCHP), 5.25 (brs, 1H, *H*NCH_2_), 4.20‒4.16 (m, 4H, 2 × CH_3_C*H*_2_OP), 4.13−4.08 (m, 1H, C*H*CP), 4.06−4.01 (m, 1H, *H*CP), 3.51−3.32 (m, 2H, C*H*_2_CCP), 1.46 (s, 27H, 3 × (C*H*_3_)_3_C), 1.36 and 1.35 (2 × t, ^3^*J*_(HCCH)_ = 7.0 Hz, 6H, 2 × C*H*_3_CH_2_OP). ^13^C NMR (151 MHz, CDCl_3_): δ = 156.8 (C=O), 156.3 (C=O), 155.8 (d, ^3^*J*_(CNCP)_ = 6.0 Hz, C=O), 80.2, 79.6, 79.5, 63.1 (d, ^2^*J*_(COP)_ = 6.6 Hz, *C*OP), 62.7 (d, ^2^*J*_(COP)_ = 6.6 Hz, *C*OP), 51.7, 49.5 (d, ^1^*J*_(CP)_ = 156.4 Hz, *C*P), 42.3, 28.4, 28.3, 28.2, 16.4 (d, ^3^*J*_(CCOP)_ = 5.6 Hz, *C*COP), 16.3 (d, ^3^*J*_(CCOP)_ = 6.1 Hz, *C*COP). ^31^P NMR (243 MHz, CDCl_3_): δ= 22.54. Anal. Calcd. for C_22_H_44_N_3_O_9_P × 0.25 H_2_O: C, 49.85; H, 8.46; N, 7.93. Found: C, 49.72; H, 8.42; N, 7.65.

*Diethyl (1S,2S)-1,2,3-tri(N-tert-butoxycarbonylamino)propylphosphonate* [(1*S*,2*S*)-**6c**]: From phosphonate (1*S*,2*S*,1′*R*)-**8c** (0.096 g, 0.215 mmol), (1*S*,2*S*)-**6c** (0.103 g, 91%) was obtained as a white solid. Mp = 136‒138 °C. ${\left[\alpha \right]}_{D}^{20}$ = ‒21.5 (*c* 1.17, CHCl_3_). Anal. Calcd. for C_22_H_44_N_3_O_9_P × 0.5 H_2_O: C, 49.43; H, 8.49; N, 7.86. Found: C, 49.70; H, 8.42; N, 7.62. NMR spectra of (1*S*,2S)-**6c** identical with its enantiomer (1*R*,2*R*)-**6a**.

*Diethyl (1S,2R)-1,2,3-tri(N-tert-butoxycarbonylamino)propylphosphonate* [(1*S*,2*R*)-**6b**]: From phosphonate (1*S*,2*R*,1′*S*)-**8b** (0.075 g, 0.168 mmol), (1*S*,2*R*)-**6b** (0.066 g, 67%) was obtained as a white solid. *R_f_* = 0.63 (dichloromethane‒methanol, 10:1, *v*/*v*). Mp = 126–128 °C. IR (KBr): ν = 3380, 3352, 2978, 1697, 1537, 1172. ${\left[\alpha \right]}_{D}^{20}$ = +16.2 (*c* 1.31, CHCl_3_). ^1^H NMR (600 MHz, CDCl_3_): δ = 5.58 (brs, 1H, *H*NCHP), 5.14 (brs, 1H, *H*NCHCHP), 5.13 (brs, 1H, *H*NCH_2_), 4.42‒4.28 (m, 1H, *H*CP), 4.24−4.12 (m, 4H, 2 × CH_3_C*H*_2_OP), 4.10‒3.98 (m, 1H, *H*CCP), 3.47−3.31(m, 2H, C*H*_2_CCP), 1.47 and 1.46 (2 × s, 27H, 3 × (C*H*_3_)_3_C), 1.37 and 1.35 (2 × t, ^3^*J*_(HCCH)_ = 7.1 Hz, 6H, 2 × C*H*_3_CH_2_OP). ^13^C NMR (151 MHz, CDCl_3_): δ = 156.5 (C=O), 155.9 (C=O), 155.2 (d, ^3^*J*_(CNCP)_ = 6.0 Hz, C=O), 80.4, 79.7, 79.6, 63.4 (d, ^2^*J*_(COP)_ = 6.8 Hz, *C*OP), 62.7 (d, ^2^*J*_(COP)_ = 6.8 Hz, *C*OP), 52.3, 48.2 (d, ^1^*J*_(CP)_ = 152.4 Hz, *C*P), 42.3, 28.4, 28.2, 16.4 (d, ^3^*J*_(CCOP)_ = 5.8 Hz, *C*COP), 16.3 (d, ^3^*J*_(CCOP)_ = 5.8 Hz, *C*COP). ^31^P NMR (243 MHz, CDCl_3_): δ = 22.40. Anal. Calcd. for C_22_H_44_N_3_O_9_P × 0.25 H_2_O: C, 49.85; H, 8.46; N, 7.93. Found: C, 49.70; H, 8.21; N, 7.66.

*Diethyl (1R,2S)-1,2,3-tri(N-tert-butoxycarbonylamino)propylphosphonate* [(1*R*,2*S*)-**6d**]: From phosphonate (1*R*,2*S*,1′*R*)-**8d** (0.099 g, 0.222 mmol), (1*R*,2*S*)**-6d** (0,090 g, 70%) was obtained as a white solid. Mp = 129–133 °C. ${\left[\alpha \right]}_{D}^{20}$ = −16.5 (*c* 1.33, CHCl_3_). Anal. Calcd. for C_22_H_44_N_3_O_9_P × 0.25 H_2_O: C, 49.85; H, 8.46; N, 7.93. Found: C, 49.79; H, 8.54; N, 7.65. NMR spectra of (1*R*,2*S*)-**6d** identical with its enantiomer (1*S*,2*R*)-**6b**.

### 3.4. Ring Opening of Aziridinephosphonates 5 with AcOH

#### 3.4.1. Reaction of Aziridinephosphonates 5 with AcOH at Room Temperature (General Procedure, Method A)

A solution of phosphonate (1*R*,2*R*,1′*S*)-**5a** or (1*S*,2*S*,1′*R*)-**5c** (1.00 mmol) in glacial acetic acid (34.9 mmol) was stirred at room temperature for 24 h. The reaction mixture was then concentrated in vacuo with toluene (3 × 10 mL). The crude product was chromatographed on a silica gel column (10 g of silica gel) with dichloromethane‒ethyl acetate (4:1, v/v, 400 mL) to give the pure protected 1,2-diamino-3-hydroxypropylphosphonate **9**.

#### 3.4.2. Reaction of Aziridinephosphonates 5 with AcOH in the Microwave Reactor (General Procedure, Method B)

A solution of phosphonate (1*R*,2*R*,1′*S*)-**5a** or (1*S*,2*S*,1′*R*)-**5c** (1.00 mmol) in glacial acetic acid (34.9 mmol) was microwave irradiated in the microwave reactor (Plazmatronika RM 800, 320 W) at 35–40 °C for 1.5 h. After cooling, the reaction mixture was concentrated in vacuo with toluene (3 × 10 mL). The crude product was chromatographed on a silica gel column column (10 g of silica gel) with dichloromethane‒ethyl acetate (4:1, *v*/*v*, 400 mL) to give the protected 1,2-diamino-3-hydroxypropylphosphonate **9**.

*Diethyl (1R,2R)-3-acetoxy-1-benzylamino-2-*[*(S)-1-phenylethylamino]propylphosphonate* [(1*R*,2*R*,1′*S*)-**9a**]: According to the method A from aziridinephosphonate (1*R*,2*R*,1′*S*)-**5a** (0.158 g, 0.393 mmol), phosphonate (1*R*,2*R*,1′*S*)-**9a** (0.107 g, 59%) was obtained as a yellowish oil.

According to the method B from aziridinephosphonate (1*R*,2*R*,1′*S*)-**5a** (0.203 g, 0.504 mmol), phosphonate (1*R*,2*R*,1′*S*)-**9a** (0.143 g, 61%) was obtained as a yellowish oil. *R_f_* = 0.42 (dichloromethane‒ethyl acetate, 1:2, *v*/*v*). IR (film): ν = 3459, 3335, 3028, 2979, 2929, 2867, 1739, 1453, 1367, 1235, 1027, 963, 763, 702 cm^−1^. ${\left[\alpha \right]}_{D}^{20}$ = −78.0 (*c* 1.28, CHCl_3_). ^1^H NMR (600 MHz, CDCl_3_): δ = 7.34−7.22 (m, 10H), 4.22 (ddd, ^2^*J*_(HCH)_ = 11.1 Hz, ^3^*J*_(HCCH)_ = 4.1 Hz, ^4^*J*_(HCCCP)_ = 2.9 Hz, 1H, *H*_a_CH_b_CCP), 4.19−4.12 (m, 3H, CH_3_C*H_2_*OP, H_a_C*H*_b_CCP), 4.08‒3.97 (m, 3H, CH_3_C*H_2_*OP, *H*_a_CH_b_Ph), 3.90 (q, ^3^*J*_(HCCH)_ = 6.6 Hz, 1H, *H*CCH_3_), 3.82 (d, ^2^*J*_(HCH)_ = 12.8 Hz, 1H, H_a_C*H_b_*Ph), 3.02 (dd, ^2^*J*_(HCP)_ *=* 14.8 Hz, ^3^*J*_(HCCH)_ = 3.7 Hz, *H*CP), 2.98 (dddd, ^3^*J*_(HCCP)_ = 14.8 Hz, ^3^*J*_(HCCH)_ = 7.2 Hz, ^3^*J*_(HCCH)_ = 4.1 Hz, *J* = 3.7 Hz, *H*CCP), 1.94 (s, 3H, C*H*_3_CO), 1.33 (d, ^3^*J*_(HCCH)_ = 6.6 Hz, HCC*H*_3_), 1.31 and 1.25 (2 × t, ^3^*J*_(HCCH)_ = 7.1 Hz, 6H, 2 × C*H_3_*CH_2_OP). ^1^H NMR (600 MHz, C_6_D_6_): δ = 7.37−7.36 (m, 2H), 7.31−7.30 (m, 2H), 7.19−7.13 (m, 4H), 7.07−7.04 (m, 2H), 4.40 (ddd, ^2^*J*_(HCH)_ = 11.0 Hz, ^3^*J*_(HCCH)_ = 4.5 Hz, ^4^*J*_(HCCCP)_ = 2.7 Hz, 1H, *H*_a_CH_b_CCP), 4.33 (dd, ^2^*J*_(HCH)_ = 11.0 Hz, ^3^*J*_(HCCH)_ = 7.4 Hz, H_a_C*H*_b_CCP), 4.04‒4.00 (m, 2H, CH_3_C*H*_2_OP), 3.91 (d, ^2^*J*_(HCH)_ = 12.8 Hz, *H*_a_CH_b_Ph), 3.84 (d, ^2^*J*_(HCH)_ = 12.8 Hz, H_a_C*H*_b_Ph), 3.89‒3.82 (m, 3H, C*H*_2_OP, *H*CCH_3_), 3.24 (dddd, ^3^*J*_(HCCP)_ = 12.0 Hz, ^3^*J*_(HCCH)_ = 7.4 Hz, ^3^*J*_(HCCH)_ = 4.5 Hz, ^3^*J*_(HCCH)_ = 3.2 Hz, 1H, *H*CCP), 3.16 (dd, ^2^*J*_(HCP)_ = 15.0 Hz, ^3^*J*_(HCCH)_ = 3.2 Hz, 1H, *H*CP), 1.53 (s, 3H, C*H*_3_CO), 1.25 (d, ^3^*J*_(HCCH)_ = 6.6 Hz, 3H, HCC*H*_3_), 1.03 (t, ^3^*J*_(HCCH)_ = 7.1 Hz, 3H, C*H*_3_CH_2_OP), 0.96 (t, ^3^*J*_(HCCH)_ = 7.1 Hz, 3H, C*H*_3_CH_2_OP). ^13^C NMR (151 MHz, CDCl_3_): δ= 170.6 (*C*=O), 144.9, 139.9, 128.6, 128.4, 128.3, 127.1, 127.0, 126.9, 63.0 (d, ^3^*J*_(CCCP)_ = 10.3 Hz, *C*CCP), 62.2 (d, ^2^*J*_(COP)_ = 7.2 Hz, *C*OP), 61.8 (d, ^2^*J*_(COP)_ = 7.2 Hz, *C*OP), 55.1, 54.7 (d, ^1^*J*_(CP)_ = 150.1 Hz, *C*P), 53.3 (d, ^2^*J*_(CCP)_ = 3.7 Hz, *C*CP), 53.1 (d, ^3^*J*_(CNCP)_ = 4.7 Hz, *C*H_2_Ph), 25.1, 20.9, 16.5 (d, ^3^*J*_(CCOP)_ = 5.7 Hz, *C*COP), 16.4 (d, ^3^*J*_(CCOP)_ = 5.7 Hz, *C*COP). ^31^P NMR (243 MHz, CDCl_3_): δ = 27.02. Anal. Calcd. for C_24_H_35_N_2_O_5_P: C, 62.32; H, 7.63; N, 6.06. Found: C, 62.12; H, 7.64; N, 6.12.

*Diethyl (1S,2S)-3-acetoxy-1-benzylamino-2-*[*(R)-1-phenylethylamino]propylphosphonate* [(1*S*,2*S*,1′*R*)-**9c**]: According to the method A from aziridinephosphonate (1*S*,2*S*,1′*R*)-**5c** (0.224 g, 0.556 mmol), phosphonate (1*S*,2S,1′*R*)-**9c** (0.137 g, 53%) was obtained as a yellowish oil.

According to the method B from aziridinephosphonate (1*S*,2*S*,1′*R*)-**5c** (0.201 g, 0.499 mmol), phosphonate (1*S*,2S,1′*R*)-**9c** (0.125 g, 54%) was obtained as a yellowish oil. ${\left[\alpha \right]}_{D}^{20}$ = +75.2 (*c* 1.34, CHCl_3_). Anal. Calcd. for C_24_H_35_N_2_O_5_P: C, 62.32; H, 7.63; N, 6.06. Found: C, 62.12; H, 7.50; N, 6.21. NMR spectra of (1*S*,2S,1′*R*)-**9c** identical with its enantiomer (1*R*,2*R*,1′*S*)-**9a**.

### 3.5. Ring Opening of (1S,2R,1′S)- and (1R,2S,1′R)-Aziridinephosphonates 5 with AcOH (General Procedure)

A solution of aziridinephosphonate (1*S*,2*R*,1′*S*)-**5b** or (1*R*,2*S*,1′*R*)-**5d** (1.00 mmol) in dichloromethane (4 mL) containing acetic acid (3.00 mmol) was refluxed for 9 h. The reaction mixture was then concentrated in vacuo with toluene (3 × 10 mL). The crude product was chromatographed on a silica gel column (15 g of silica gel) with dichloromethane‒ethyl acetate (4:1, *v*/*v*, 650 mL) to give the respective phosphonate **9**. The appropriate fractions were collected and purified by HPLC using a X Bridge Prep, C18, 5 µm, OBD (Optimum Bed Density), 19 × 100 mm column and acetonitrile‒water mixture (28:72, *v*/*v*) as eluent to afford **10** and **11**.

*Diethyl (1S,2R)-3-acetoxy-1-benzylamino-2-*[*(S)-1-phenylethylamino]propylphosphonate* [(1*S*,2*R*,1′*S*)-**9b**]: From aziridinephosphonate (1*S*,2*R*,1′*S*)-**5b** (0.157 g, 0.390 mmol), phosphonate (1*S*,2*R*,1′*S*)-**9b** (0.044 g, 24%) was obtained as a yellowish oil. *R_f_* = 0.40 (dichloromethane‒ethyl acetate, 1:2, *v*/*v*). IR (film): ν = 3456, 3321, 3028, 2978, 1738, 1453, 1367, 1235, 1028, 963, 702 cm^−1^. ${\left[\alpha \right]}_{D}^{20}$ = −35.3 (*c* 0.98, CHCl_3_). ^1^H NMR (600 MHz, CDCl_3_): δ = 7.35−7.23 (m, 8H), 7.20−7.18 (m, 2H), 4.36 (dd, ^2^*J*_(HCH)_ = 11.3 Hz, ^3^*J*_(HCCH)_ = 5.2 Hz, 1H, *H_a_*CH_b_CCP), 4.29 (dd, ^2^*J*_(HCH)_ = 11.3 Hz, ^3^*J*_(HCCH)_ = 6.5 Hz, 1H, H_a_C*H_b_*CCP), 4.24−4.19 (m, 2H, CH_3_C*H_2_*OP), 4.13−4.08 (m, 2H, CH_3_C*H_2_*OP), 3.95 (q, ^3^*J*_(HCCH)_ = 6.6 Hz, 1H, *H*CCH_3_), 3.56 (AB, *J*_AB_ = 13.1 Hz, 1H, *H*_a_CH_b_Ph), 3.53 (AB, *J*_AB_ = 13.1 Hz, 1H, H_a_C*H_b_*Ph), 2.98 (dddd, ^3^*J*_(HCCP)_ = 23.5 Hz, ^3^*J*_(HCCH)_ = 6.5 Hz, ^3^*J*_(HCCH)_ = 5.2 Hz, ^3^*J*_(HCCH)_ = 4.3 Hz, 1H, *H*CCP), 2.95 (dd, ^2^*J*_(HCP)_ = 14.1 Hz, ^3^*J*_(HCCH)_ = 4.3 Hz, 1H, *H*CP), 2.02 (s, 3H, C*H*_3_CO), 1.36 (d, ^3^*J*_(HCCH)_ = 6.6 Hz, 3H, HCC*H*_3_), 1.34 and 1.32 (2 × t, ^3^*J*_(HCCH)_ = 7.1 Hz, 6H, 2 × C*H_3_*CH_2_OP). ^13^C NMR (151 MHz, CDCl_3_): δ = 170.8 (*C*=O), 145.6, 139.7, 128.5, 128.3, 128.2, 127.1, 127.05, 127.0, 64.3 (*C*CCP), 62.7 (d, ^2^*J*_(COP)_ = 7.3 Hz, *C*OP), 61.7 (d, ^2^*J*_(COP)_ = 7.3 Hz, *C*OP), 55.7, 55.6 (d, ^1^*J*_(CP)_ = 150.1 Hz, *C*P), 53.5 (d, ^2^*J*_(CCP)_ = 4.0 Hz, *C*CP), 52.2 (d, ^3^*J*_(CNCP)_ = 9.7 Hz, *C*H_2_Ph), 24.9, 20.9, 16.6 (d, ^3^*J*_(CCOP)_ = 5.8 Hz, *C*COP), 16.4 (d, ^3^*J*_(CCOP)_ = 5.8 Hz, *C*COP). ^31^P NMR (243 MHz, CDCl_3_): δ= 25.74. Anal. Calcd. for C_24_H_35_N_2_O_5_P: C, 62.32; H, 7.63; N, 6.06. Found: C, 62.09; H, 7.89; N, 5.99.

*Diethyl (1R,2S)-3-acetoxy-1-benzylamino-2-*[*(R)-1phenylethylamino]propylphosphonate* [(1*R*,2*S*,1′*R*)-**9d**]: From aziridinephosphonate (1*R*,2*S*,1′*R*)-**5d** (0.147 g, 0.365 mmol), phosphonate (1*R*,2*S*,1′*R*)-**9d** (0.051 g, 30%) was obtained as a yellowish oil. ${\left[\alpha \right]}_{D}^{20}$ = +35.9 (*c* 0.70, CHCl_3_). Anal. Calcd. for C_24_H_35_N_2_O_5_P: C, 62.32; H, 7.63; N, 6.06. Found: C, 62.28; H, 7.93; N, 6.03. NMR spectra of (1*R*,2*S*,1′*R*)-**9d** identical with its enantiomer (1*S*,2*R*,1′*S*)-**9b**.

*Ethyl (1S,2R)-*{*1-(N-benzylacetamido)-3-hydroxy-2-*[*(S)-(1-phenylethyl)amino*]*propyl*}*phosphonate* [(1*S*,2*R*,1’*S*)-**10b**]: From aziridinephosphonate (1*S*,2*R*,1′*S*)-**5b** (0.157 g, 0.390 mmol), phosphonate (1*S*,2*R*,1′*S*)-**10b** (0.017 g, 10%) was obtained as a white solid. Retention time: *t*_R_ = 4.03 min. Mp = 104–106 °C. IR (film): ν = 3386, 3063, 2982, 1644, 1591, 1453, 1359, 1217, 1043, 948, 702. ${\left[\alpha \right]}_{D}^{20}$ = −56.6 (*c* 1.09, CHCl_3_). ^1^H NMR (600 MHz, CDCl_3_): δ **=** 7.48−7.37 (m, 7H), 7.34−7.32 (m, 1H), 7.18 (d, *J* = 7.2 Hz, 2H), 6.58 (brs, 1H, N*H*), 5.36 (d, *J* = 16.9 Hz, 1H, *H*_a_CH_b_Ph), 5.05 (q, *J* = 7.1 Hz, 1H, *H*CCH_3_), 4.86 (d, *J* = 16.9 Hz, 1H, H_a_C*H_b_*Ph), 4.54−4.46 (m, 3H, C*H_2_*CCP, *H*CP), 4.05−3.99 (m, 1H, *H*CHOP), 3.78−3.73 (m, 1H, HC*H*OP), 3.72−3.65 (m, 1H, *H*CCP), 2.06 (s, 3H, C*H*_3_CO), 1.92 (d, ^3^*J*_(HCCH)_ = 7.2 Hz, 3H, HCC*H*_3_), 1.11 (t, ^3^*J*_(HCCH)_ = 7.1 Hz, 3H, C*H_3_*CH_2_OP). ^13^C NMR (151 MHz, CDCl_3_): δ = 166.9 (d, ^3^*J*_(CNCP)_ = 5.7 Hz, *C*=O), 138.6, 134.1, 129.5, 129.2, 128.7, 128.2, 126.4, 125.9, 67.5, 60.7 (d, ^2^*J*_(COP)_ = 6.2 Hz, *C*OP), 60.2 (d, ^3^*J*_(CNCP)_ = 7.7 Hz), 60.0 (d, ^1^*J*_(CP)_ = 139.0 Hz, *C*P), 55.8, 49.1, 18.6, 16.8 (d, ^3^*J*_(CCOP)_ = 5.7 Hz, *C*COP), 12.8. ^31^P NMR (243 MHz, CDCl_3_): δ= 6.93. Anal. Calcd. for C_22_H_31_N_2_O_5_P × 0.5 H_2_O: C, 59.59; H, 7.27; N, 6.32. Found: C, 59.47; H, 7.20; N, 6.15.

*Ethyl (1R,2S)-{1-(N-benzylacetamido)-3-hydroxy-2-*[*(R)-(1-phenylethyl)amino]propyl}phosphonate* [(1*R*,2*S*,1’*R*)-**10d**]: From aziridinephosphonate (1*R*,2*S*,1′*R*)-**5d** (0.147 g, 0.365 mmol), phosphonate (1*R*,2*S*,1′*R*)-**10d** (0.012 g, 7%) was obtained as a white solid. Mp = 101‒103 °C. ${\left[\alpha \right]}_{D}^{20}$ = +55.6 (*c* 0.88, CHCl_3_). Anal. Calcd. for C_22_H_31_N_2_O_5_P × 1.25 H_2_O: C, 57.83; H, 7.39; N, 6.13. Found: C, 57.58; H, 7.02; N, 5.85. NMR spectra of (1*R*,2*S*,1′*R*)-**10d** identical with its enantiomer (1*S*,2*R*,1′*S*)-**10b**.

*(3S,4R)-3-(N-benzylacetamido)-4-*[*(S)-N-(1-phenylethyl)*]*amino-1,2-oxaphospholane* [(3*S*,4*R*,1′*S*)-**11b**]: From aziridinephosphonate (1*S*,2*R*,1′*S*)-**5b** (0.157 g, 0.390 mmol), 1,2-oxaphospholane (3*S*,4*R*,1′*S*)-**11b** (0.006 g, 4%) was obtained as a thick colorless oil. Retention time: *t*_R_ = 1.91 min. IR (film): ν = 3406, 3063, 2980, 1650, 1594, 1238, 1087, 703. ${\left[\alpha \right]}_{D}^{20}$ = ‒24.7 (*c* 0.75, MeOH). ^1^H NMR (600 MHz, D_2_O): δ = 7.49‒7.37 (m, 10H), 5.39 (q, ^3^*J*_(HCCH)_ = 7.0 Hz, 1H, *H*CCH_3_), 5.14 (dddd, ^3^*J*_(HCCP)_ = 21.0 Hz, ^3^*J*_(HCCH)_ = 11.3 Hz, ^3^*J*_(HCCH)_ = 4.6 Hz, ^3^*J*_(HCCH)_ = 2.3 Hz, 1H, *H*CCP), 5.06 (d, ^2^*J*_(HCH)_ = 15.4 Hz, 1H, *H*_a_CH_b_Ph), 4.75 (d, ^2^*J*_(HCH)_ = 15.4 Hz, 1H, H_a_C*H*_b_Ph), 4.05 (d, ^3^*J*_(HCCH)_ = 11.3 Hz, 1H, *H*CP), 3.60 (ddd, ^2^*J*_(HCH)_ = 11.8 Hz, ^3^*J*_(HCOP)_ = 7.0 Hz, ^3^*J*_(HCCH)_ = 4.6 Hz, 1H, *H_a_*CH_b_CCP), 3.55 (ddd, ^3^*J*_(HCOP)_ = 20.6 Hz, ^2^*J*_(HCH)_ = 11.8 Hz, ^3^*J*_(HCCH)_ = 2.3 Hz, 1H, H_a_C*H_b_*CCP), 2.47 (s, 3H, C*H*_3_CO), 1.70 (d, ^3^*J*_(HCCH)_ = 7.0 Hz, 3H, HCC*H*_3_). ^13^C NMR (151 MHz, D_2_O): δ = 166.0 (d, ^3^*J*_(CNCP)_ = 2.1 Hz, C=O), 138.1, 132.6, 129.4, 129.3, 129.1, 128.8, 128.6, 126.6, 64.0 (d, ^3^*J*_(CCCP)_ = 10.4 Hz, *C*CCP), 63.2 (d, ^2^*J*_(CCP)_ = 5.5 Hz, *C*CP), 55.4, 54.6 (d, ^1^*J*_(CP)_ = 130.6 Hz, *C*P), 49.9, 16.6, 11.8. ^31^P NMR (243 MHz, D_2_O): δ = 29.81. Anal. Calcd. for C_20_H_25_N_2_O_4_P × 1.25 H_2_O: C, 58.46; H, 6.75; N, 6.82. Found: C, 58.23; H, 6.51; N, 6.63.

*(3R,4S)-3-(N-benzylacetamido)-4-*[*(R)-N-(1-phenylethyl)]amino-1,2-oxaphospholane* [(3*R*,4*S*,1′*R*)-**11d**]: From aziridinephosphonate (1*R*,2*S*,1′*R*)-**5d** (0.147 g, 0.365 mmol), 1,2-oxaphospholane (3*R*,4*S*,1′*R*)-**11d** (0.019 g, 13%) was obtained as a thick colorless solid. ${\left[\alpha \right]}_{D}^{20}$ = +24.9 (*c* 0.82, MeOH). Anal. Calcd. for C_20_H_25_N_2_O_4_P × 1.75 H_2_O: C, 57.21; H, 6.84; N, 6.67. Found: C, 56.96; H, 6.73; N, 6.71. NMR spectra of (3*R*,4*S*,1′*R*)-**11d** identical with its enantiomer (3*S*,4*R*,1′*S*)-**11b**.

### 3.6. Hydrogenolysis of Diethyl 3-Acetoxy-1-Benzylamino-2-[1-Phenylethylamino]propylphosphonates 9 (General Procedure)

A solution of diethyl 3-acetoxy-1-benzylamino-2-(1-phenylethylamino)propylphosphonate **9** (1.00 mmol) in ethanol (10 mL) containing Boc_2_O (2.2 mmol) was stirred in a pressure reactor (15 bar) over 20% Pd(OH)_2_/C (50 mg) at room temperature for 18 h. The suspension was filtered through a layer of Celite, then the solution was concentrated and chromatographed on a silica gel column (17 g of silica gel) with dichloromethane‒methanol (100:1, *v*/*v*, 500 mL and then 50:1, *v*/*v*, 250 mL) to give respective enantiomerically pure diethyl 3**-**acetoxy-1,2-di(*N*-Boc-amino)propylophosphonates **7**.

*Diethyl (1R,2R)-3-acetoxy-1,2-di(N-Boc-amino)propylophosphonate* [(1*R*,2*R*)-**7a**]: From phosphonate (1*R*,2*R*,1′*S*)-**9a** (0.160 g, 0.346 mmol), phosphonate (1*R*,2*R*)-**7a** (0.120 g, 74%) was obtained as a thick colorless oil. *R_f_* = 0.57 (dichloromethane‒methanol, 10:1, *v*/*v*). IR (film): ν = 3320, 2980, 2933, 1748, 1715, 1684, 1240, 1166, 1050, 1025 cm^−1^. ${\left[\alpha \right]}_{D}^{20}$ = +7.0 (*c* 1.23, CHCl_3_). ^1^H NMR (600 MHz, CDCl_3_): δ = 5.39 (brs, 1H, *H*NCHP), 5.24 (brs, 1H, *H*NCHCHP), 4.33−4.30 (m, 1H), 4.30−4.20 (m, 3H), 4.20−4.15(m, 4H, 2 × CH_3_C*H_2_*OP), 2.09 (s, 3H, C*H*_3_CO), 1.45 (s, 18H, 2 × (C*H*_3_)_3_C), 1.35 and 1.34 (2 × t, ^3^*J*_(HCCH)_ = 7.1 Hz, 6H, 2 × C*H_3_*CH_2_OP). ^13^C NMR (151 MHz, CDCl_3_): δ = 170.6 (C=O), 155.7 (C=O), 155.6 (d, ^3^*J*_(CNCP)_ = 6.5 Hz, C=O), 80.3, 79.9, 63.6 (brs), 62.9 (brs), 49.7, 48.1 (d, ^1^*J*_(CP)_ = 155.8 Hz, *C*P), 28.3, 28.2, 20.8, 16.5 (d, ^3^*J*_(CCOP)_ = 5.6 Hz, *C*COP), 16.3 (d, ^3^*J*_(CCOP)_ = 5.9 Hz, *C*COP). ^31^P NMR (243 MHz, CDCl_3_): δ = 22.00. Anal. Calcd. for C_19_H_37_N_2_O_9_P × 0.5 H_2_O: C, 47.80; H, 8.02; N, 5.87. Found: C, 47.91; H, 7.78; N, 5.85.

*Diethyl (1S,2S)-3-acetoxy-1,2-di(N-Boc-amino)propylophosphonate* [(1*S*,2*S*)-**7c**]: From phosphonate (1*S*,2*S*,1′*R*)-**9c** (0.133 g, 0.288 mmol), propylphosphonate (1*S*,2S)-**7c** (0.101 g, 75%) was obtained as a thick colorless oil. ${\left[\alpha \right]}_{D}^{20}$ = −7.2 (*c* 1.21, CHCl_3_). Anal. Calcd. for C_19_H_37_N_2_O_9_P: C, 48.72; H, 7.96; N, 5.98. Found: C, 48.68; H, 8.20; N, 6.14. NMR spectra of (1*S*,2*S*)-**7c** identical with its enantiomer (1*R*,2*R*)-**7a**.

*Diethyl (1S,2R)-3-acetoxy-1,2-di(N-Boc-amino)propylophosphonate* [(1*S*,2*R*)-**7b**]: From phosphonate (1*S*,2*R*,1′*S*)-**9b** (0.040 g, 0.087 mmol), phosphonate (1*S*,2*R*)-**7b** (0.016 g, 40%) was obtained as a thick colorless oil. *R_f_* = 0.52 (dichloromethane‒methanol, 10:1, *v*/*v*). IR (film): ν = 3319, 2958, 2925, 1743, 1712, 1239, 1165,1045, 1026 cm^−1^. ${\left[\alpha \right]}_{D}^{20}$ = +12.7 (*c* 0.71, CHCl_3_). ^1^H NMR (600 MHz, CDCl_3_): δ = 5.48 (d, ^3^*J*_(HNCH)_ = 8.0 Hz, 1H, *H*N), 5.10 (d, ^3^*J*_(HNCH)_ = 10.3 Hz, 1H, *H*N), 4.38 (ddd, *J* = 15.0 Hz, *J* = 10.1 Hz, *J* = 3.1 Hz, 1H), 4.28‒4.13 (m, 7H, *H_a_*CH_b_, H_a_C*H_b_*, C*H*CP, C*H*P, C*H_2_*OP), 2.10 (s, 3H, C*H*_3_CO), 1.48 (s, 9H, (C*H*_3_)C), 1.47 (s, 9H, (C*H*_3_)_3_C), 1.35 and 1.34 (2 × t, ^3^*J*_(HCCH)_ = 7.0 Hz, 6H, 2 × C*H_3_*CH_2_OP). ^13^C NMR (151 MHz, CDCl_3_): δ = 170.6 (C=O), 155.5 (C=O), 155.0 (d, ^3^*J*_(CNCP)_ = 5.4 Hz, C=O), 80.6, 80.0, 63.6, 63.5 (d, ^2^*J*_(COP)_ = 7.1 Hz, *C*OP), 62.6 (d, ^2^*J*_(COP)_ = 7.4 Hz, *C*OP), 50.9 (d, ^2^*J*_(CCP)_ = 3.7 Hz), 47.3 (d, ^1^*J*_(CP)_ = 155.1 Hz, *C*P), 28.3, 28.2, 20.8, 16.5 (d, ^3^*J*_(CCOP)_ = 5.6 Hz, *C*COP), 16.3 (d, ^3^*J*_(CCOP)_ = 6.1 Hz, *C*COP). ^31^P NMR (243 MHz, CDCl_3_): δ= 22.08. Anal. Calcd. for C_19_H_37_N_2_O_9_P × 0.25 H_2_O: C, 48.25; H, 7.99; N, 5.93. Found: C, 48.14; H, 8.27; N, 5.86.

*Diethyl (1R,2S)-3-acetoxy-1,2-di(N-Boc-amino)propylophosphonate* [(1*R*,2*S*)-**7d**]: From propylphosphonate (1*R*,2*S*,1′*R*)-**9d** (0.040 g, 0.087 mmol), propylphosphonate (1*R*,2*S*)-**7d** (0.010 g, 25%) was obtained as a colorless oil. ${\left[\alpha \right]}_{D}^{20}$ = −9.8 (*c* 0.61, CHCl_3_). Anal. Calcd. for C_19_H_37_N_2_O_9_P × 0.5 H_2_O: C, 47.80; H, 8.02; N, 5.87. Found: C, 47.77; H, 7.85; N, 5.87. NMR spectra of (1*R*,2*S*)-**7d** identical with its enantiomer (1*S*,2*R*)-**7b**.

## 4. Conclusions

An efficient synthetic strategy for four enantiomers of diethyl 1,2,3-tri(*N-tert*-butoxycarbonylamino)propylphosphonates **9** was developed. It relies on the opening of the aziridine ring in aziridinephosphonate (1*R*,2*R*,1′*S*)-**5a**, (1*S*,2*R*,1′*S*)-**5b**, (1*S*,2*S*,1′*R*)-**5c** and (1*R*,2*S*,1′*R*)-**5d** with trimethylsilyl azide followed by the reduction of the azido group together with the hydrogenolytic removal of the 1-phenylethyl and benzyl residues and simultaneous protection of the amino functions with a *tert*-butoxycarbonyl group.

Aziridinephosphonate (1*R*,2*R*,1′*S*)-**5a** and (1*S*,2*S*,1′*R*)-**5c** were smoothly converted into syn 3-acetoxy-1-benzylamino-2-[*N*-(1-phenylethyl)amino]propylphosphonates (1*R*,2*R*,1′*S*)-**9a** and (1*S*,2*S*,1′*R*)-**9c** via aziridine ring opening with acetic acid as a nucleophile. The synthesis of anti phosphonates (1*S*,2*R*,1′*S*)-**9b** and (1*R*,2*S*,1′*R*)-**9d** from (1*S*,2*R*,1′*S*)-**5b** and (1*R*,2*S*,1′*R*)-**5d** was less effective, as the formation of several by-products, including the respective monophosphonate esters **10b** and **10d** and 1,2-oxaphospholanes **11b** and **11d**, was noticed. The hydrogenolysis of 3-acetoxy-1-benzylamino-2-[*N*-(1-phenylethyl)amino]propylphosphonates (1*R*,2*R*,1′*S*)-**9a** and (1*S*,2*S*,1′*R*)-**9c** as well as (1*S*,2*R*,1′*S*)-**9b** and (1*R*,2*S*,1′*R*)-**9d** in the presence of Boc_2_O gave 3-acetoxy-1,2-di(*N*-*tert*-butoxycarbonylamino)propylphosphonates, (1*R*,2*R*)-**7a**, (1*S*,2*S*)-**7c**, (1*S*,2*R*)-**7b** and (1*R*,2*S*)-**7d**, respectively, in good yields.

## Supplementary Materials

Supplementary File 1
