# Supplementary material for: Synthesis of Enantiomerically Pure N-Boc-Protected 1,2,3-Triaminopropylphosphonates and 1,2-Diamino-3-Hydroxypropylphosphonates
Source: Molecules. 2019 Oct 25;24(21):3857. doi: 10.3390/molecules24213857 (PMC6864986; doi:10.3390/molecules24213857)
Supplement: Supplementary File 1 [file molecules-24-03857-s001.pdf]

Spin Works 3:

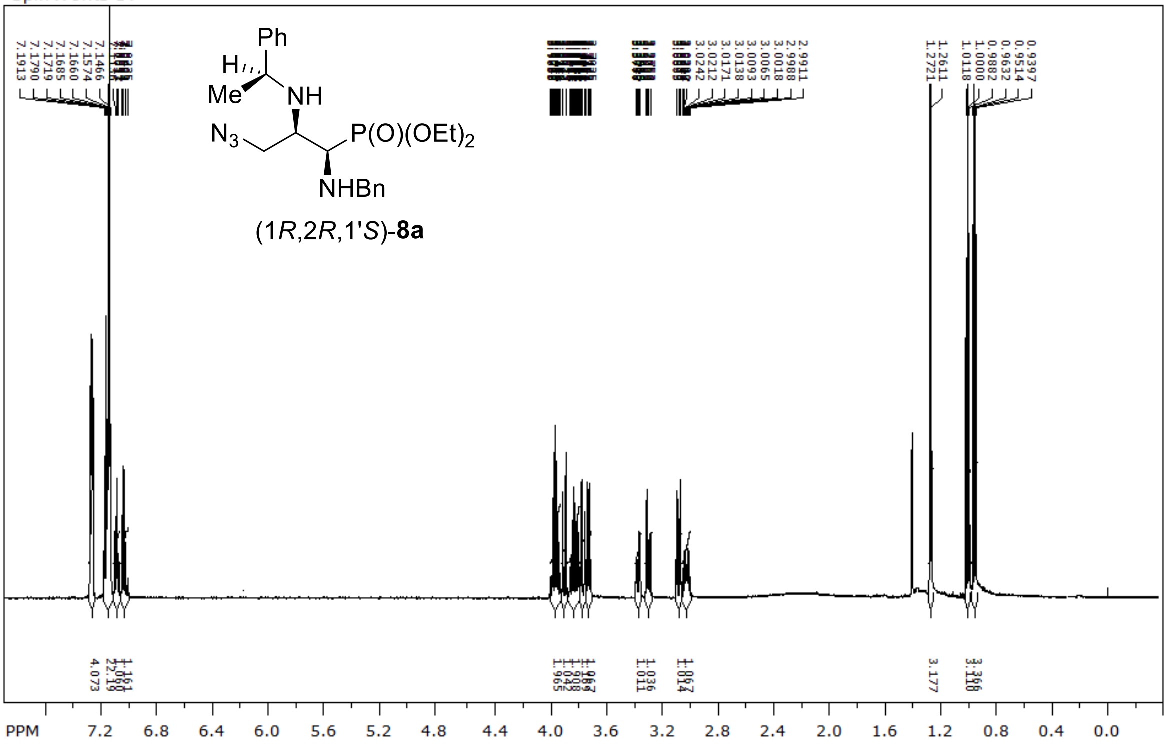

file: ...oniany\(1R,2R,1'S)\abt-0879 1H\fid expt: <zg30>
transmitter freq.: 600.263707 MHz
time domain size: 65536 points
width: 12335.53 Hz = 20.5502 ppm = 0.188225 Hz/pt
number of scans: 16

freq. of 0 ppm: 600.260000 MHz
processed size: 32768 complex points
LB: 0.000 GF: 0.0000
Hz/cm: 198.960 ppm/cm: 0.33145

Spin Works 3: no title

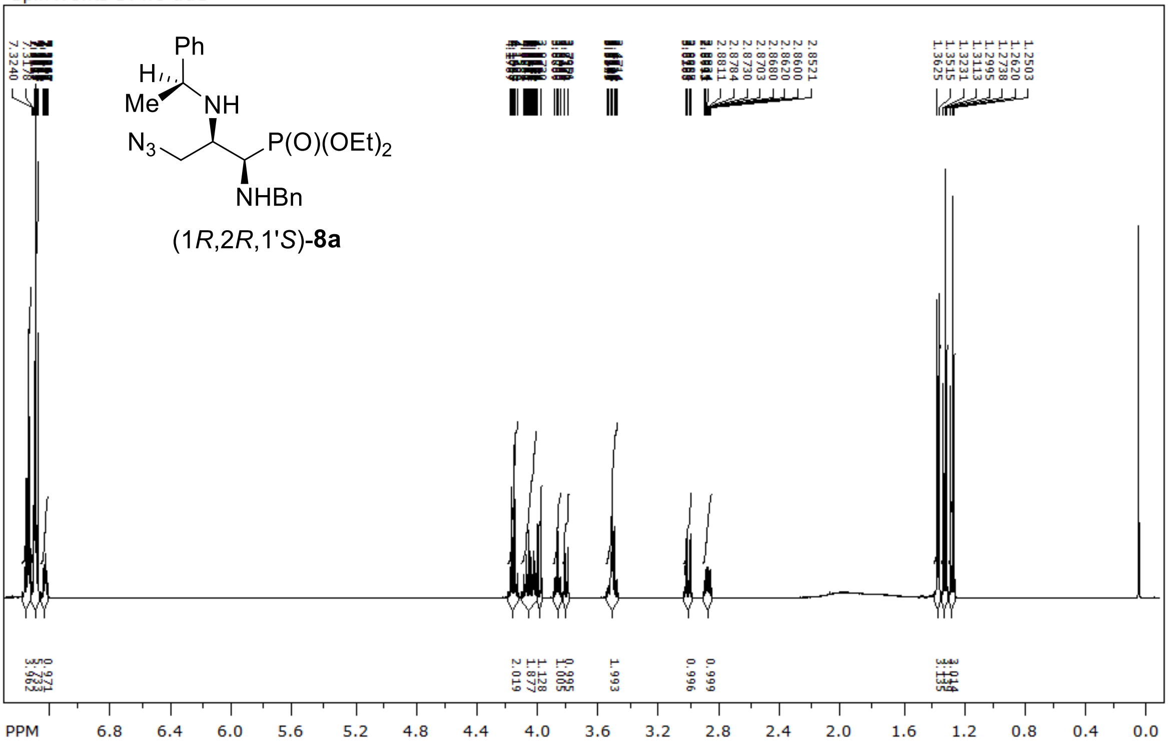

file: ...ktop\NMR\NMR2017UŁ\abt-0774\10\fid expt: <zg30>
transmitter freq.: 600.263707 MHz
time domain size: 65536 points
width: 12335.53 Hz = 20.5502 ppm = 0.188225 Hz/pt
number of scans: 16

freq. of 0 ppm: 600.260000 MHz
processed size: 32768 complex points
LB: 0.000 GF: 0.0000
Hz/cm: 183.043 ppm/cm: 0.30494

SpinWorks 3: no title

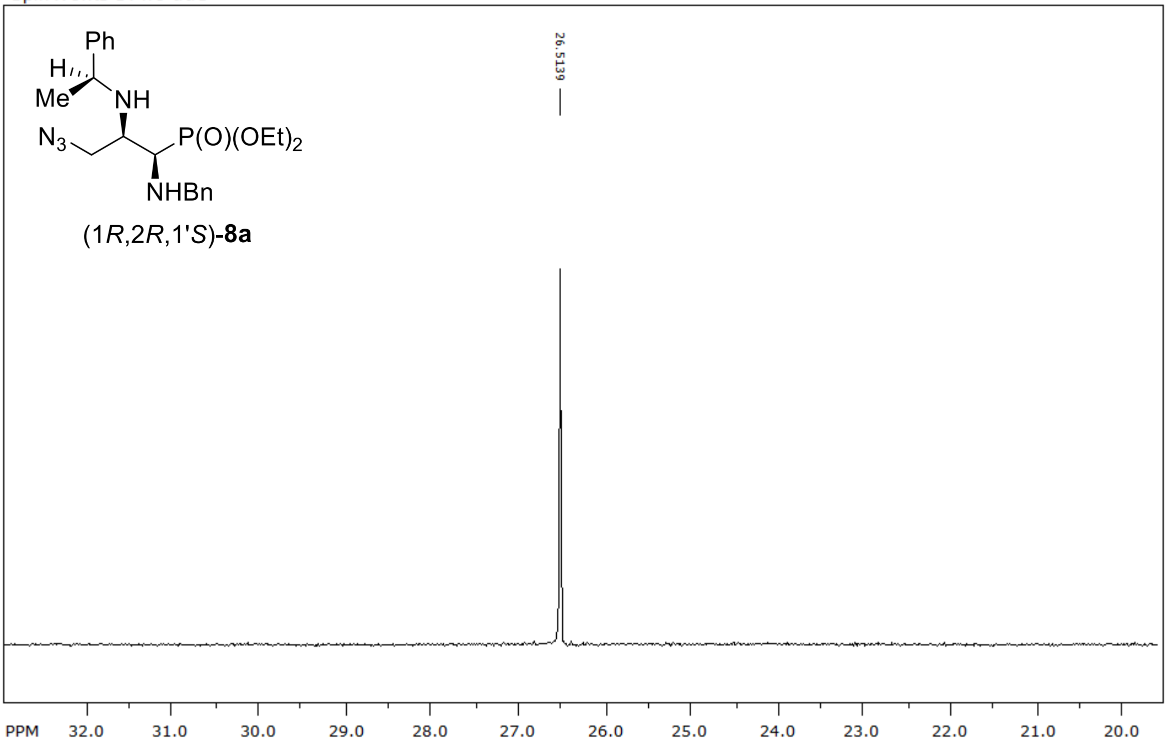

file: ...niany\(1R,2R,1'S)\abt-0874 31P\fid   expt: <zgpg30>
transmitter freq.: 242.977552 MHz
time domain size: 65536 points
width: 96153.85 Hz = 395.7314 ppm = 1.467191 Hz/pt
number of scans: 128

freq. of 0 ppm: 242.989702 MHz
processed size: 32768 complex points
LB: 0.000   GF: 0.0000
Hz/cm: 130.273   ppm/cm: 0.53615

SpinWorks 3: no title

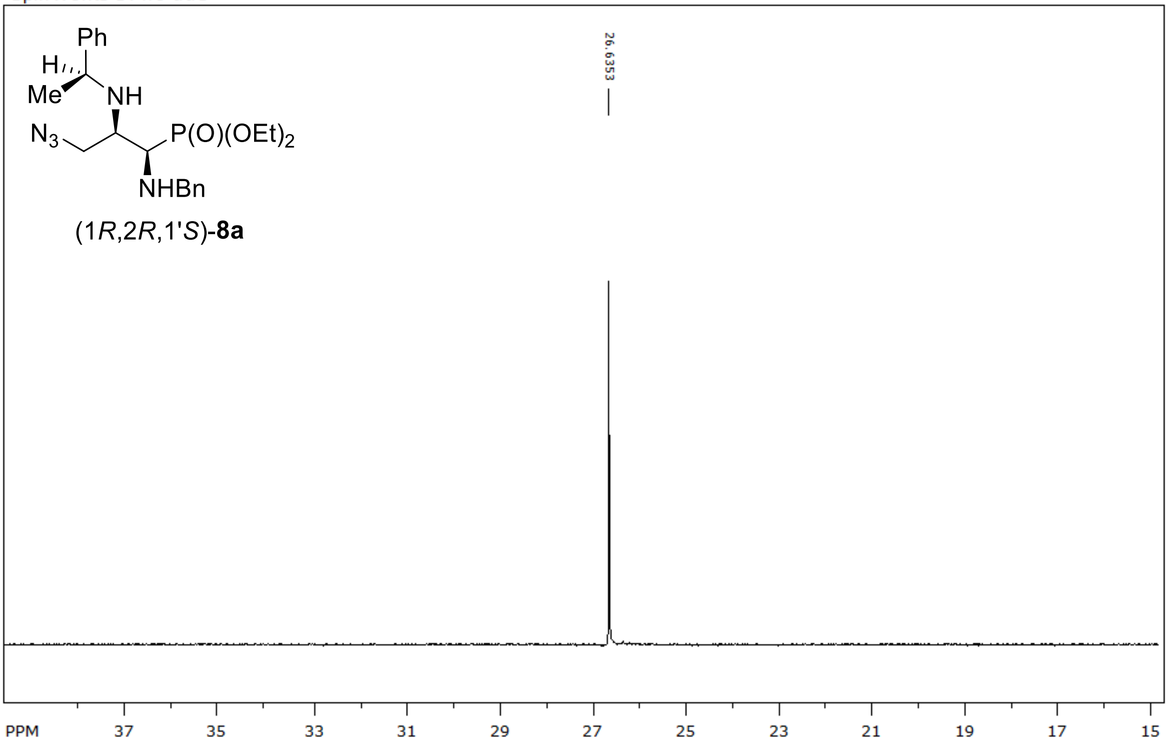

file: ...ktop\NMR\NMR2017Uł\abt-0774\11\fid   expt: <zgpg30>
transmitter freq.: 242.977552 MHz
time domain size: 65536 points
width: 96153.85 Hz = 395.7314 ppm = 1.467191 Hz/pt
number of scans: 128

freq. of 0 ppm: 242.989702 MHz
processed size: 32768 complex points
LB: 0.000   GF: 0.0000
Hz/cm: 241.935   ppm/cm: 0.99571

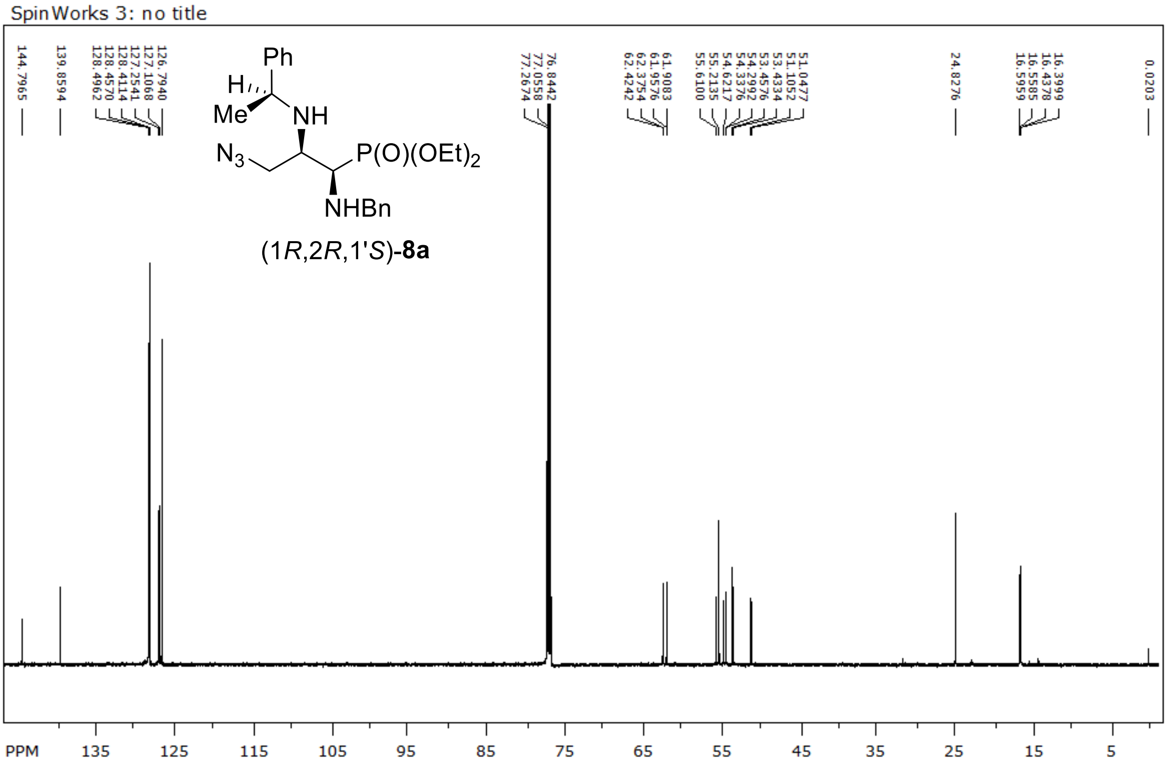

file: ...niany\(1R,2R,1'S)\abt-1242 13C\fid expt: <zgpg30>
transmitter freq.: 150.950591 MHz
time domain size: 65536 points
width: 36057.69 Hz = 238.8708 ppm = 0.550197 Hz/pt
number of scans: 4096

freq. of 0 ppm: 150.935497 MHz
processed size: 32768 complex points
LB: 0.000 GF: 0.0000
Hz/cm: 896.790 ppm/cm: 5.94095

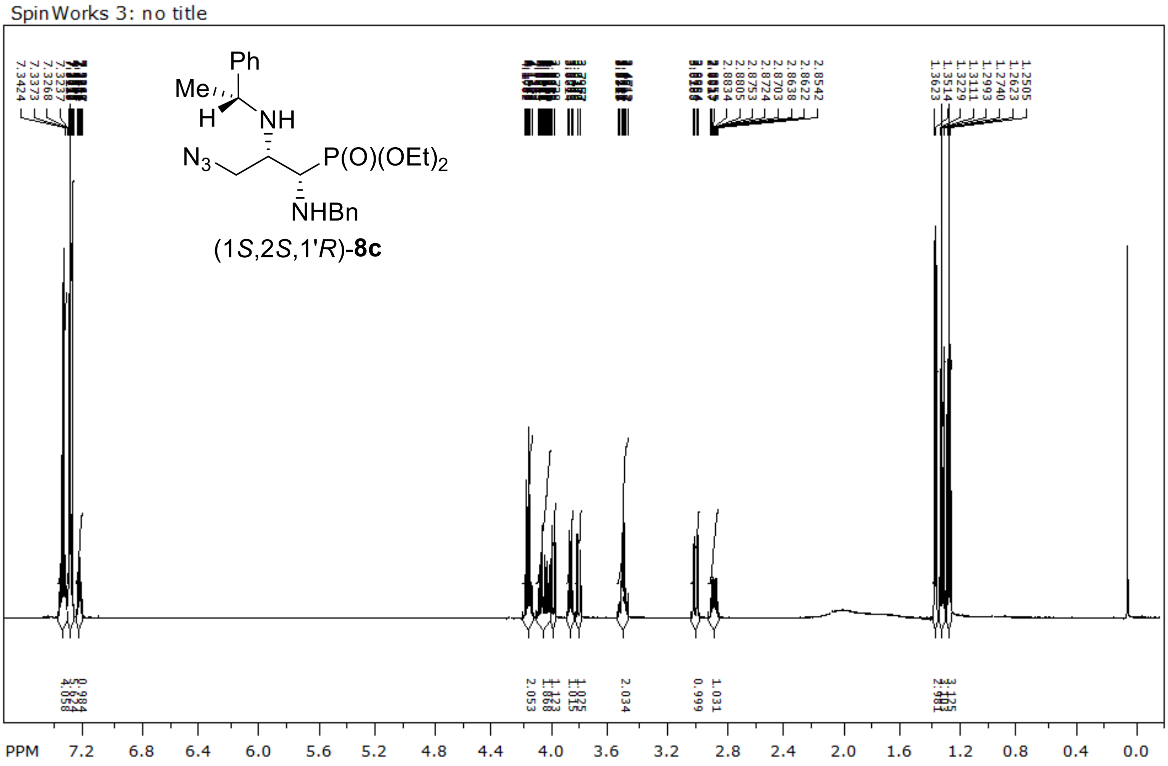

file: ...oniany\(1S,2S,1'R)\abt-0799 1H\fid expt: <zg30>
transmitter freq.: 600.263707 MHz
time domain size: 65536 points
width: 12335.53 Hz = 20.5502 ppm = 0.188225 Hz/pt
number of scans: 16

freq. of 0 ppm: 600.260000 MHz
processed size: 32768 complex points
LB: 0.000 GF: 0.0000
Hz/cm: 191.002 ppm/cm: 0.31820

SpinWorks 3: no title

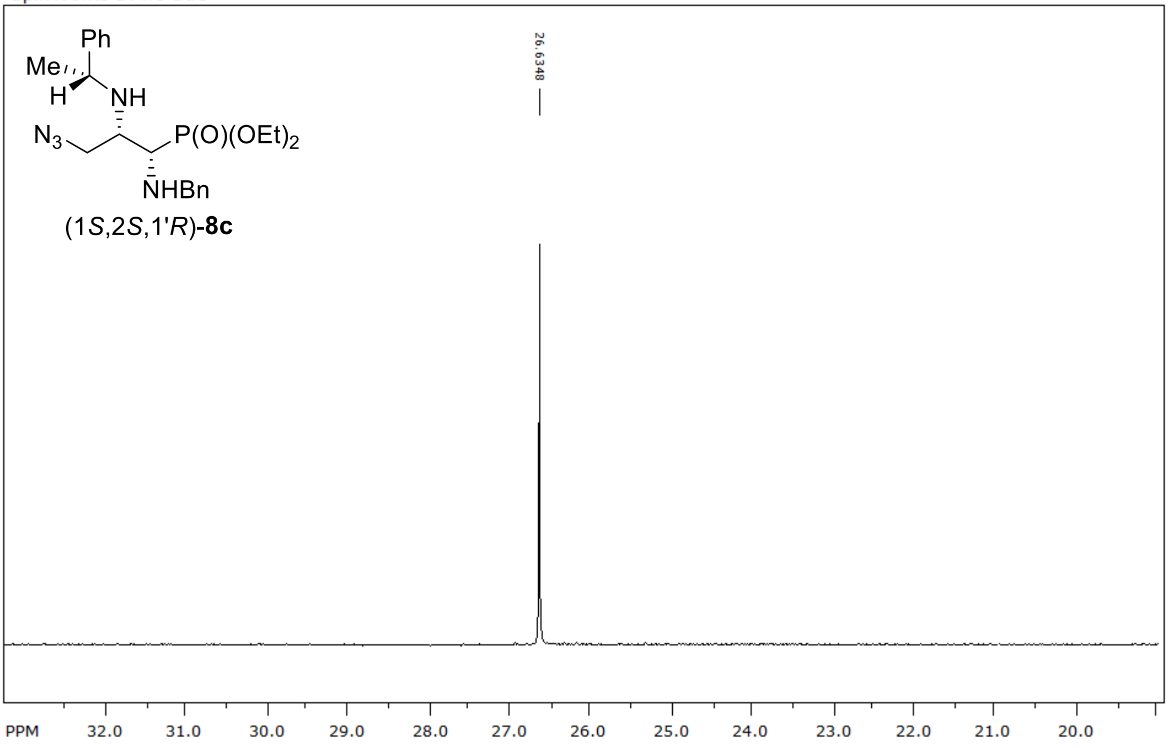

file: ...niany\(1S,2S,1'R)\abt-0799 31P\fid expt: <zgpg30>
transmitter freq.: 242.977552 MHz
time domain size: 65536 points
width: 96153.85 Hz = 395.7314 ppm = 1.467191 Hz/pt
number of scans: 128

freq. of 0 ppm: 242.989702 MHz
processed size: 32768 complex points
LB: 0.000 GF: 0.0000
Hz/cm: 139.578 ppm/cm: 0.57445

SpinWorks 3: no title

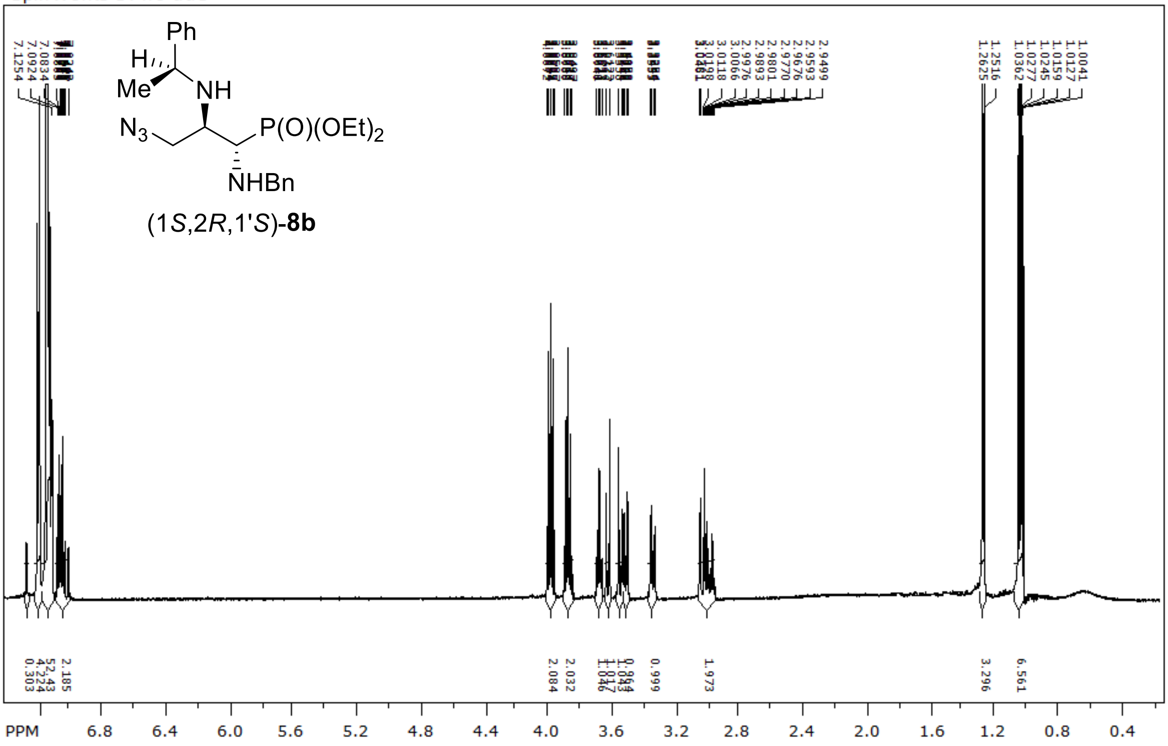

file: ...oniany\(1S,2R,1'S)\abt-1443 1H\fid expt: <zg30>
transmitter freq.: 600.263707 MHz
time domain size: 65536 points
width: 12335.53 Hz = 20.5502 ppm = 0.188225 Hz/pt
number of scans: 16

freq. of 0 ppm: 600.260000 MHz
processed size: 32768 complex points
LB: 0.000 GF: 0.0000
Hz/cm: 175.085 ppm/cm: 0.29168

SpinWorks 3: no title

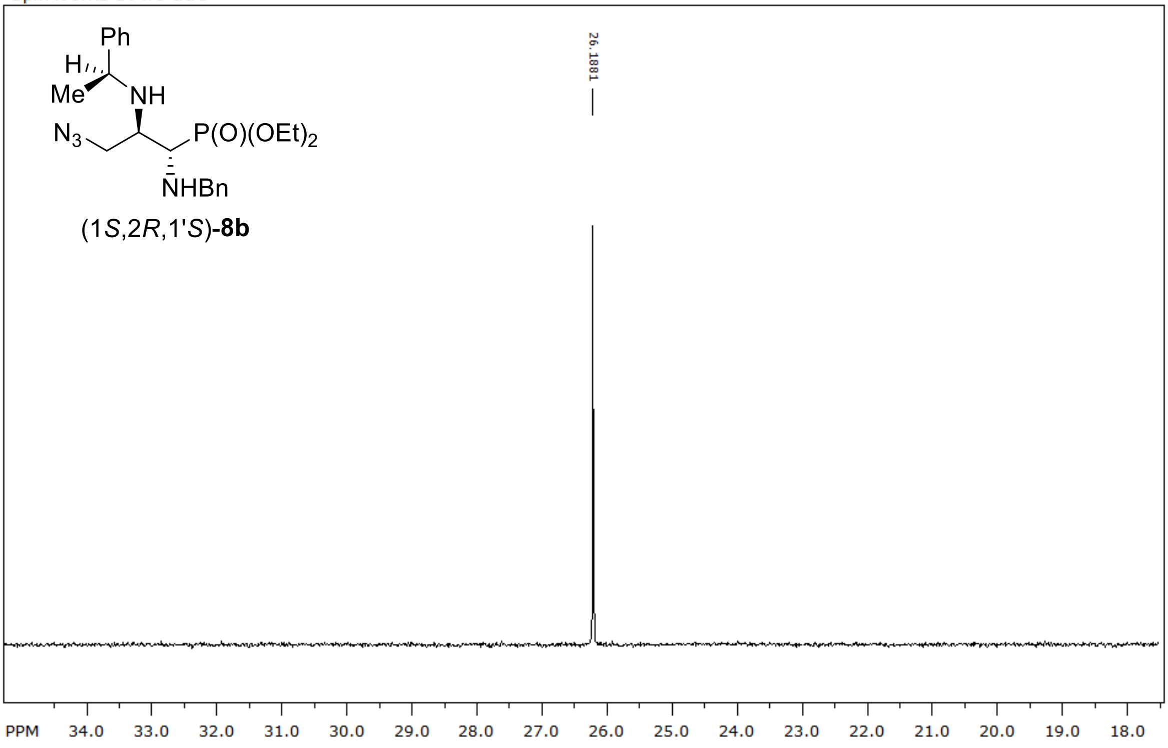

file: ...(1S,2R,1'S)\abt-0849 31P CDCl3\fid expt: <zgpg30>
transmitter freq.: 242.977552 MHz
time domain size: 65536 points
width: 96153.85 Hz = 395.7314 ppm = 1.467191 Hz/pt
number of scans: 16

freq. of 0 ppm: 242.989702 MHz
processed size: 32768 complex points
LB: 0.000 GF: 0.0000
Hz/cm: 173.697 ppm/cm: 0.71487

SpinWorks 3: no title

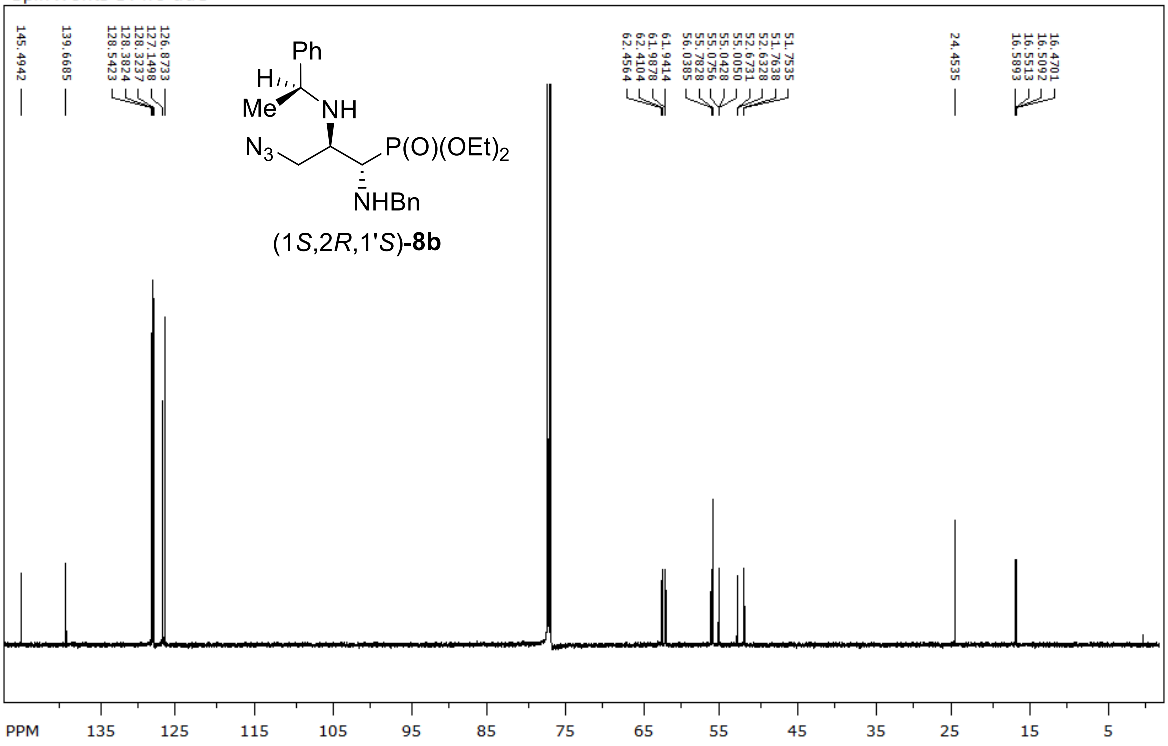

file: ...ny\(1S,2R,1'S)\abt-1059 13C\10\fid expt: <zgpg30>
transmitter freq.: 150.950591 MHz
time domain size: 65536 points
width: 36057.69 Hz = 238.8708 ppm = 0.550197 Hz/pt
number of scans: 2048

freq. of 0 ppm: 150.935497 MHz
processed size: 32768 complex points
LB: 0.000 GF: 0.0000
Hz/cm: 904.932 ppm/cm: 5.99489

SpinWorks 3: no title

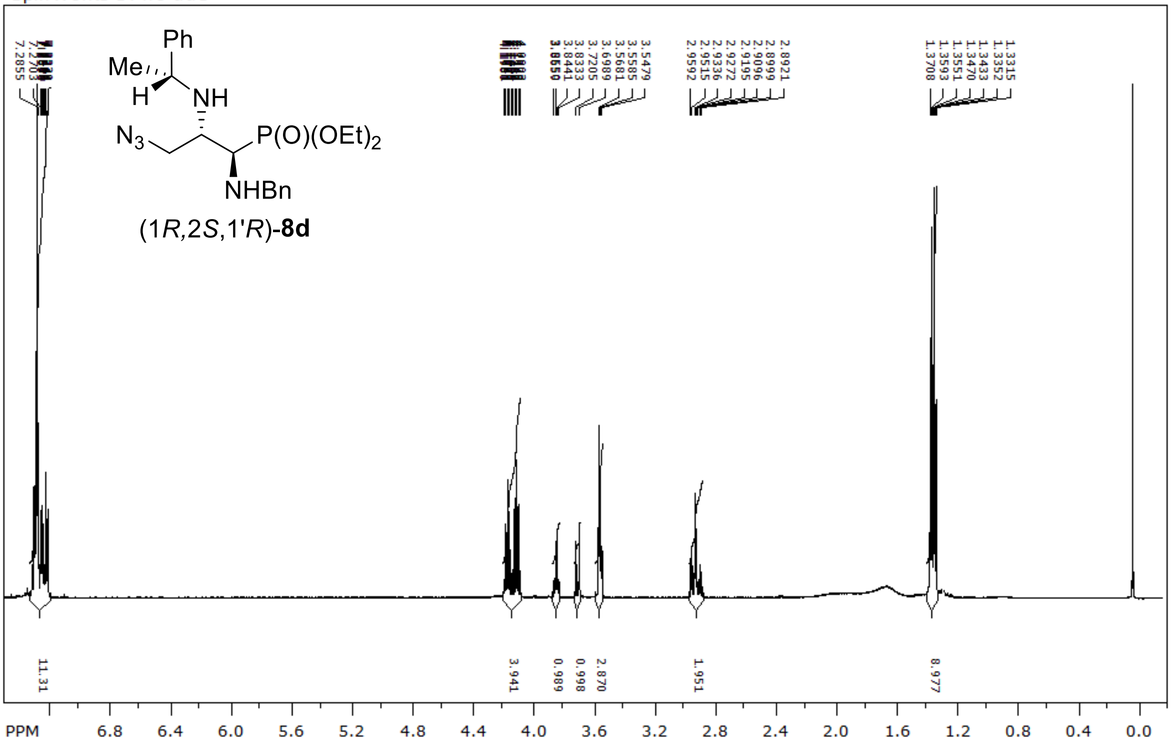

file: ...er\Desktop\NMR2019\abt-1407\10\fid   expt: <zg30>
transmitter freq.: 600.263707 MHz
time domain size: 65536 points
width: 12335.53 Hz = 20.5502 ppm = 0.188225 Hz/pt
number of scans: 16

freq. of 0 ppm: 600.260000 MHz
processed size: 32768 complex points
LB: 0.000   GF: 0.0000
Hz/cm: 184.635   ppm/cm: 0.30759

SpinWorks 3: no title

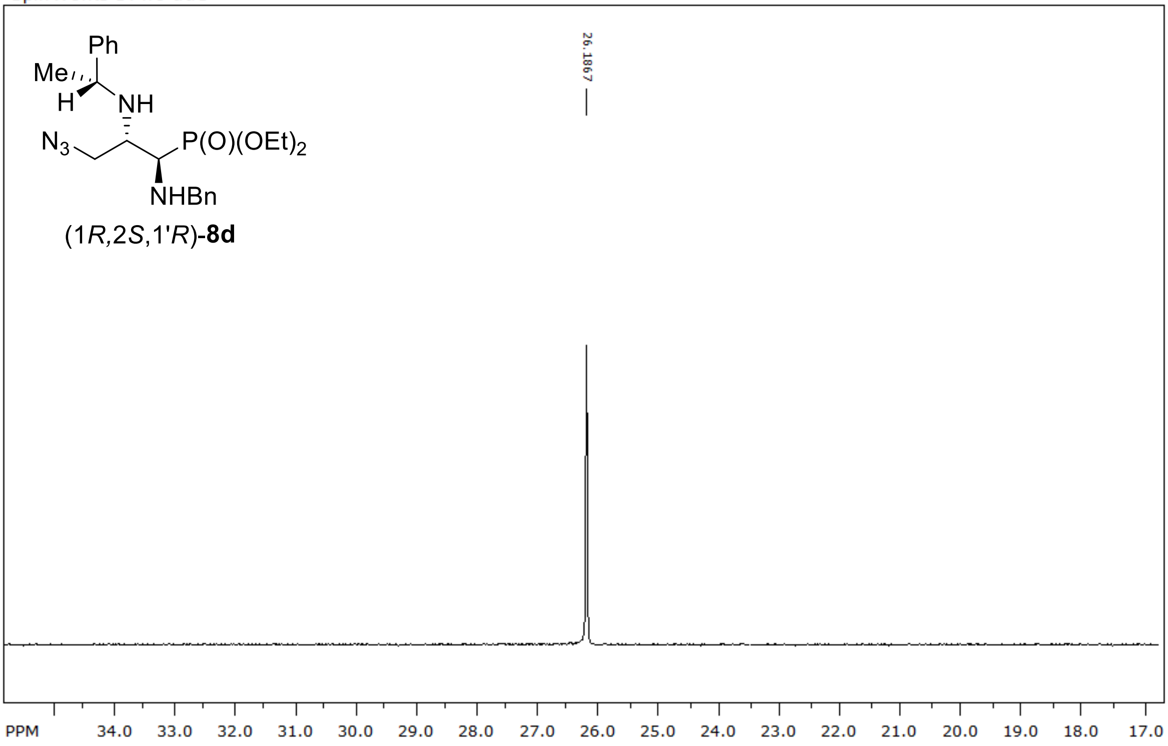

file: ...ktop\NMR\NMR2018UŁ\abt-0931\11\fid   expt: <zgpg30>
transmitter freq.: 242.977552 MHz
time domain size: 65536 points
width: 96153.85 Hz = 395.7314 ppm = 1.467191 Hz/pt
number of scans: 128

freq. of 0 ppm: 242.989702 MHz
processed size: 32768 complex points
LB: 0.000   GF: 0.0000
Hz/cm: 186.104   ppm/cm: 0.76593

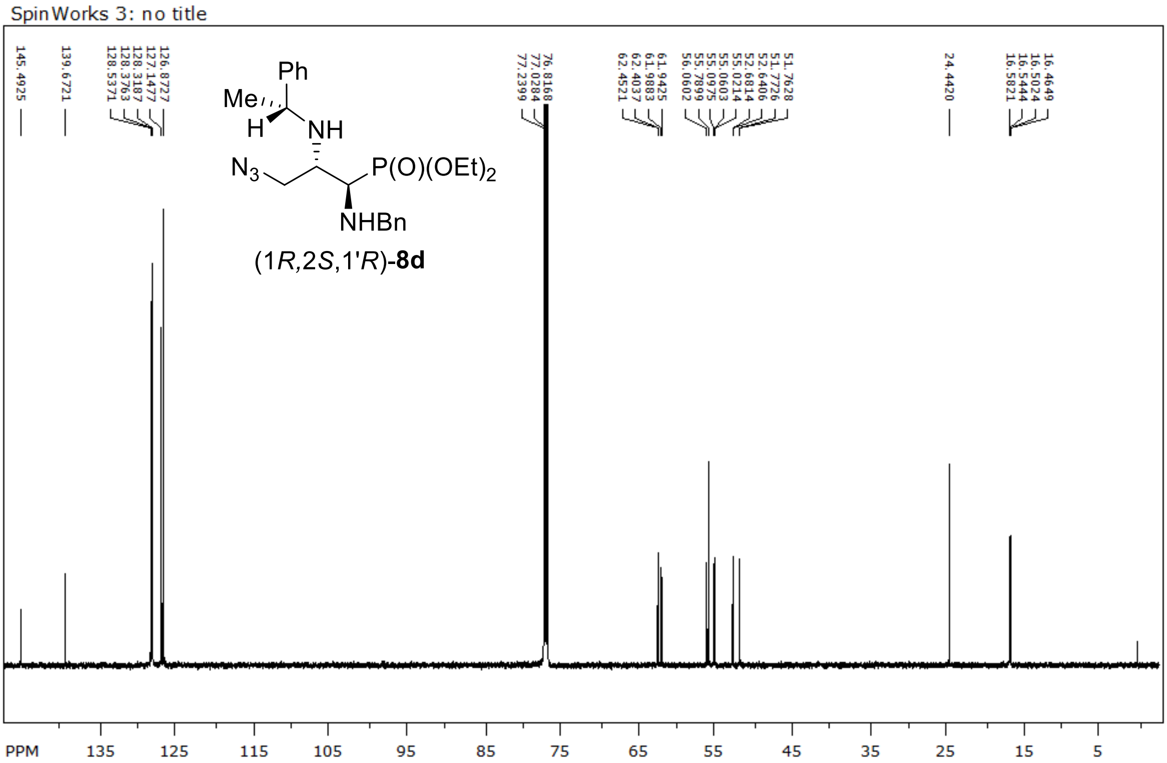

file: ...ny\(1R,2S,1'R)\abt-1012 13C\10\fid expt: <zgpg30>
transmitter freq.: 150.950591 MHz
time domain size: 65536 points
width: 36057.69 Hz = 238.8708 ppm = 0.550197 Hz/pt
number of scans: 3072

freq. of 0 ppm: 150.935497 MHz
processed size: 32768 complex points
LB: 0.000 GF: 0.0000
Hz/cm: 909.584 ppm/cm: 6.02571

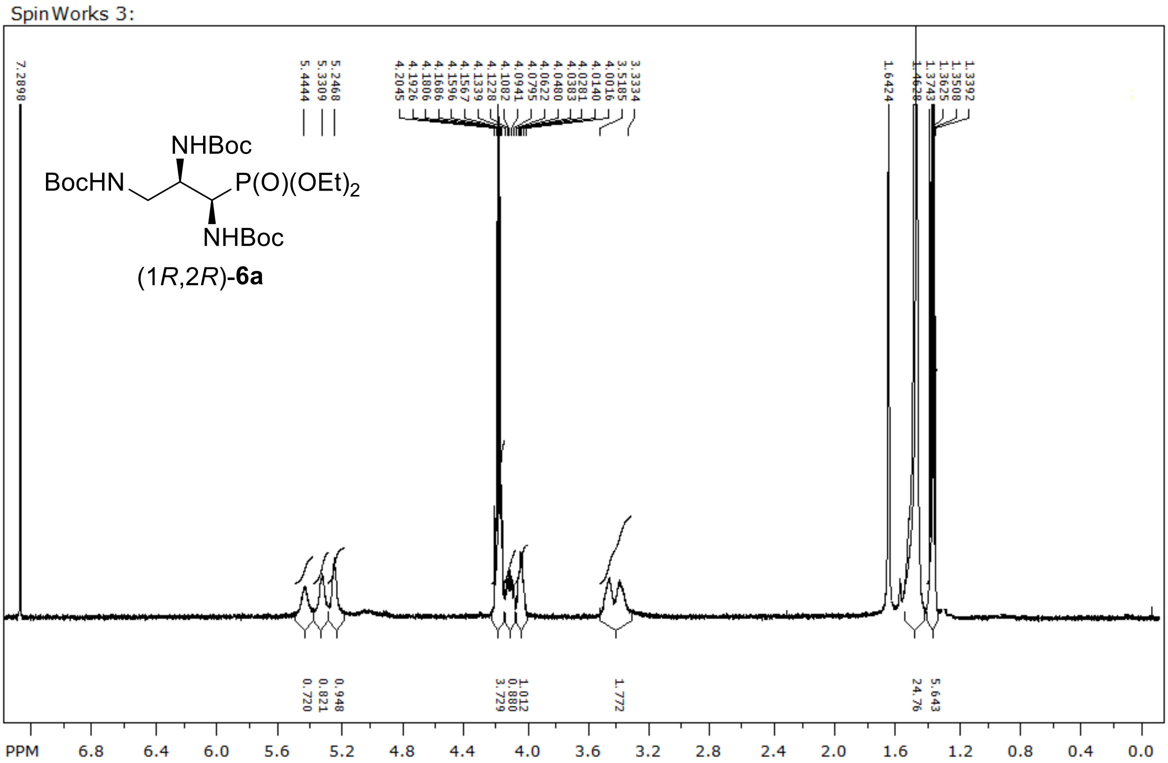

file: ...fosfoniany\(1R,2R)\abt-1040 1H\fid expt: <zg30>
transmitter freq.: 600.263707 MHz
time domain size: 65536 points
width: 12335.53 Hz = 20.5502 ppm = 0.188225 Hz/pt
number of scans: 16

freq. of 0 ppm: 600.260000 MHz
processed size: 32768 complex points
LB: 0.000 GF: 0.0000
Hz/cm: 180.656 ppm/cm: 0.30096

SpinWorks 3:

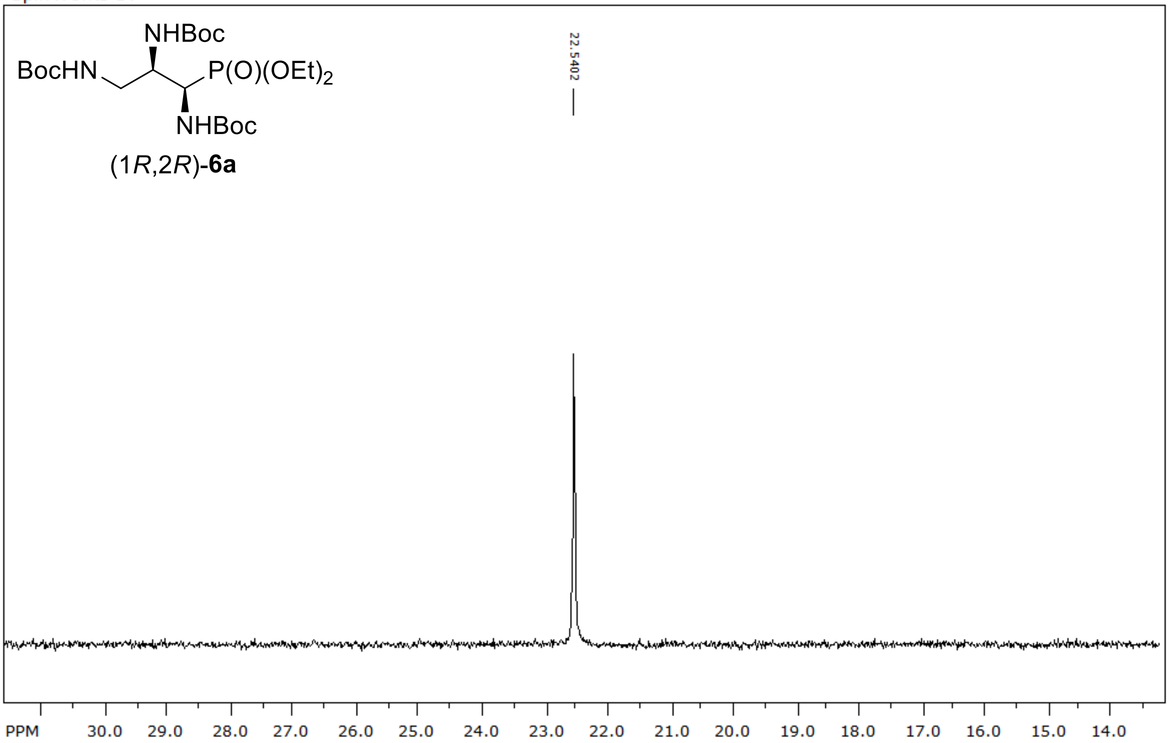

file: ...osfoniany\(1R,2R)\abt-1040 31P\fid expt: <zgpg30>
transmitter freq.: 242.977552 MHz
time domain size: 65536 points
width: 96153.85 Hz = 395.7314 ppm = 1.467191 Hz/pt
number of scans: 128

freq. of 0 ppm: 242.989702 MHz
processed size: 32768 complex points
LB: 0.000 GF: 0.0000
Hz/cm: 179.901 ppm/cm: 0.74040

SpinWorks 3: no title

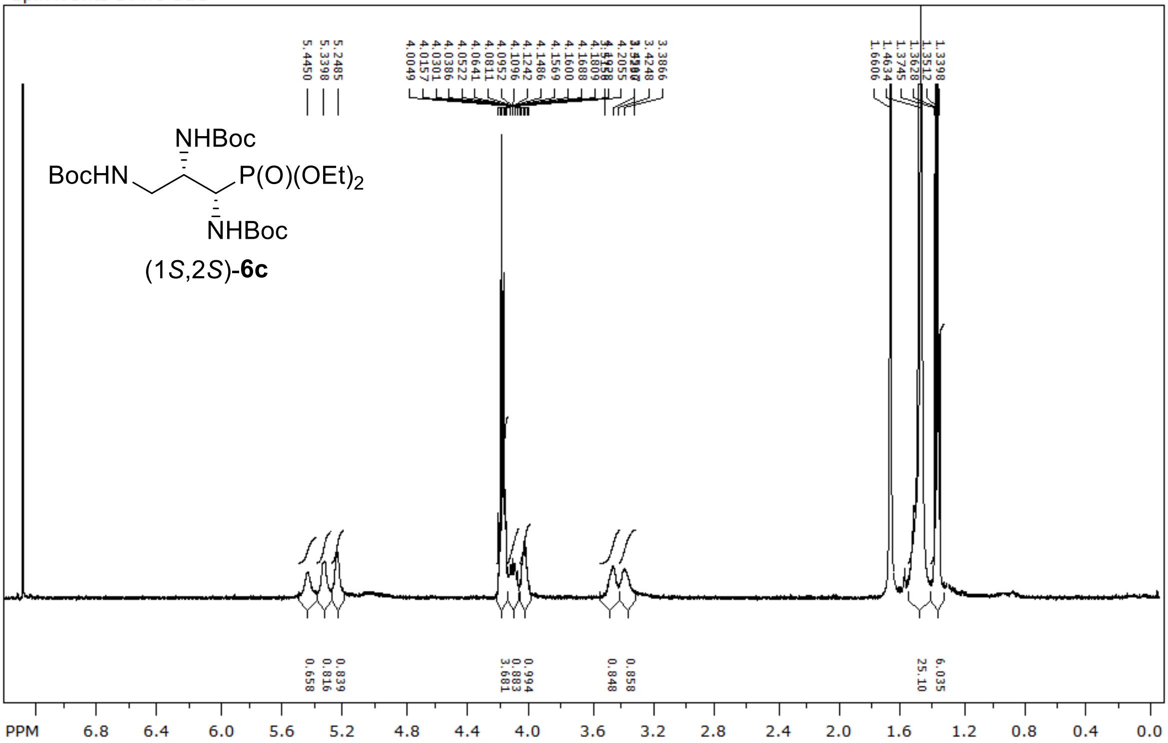

file: ...fosfoniany\(1S,2S)\abt-1046 1H\fid expt: <zg30>
transmitter freq.: 600.263707 MHz
time domain size: 65536 points
width: 12335.53 Hz = 20.5502 ppm = 0.188225 Hz/pt
number of scans: 16

freq. of 0 ppm: 600.260000 MHz
processed size: 32768 complex points
LB: 0.000 GF: 0.0000
Hz/cm: 180.258 ppm/cm: 0.30030

SpinWorks 3: no title

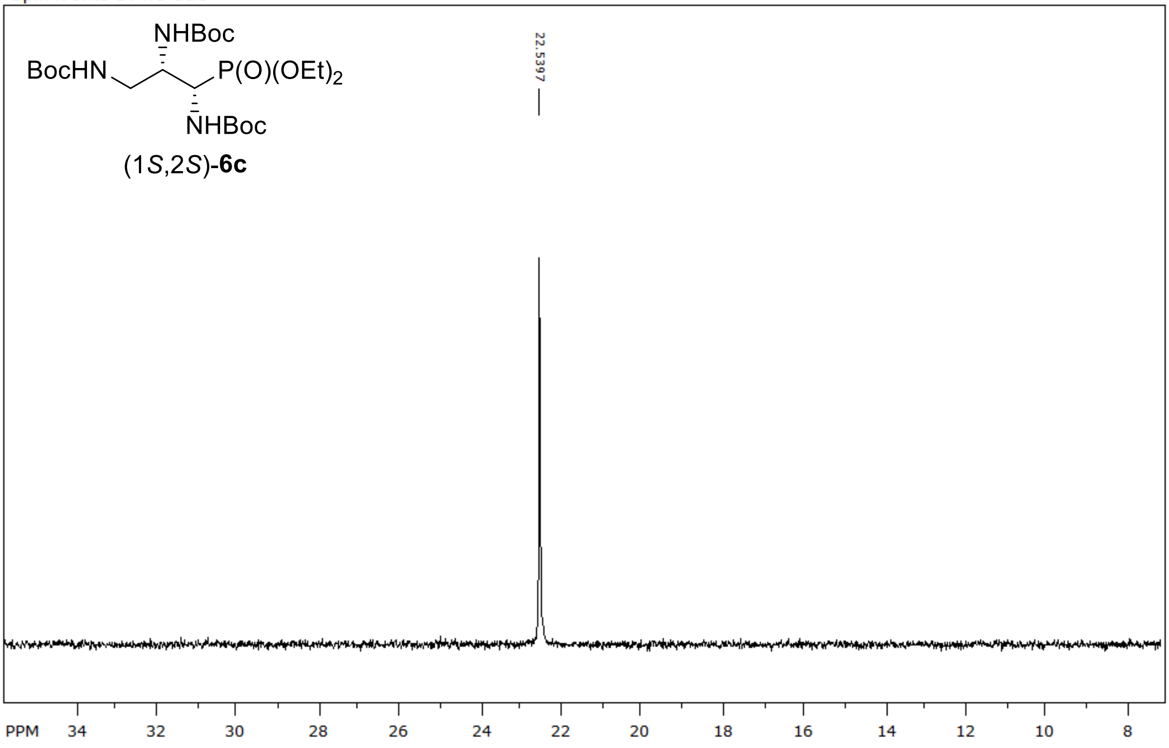

file: ...osfoniany\(1S,2S)\abt-1046 31P\fid expt: <zgpg30>
transmitter freq.: 242.977552 MHz
time domain size: 65536 points
width: 96153.85 Hz = 395.7314 ppm = 1.467191 Hz/pt
number of scans: 128

freq. of 0 ppm: 242.989702 MHz
processed size: 32768 complex points
LB: 0.000 GF: 0.0000
Hz/cm: 279.156 ppm/cm: 1.14890

SpinWorks 3: no title

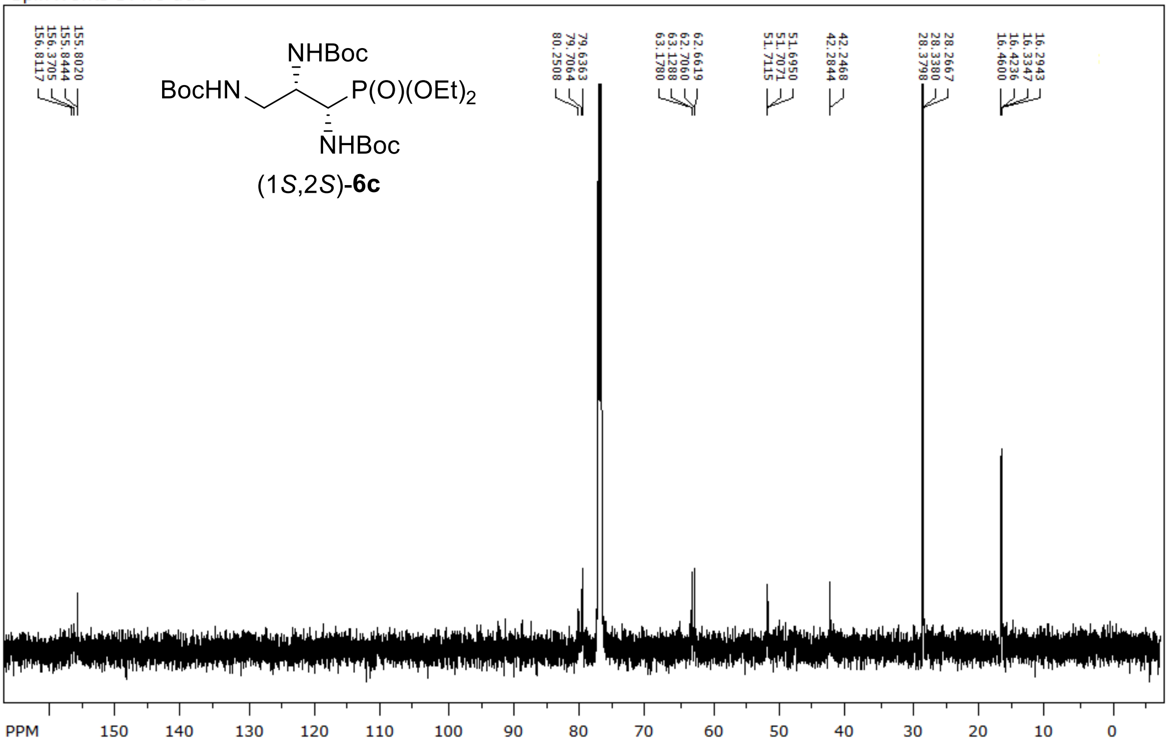

file: ...osfoniany\(1S,2S)\abt-1046 13C\fid expt: <zgpg30>
transmitter freq.: 150.950591 MHz
time domain size: 65536 points
width: 36057.69 Hz = 238.8708 ppm = 0.550197 Hz/pt
number of scans: 7000

freq. of 0 ppm: 150.935497 MHz
processed size: 32768 complex points
LB: 0.000 GF: 0.0000
Hz/cm: 1053.815 ppm/cm: 6.98119

SpinWorks 3:

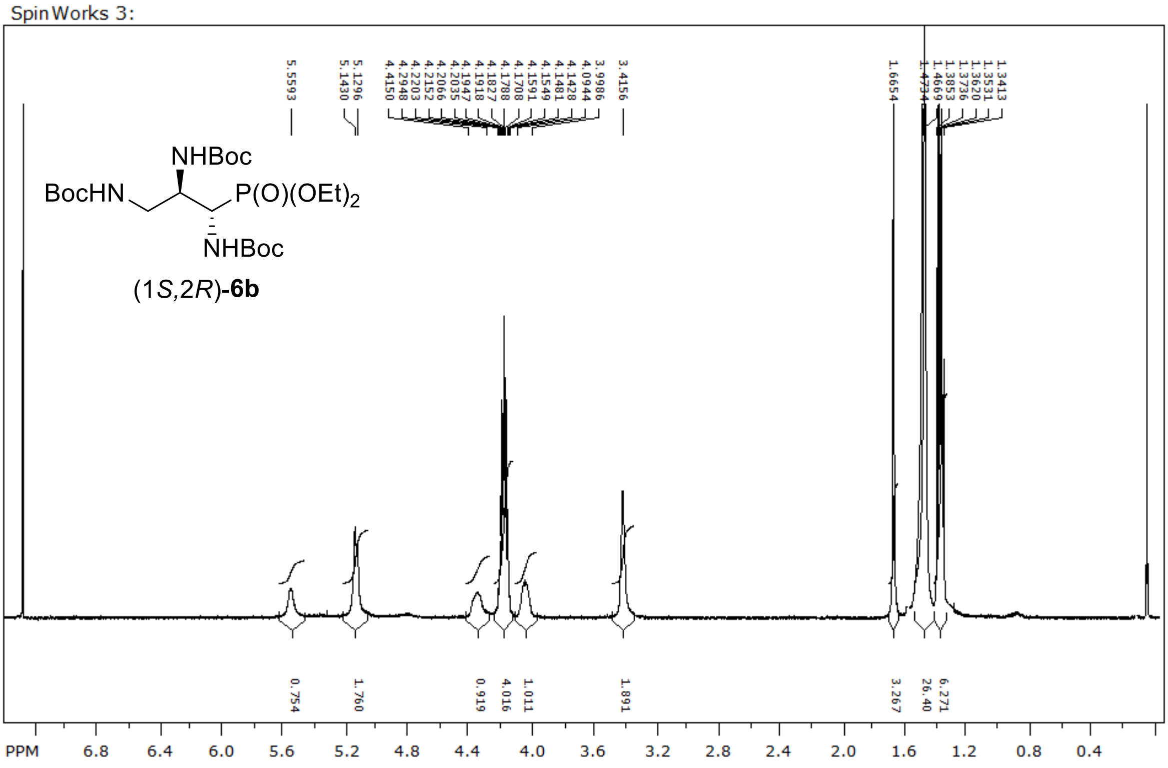

file: ...fosfoniany\(1S,2R)\abt-1051 1H\fid expt: <zg30>
transmitter freq.: 600.263707 MHz
time domain size: 65536 points
width: 12335.53 Hz = 20.5502 ppm = 0.188225 Hz/pt
number of scans: 16

freq. of 0 ppm: 600.260000 MHz
processed size: 32768 complex points
LB: 0.000 GF: 0.0000
Hz/cm: 179.462 ppm/cm: 0.29897

SpinWorks 3:

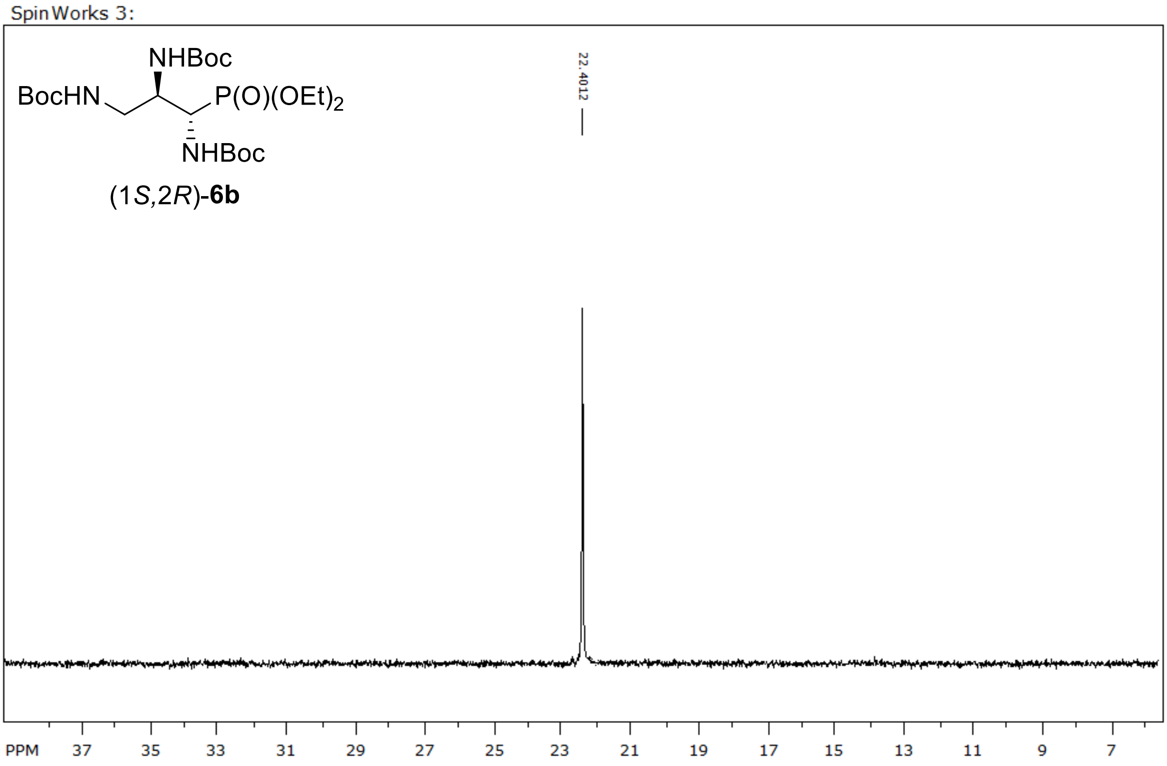

file: ...osfoniany\(1S,2R)\abt-1051 31P\fid expt: <zgpg30>
transmitter freq.: 242.977552 MHz
time domain size: 65536 points
width: 96153.85 Hz = 395.7314 ppm = 1.467191 Hz/pt
number of scans: 128

freq. of 0 ppm: 242.989702 MHz
processed size: 32768 complex points
LB: 0.000 GF: 0.0000
Hz/cm: 328.784 ppm/cm: 1.35315

SpinWorks 3: no title

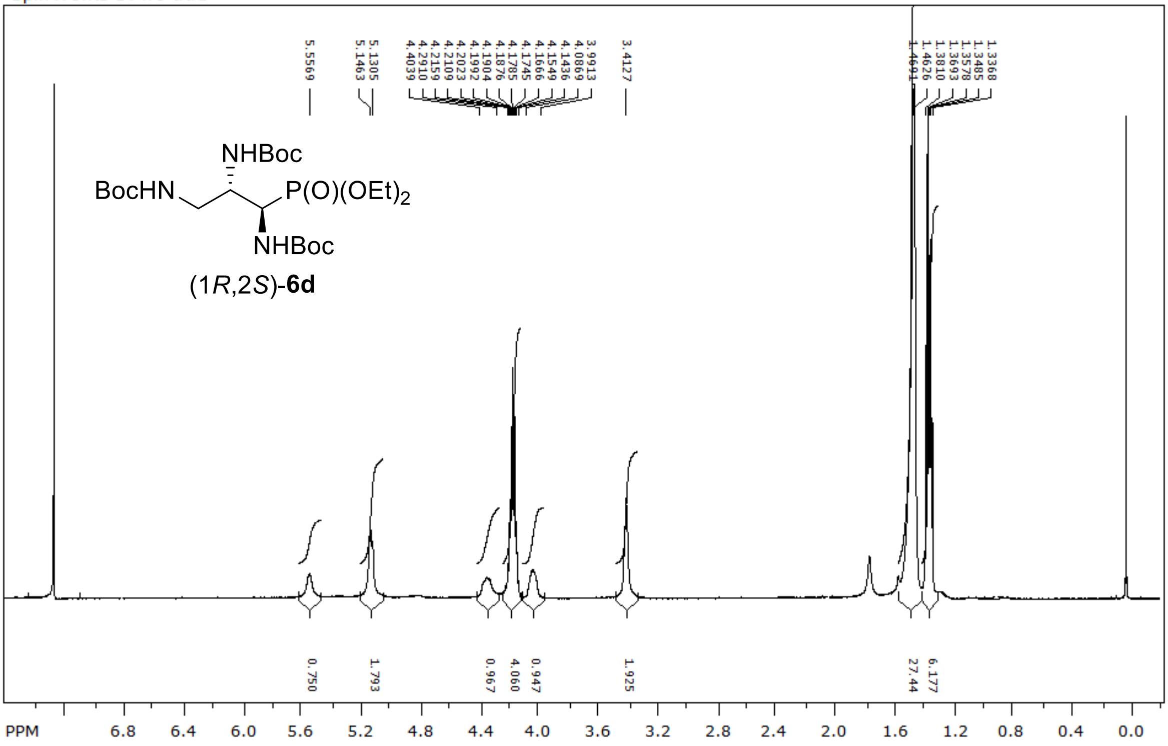

file: ...fosfoniany\(1R,2S)\abt-1065 1H\fid   expt: <zg30>
transmitter freq.: 600.263707 MHz
time domain size: 65536 points
width: 12335.53 Hz = 20.5502 ppm = 0.188225 Hz/pt
number of scans: 16

freq. of 0 ppm: 600.260000 MHz
processed size: 32768 complex points
LB: 0.000   GF: 0.0000
Hz/cm: 188.216   ppm/cm: 0.31356

SpinWorks 3: no title

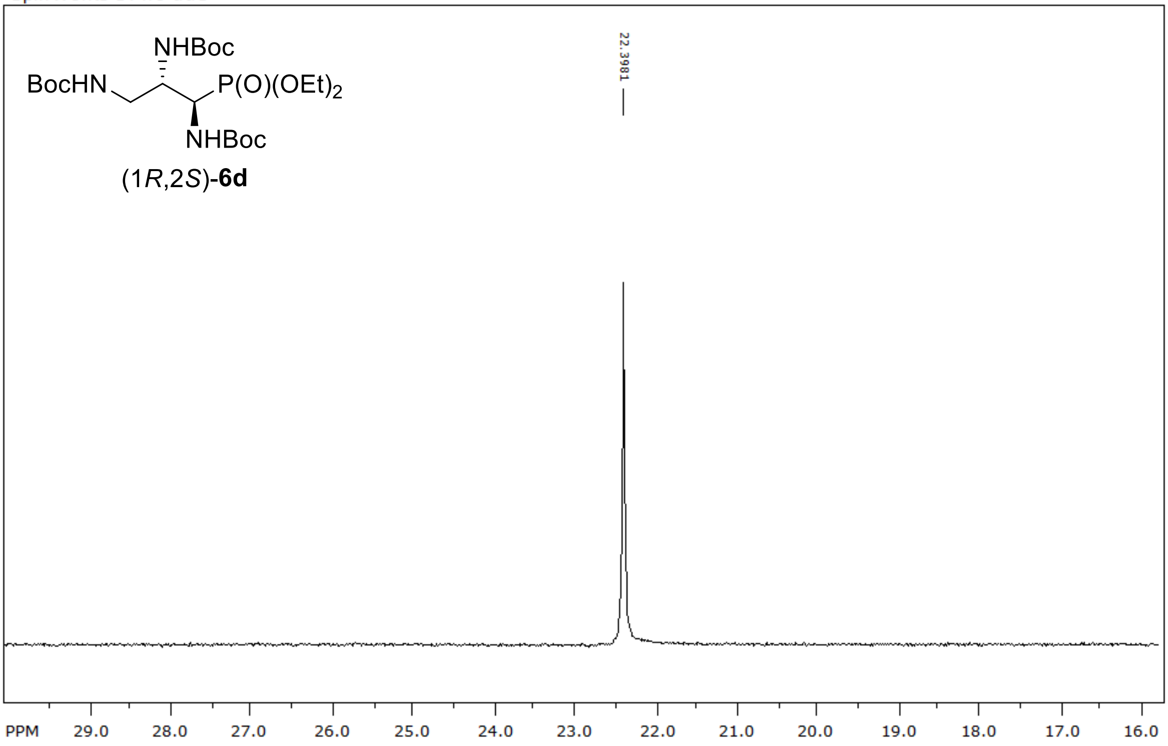

file: ...osfoniany\(1R,2S)\abt-1065 31P\fid   expt: <zgpg30>
transmitter freq.: 242.977552 MHz
time domain size: 65536 points
width: 96153.85 Hz = 395.7314 ppm = 1.467191 Hz/pt
number of scans: 128

freq. of 0 ppm: 242.989702 MHz
processed size: 32768 complex points
LB: 0.000   GF: 0.0000
Hz/cm: 139.578   ppm/cm: 0.57445

SpinWorks 3: no title

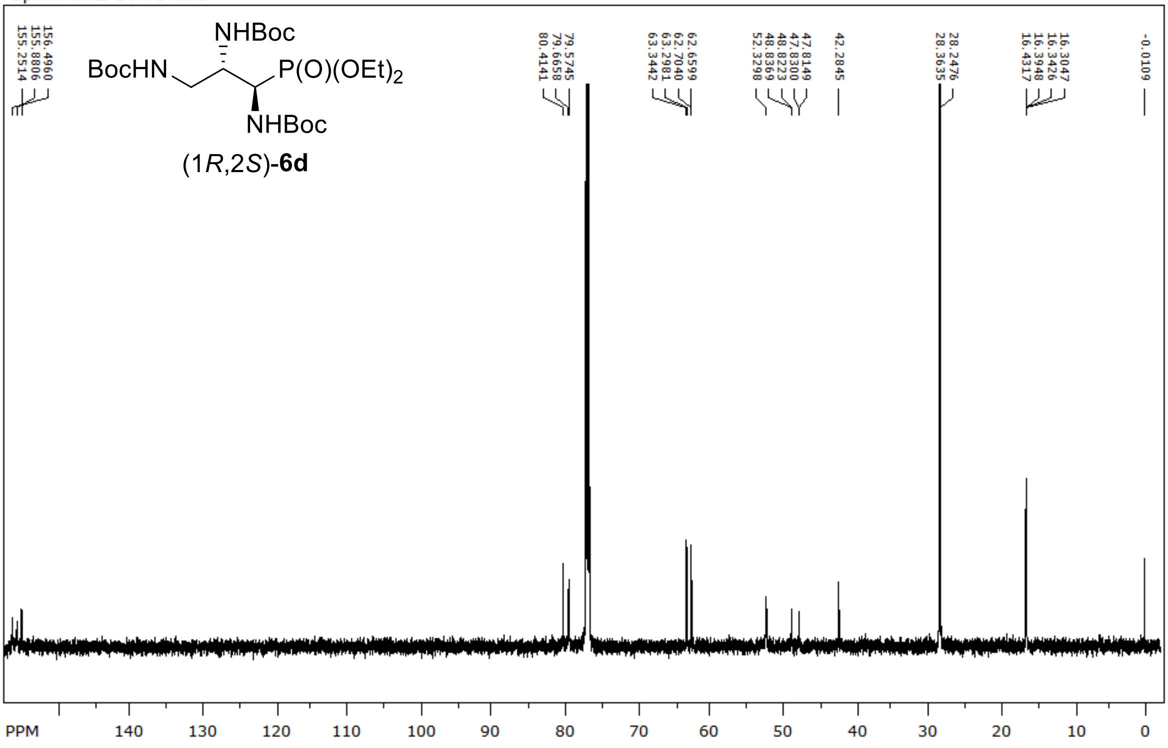

file: ...osfoniany\(1R,2S)\abt-1065 13C\fid expt: <zgpg30>
transmitter freq.: 150.950591 MHz
time domain size: 65536 points
width: 36057.69 Hz = 238.8708 ppm = 0.550197 Hz/pt
number of scans: 4000

freq. of 0 ppm: 150.935497 MHz
processed size: 32768 complex points
LB: 0.000 GF: 0.0000
Hz/cm: 965.416 ppm/cm: 6.39557

SpinWorks 3: no title

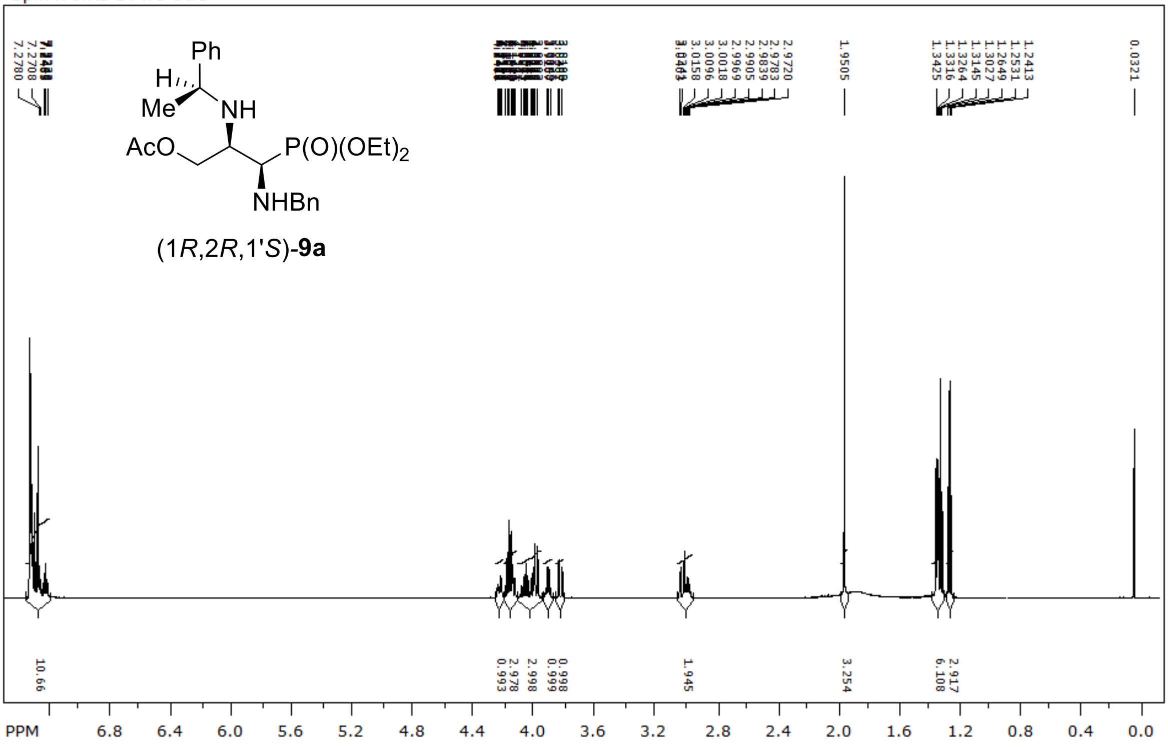

file: ...\(1R,2R,1'S)\abt-0784 1H CDCl3\fid expt: <zg30>
transmitter freq.: 600.263707 MHz
time domain size: 65536 points
width: 12335.53 Hz = 20.5502 ppm = 0.188225 Hz/pt
number of scans: 16

freq. of 0 ppm: 600.260000 MHz
processed size: 32768 complex points
LB: 0.000 GF: 0.0000
Hz/cm: 183.839 ppm/cm: 0.30626

SpinWorks 3:

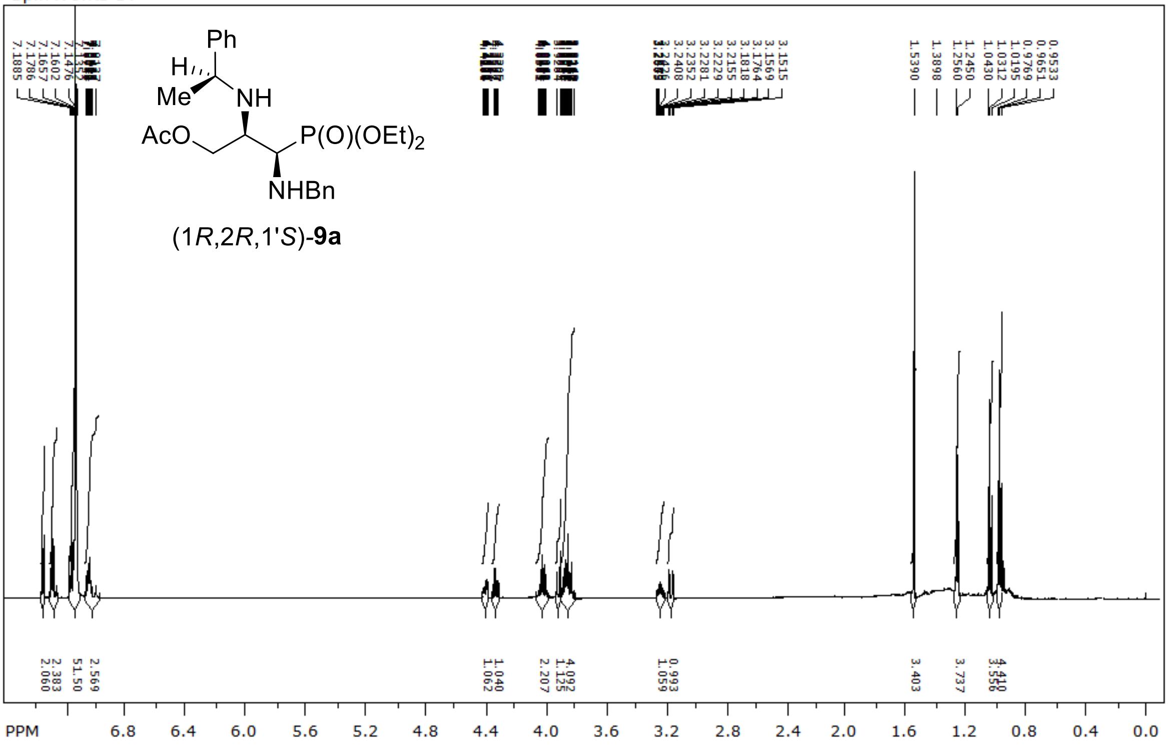

file: ...sktop\NMR\NMR2017UŁ\abt-0488\1\fid expt: <zg30>
transmitter freq.: 600.263707 MHz
time domain size: 65536 points
width: 12335.53 Hz = 20.5502 ppm = 0.188225 Hz/pt
number of scans: 16

freq. of 0 ppm: 600.260000 MHz
processed size: 32768 complex points
LB: 0.000 GF: 0.0000
Hz/cm: 185.829 ppm/cm: 0.30958

SpinWorks 3: no title

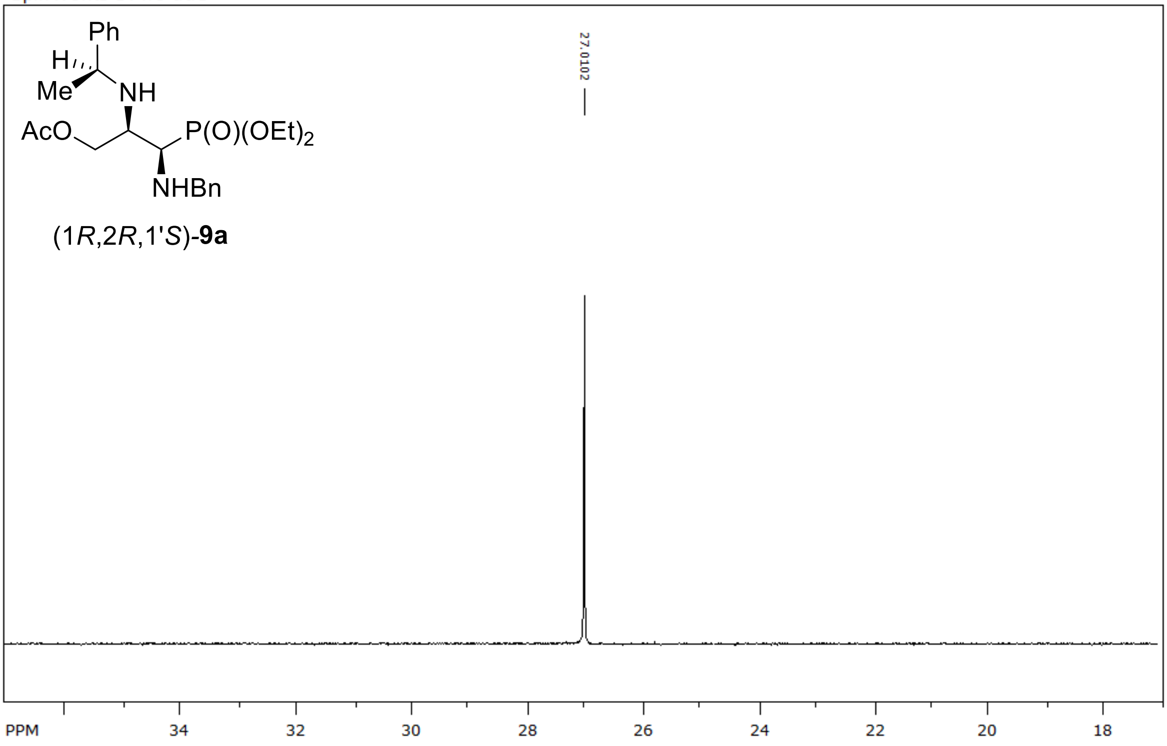

file: ...hodne\(1R,2R,1'S)\abt-0784 31P\fid expt: <zgpg30>
transmitter freq.: 242.977552 MHz
time domain size: 65536 points
width: 96153.85 Hz = 395.7314 ppm = 1.467191 Hz/pt
number of scans: 128

freq. of 0 ppm: 242.989702 MHz
processed size: 32768 complex points
LB: 0.000 GF: 0.0000
Hz/cm: 195.409 ppm/cm: 0.80423

SpinWorks 3: no title

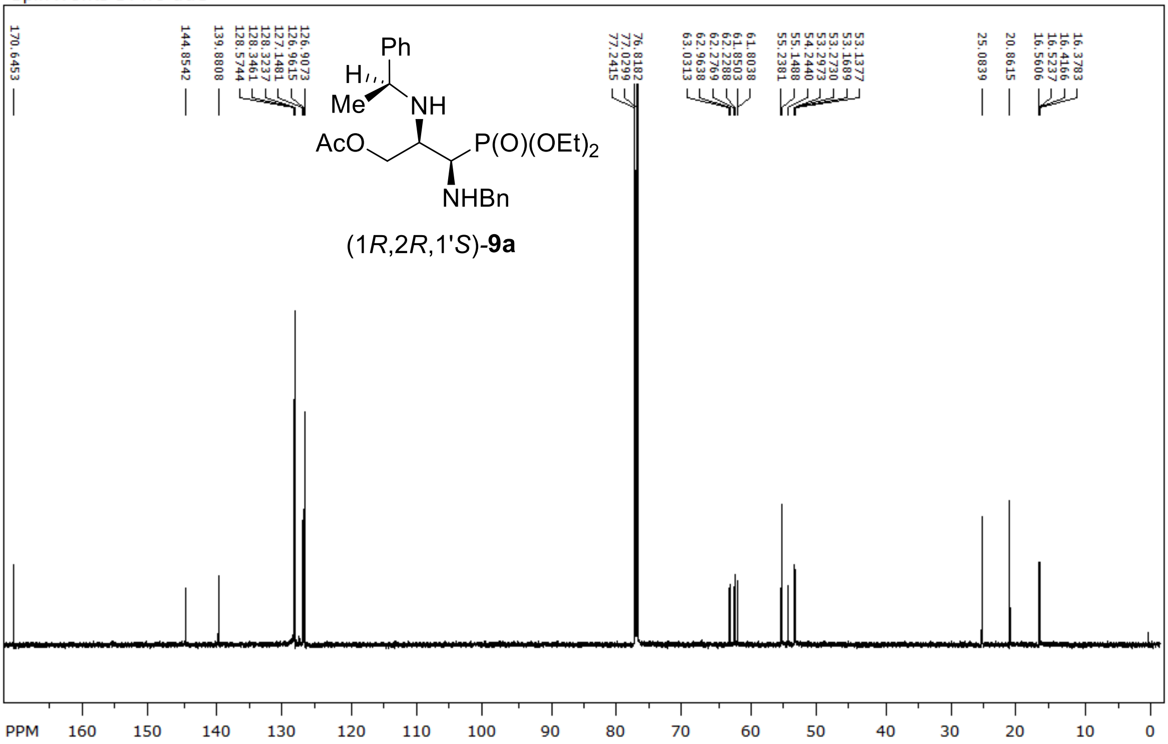

file: ...hodne\(1R,2R,1'S)\abt-0243 13C\fid expt: <zgpg30>
transmitter freq.: 150.950591 MHz
time domain size: 65536 points
width: 36057.69 Hz = 238.8708 ppm = 0.550197 Hz/pt
number of scans: 2048

freq. of 0 ppm: 150.935497 MHz
processed size: 32768 complex points
LB: 0.000 GF: 0.0000
Hz/cm: 1050.326 ppm/cm: 6.95808

SpinWorks 3: no title

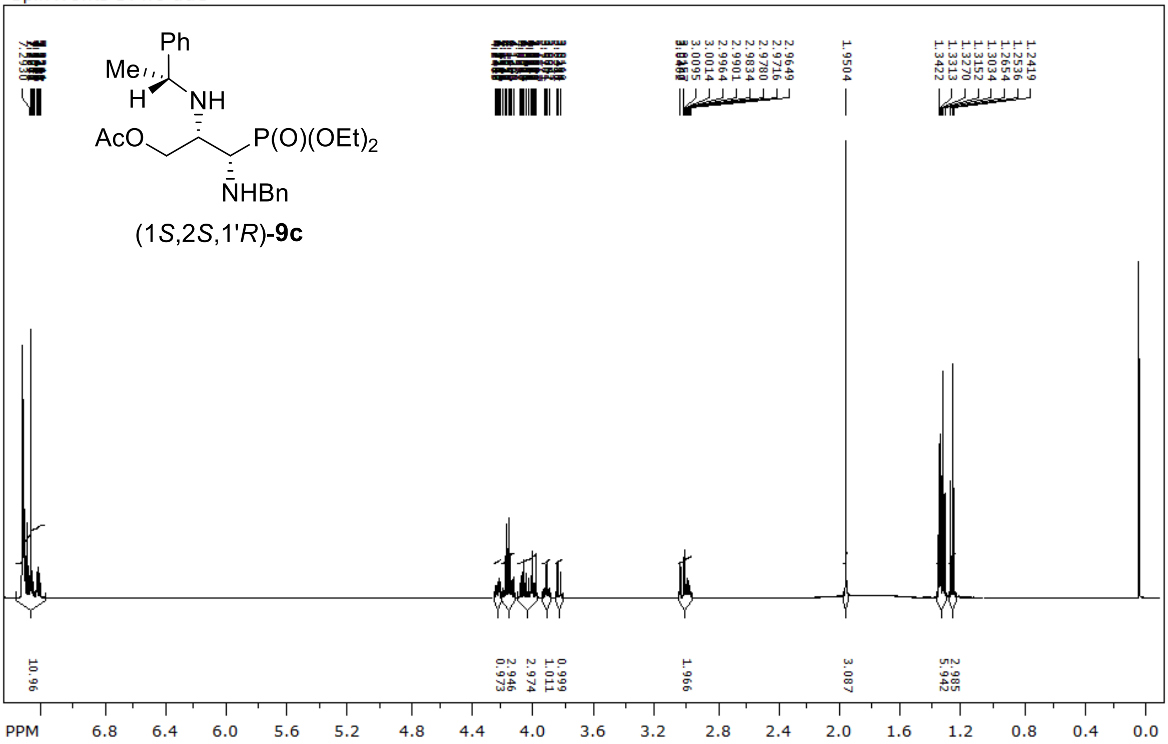

file: ...chodne\(1S,2S,1'R)\abt-0753 1H\fid expt: <zg30>
transmitter freq.: 600.263707 MHz
time domain size: 65536 points
width: 12335.53 Hz = 20.5502 ppm = 0.188225 Hz/pt
number of scans: 16

freq. of 0 ppm: 600.260000 MHz
processed size: 32768 complex points
LB: 0.000 GF: 0.0000
Hz/cm: 181.850 ppm/cm: 0.30295

SpinWorks 3: no title

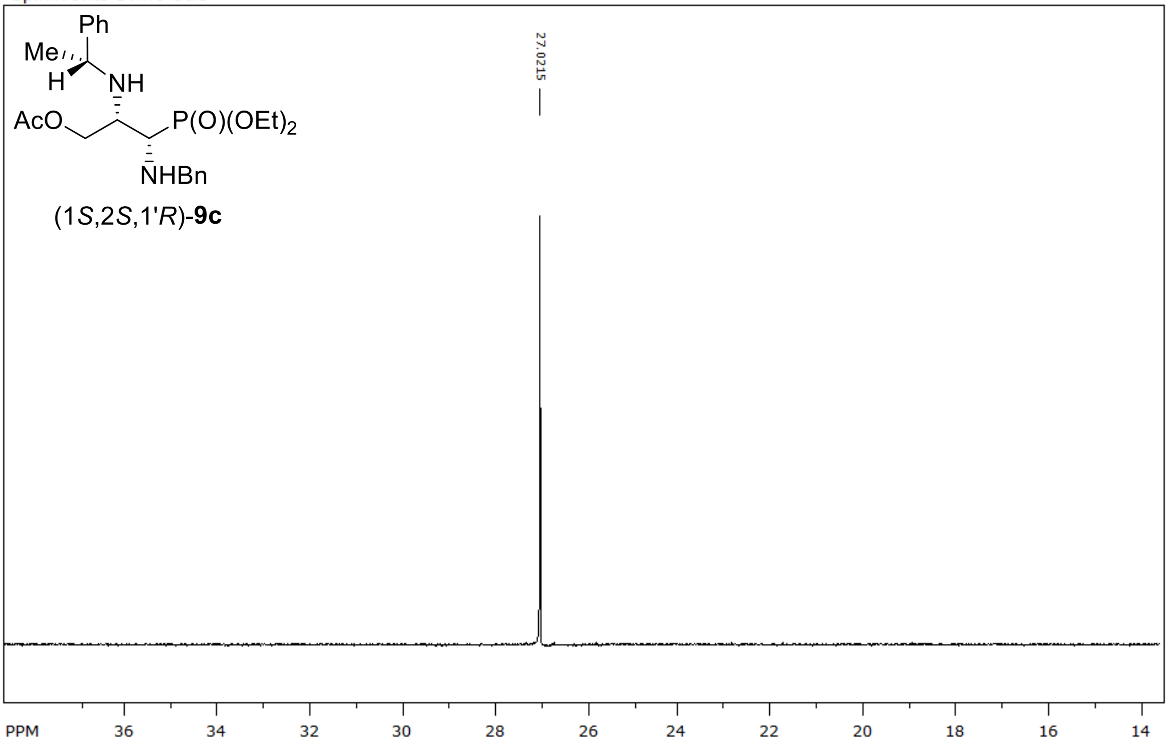

file: ...hodne\(1S,2S,1'R)\abt-0753 31P\fid expt: <zgpg30>
transmitter freq.: 242.977552 MHz
time domain size: 65536 points
width: 96153.85 Hz = 395.7314 ppm = 1.467191 Hz/pt
number of scans: 128

freq. of 0 ppm: 242.989702 MHz
processed size: 32768 complex points
LB: 0.000 GF: 0.0000
Hz/cm: 245.037 ppm/cm: 1.00848

SpinWorks 3:

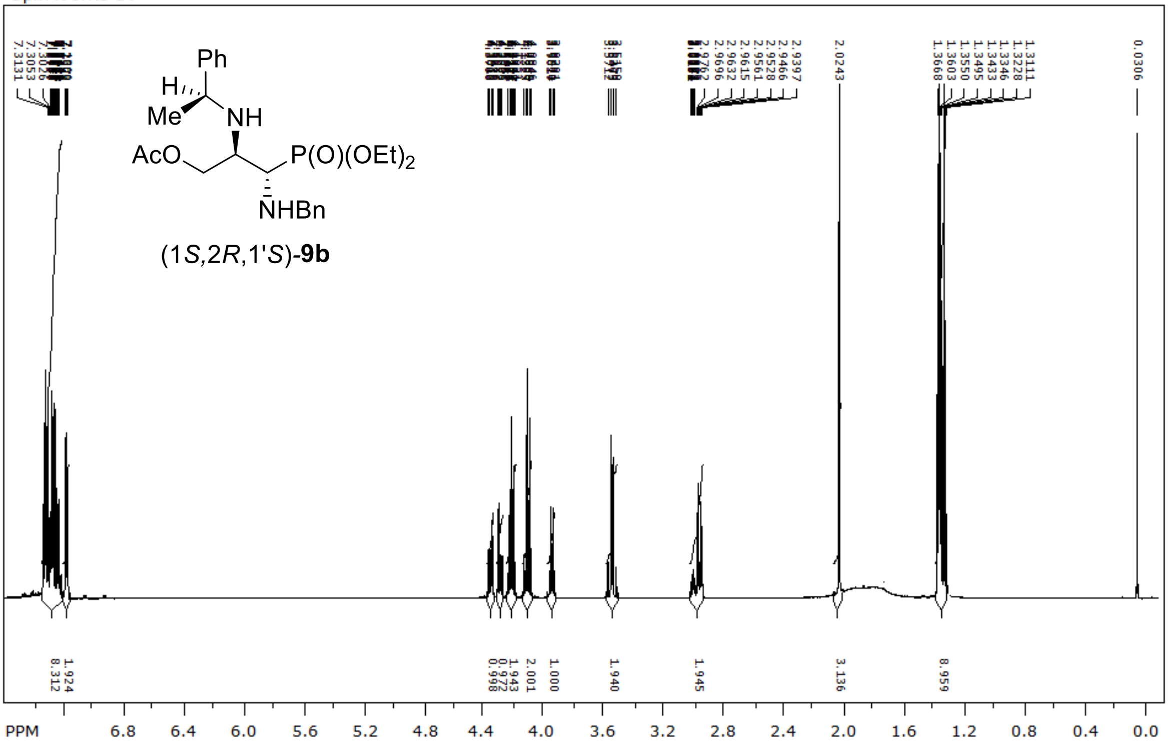

file: ...chodne\(1S,2R,1'S)\abt-1031 1H\fid expt: <zg30>
transmitter freq.: 600.263707 MHz
time domain size: 65536 points
width: 12335.53 Hz = 20.5502 ppm = 0.188225 Hz/pt
number of scans: 16

freq. of 0 ppm: 600.260000 MHz
processed size: 32768 complex points
LB: 0.000 GF: 0.0000
Hz/cm: 185.829 ppm/cm: 0.30958

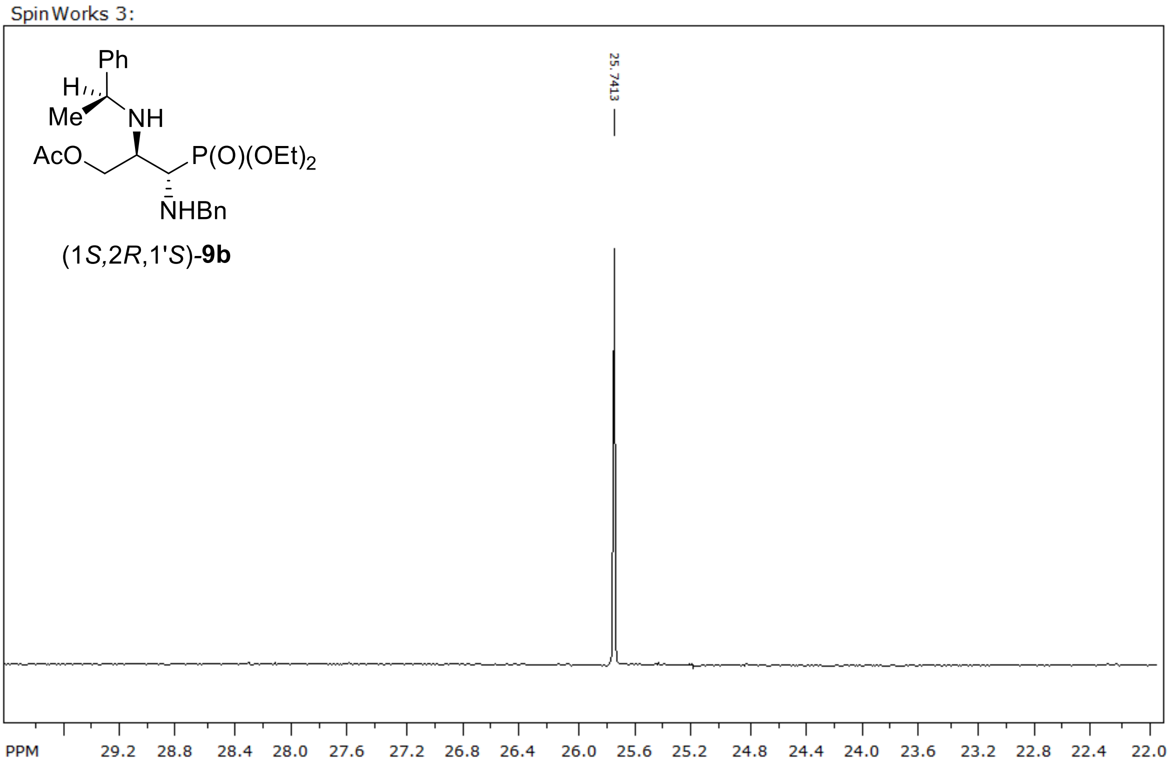

file: ...hodne\(1S,2R,1'S)\abt-1031 31P\fid expt: <zgpg30>
transmitter freq.: 242.977552 MHz
time domain size: 65536 points
width: 96153.85 Hz = 395.7314 ppm = 1.467191 Hz/pt
number of scans: 128

freq. of 0 ppm: 242.989702 MHz
processed size: 32768 complex points
LB: 0.000 GF: 0.0000
Hz/cm: 78.917 ppm/cm: 0.32479

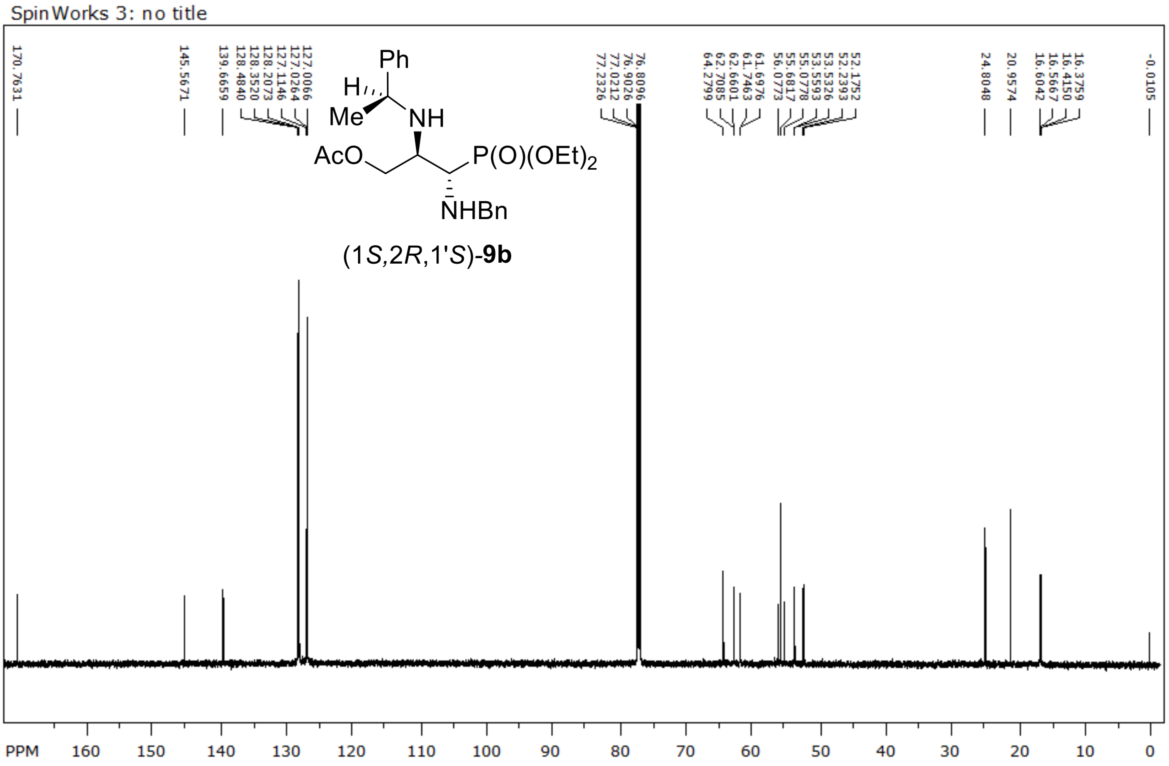

file: ...hodne\(1S,2R,1'S)\abt-0314 13C\fid expt: <zgpg30>
transmitter freq.: 150.950591 MHz
time domain size: 65536 points
width: 36057.69 Hz = 238.8708 ppm = 0.550197 Hz/pt
number of scans: 3072

freq. of 0 ppm: 150.935497 MHz
processed size: 32768 complex points
LB: 0.000 GF: 0.0000
Hz/cm: 1052.652 ppm/cm: 6.97349

SpinWorks 3: no title

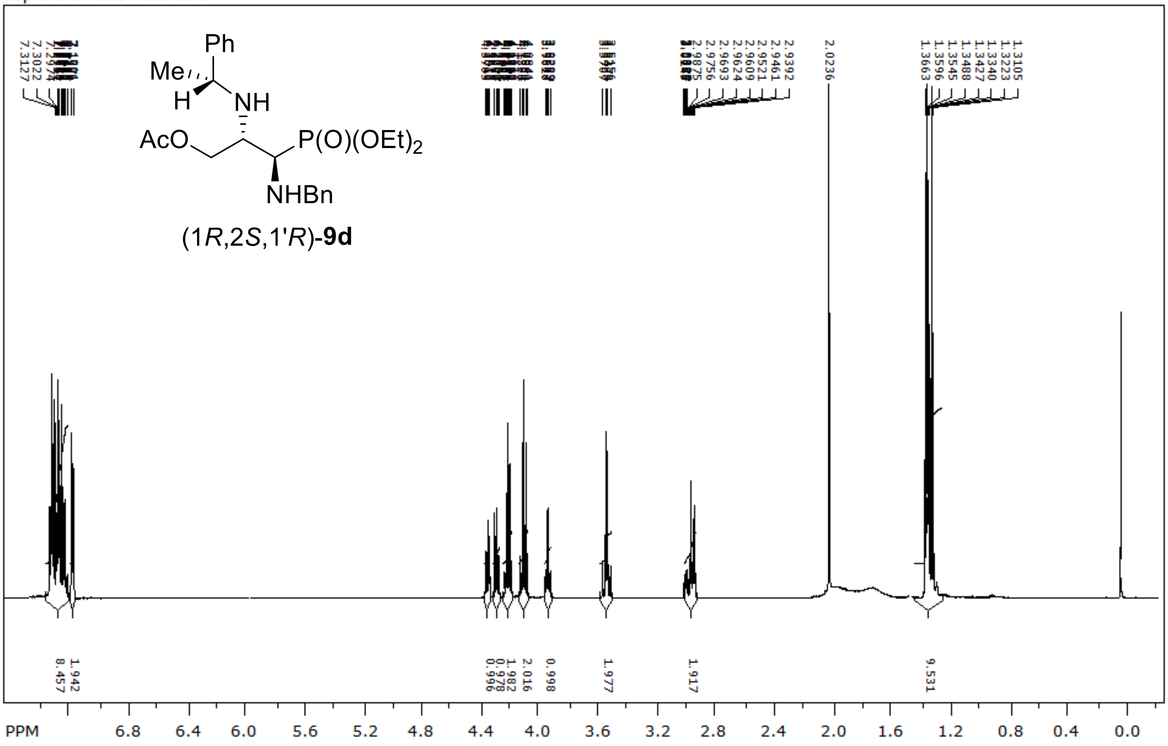

file: ...hodne\(1R,2S,1'R)\abt-1058 31P\fid expt: <zg30>
transmitter freq.: 600.263707 MHz
time domain size: 65536 points
width: 12335.53 Hz = 20.5502 ppm = 0.188225 Hz/pt
number of scans: 16

freq. of 0 ppm: 600.260000 MHz
processed size: 32768 complex points
LB: 0.000 GF: 0.0000
Hz/cm: 189.808 ppm/cm: 0.31621

SpinWorks 3: no title

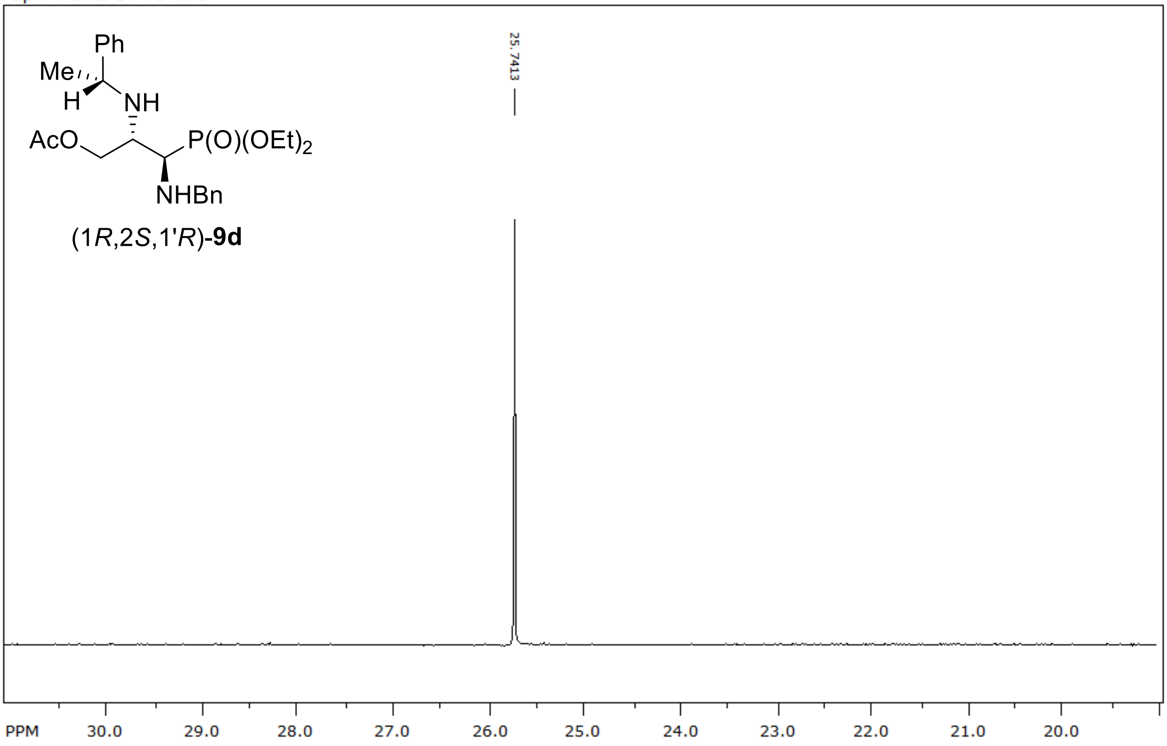

file: ...chodne\(1R,2S,1'R)\abt-1058 1H\fid expt: <zgpg30>
transmitter freq.: 242.977552 MHz
time domain size: 65536 points
width: 96153.85 Hz = 395.7314 ppm = 1.467191 Hz/pt
number of scans: 128

freq. of 0 ppm: 242.989702 MHz
processed size: 32768 complex points
LB: 0.000 GF: 0.0000
Hz/cm: 117.816 ppm/cm: 0.48488

SpinWorks 3:

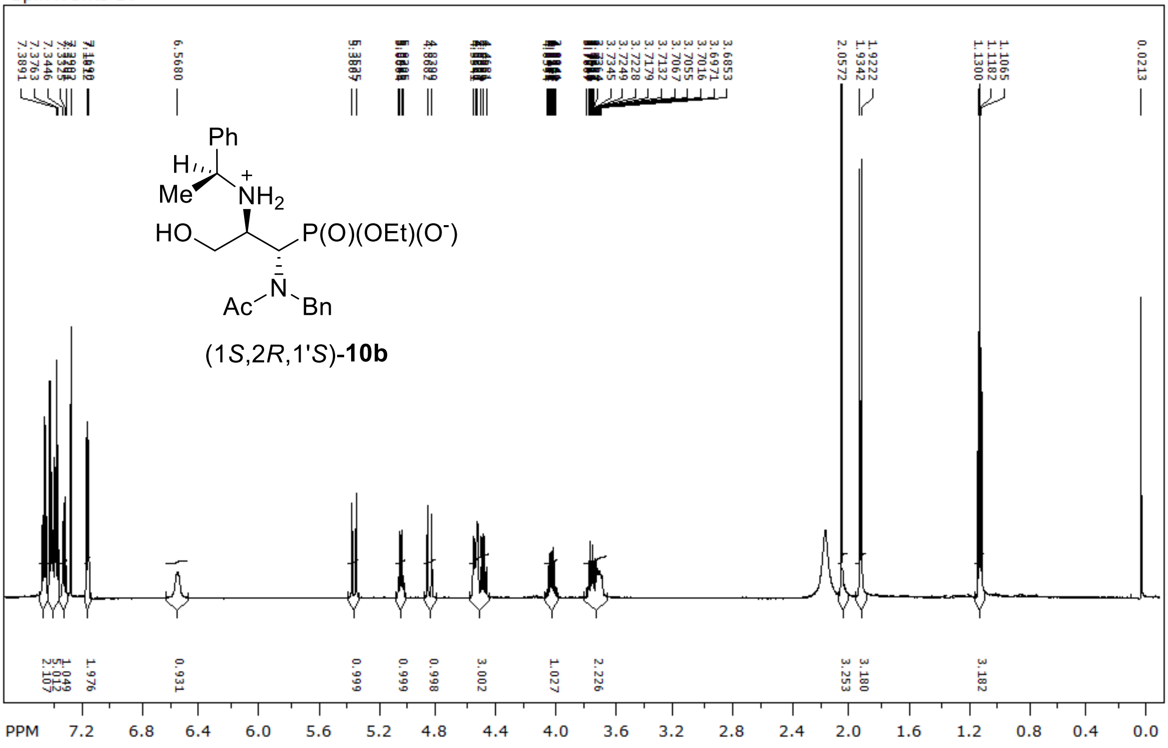

file: ...okwasy\(1S,2R,1'S)\abt-1034 1H\fid expt: <zg30>
transmitter freq.: 600.263707 MHz
time domain size: 65536 points
width: 12335.53 Hz = 20.5502 ppm = 0.188225 Hz/pt
number of scans: 16

freq. of 0 ppm: 600.260000 MHz
processed size: 32768 complex points
LB: 0.000 GF: 0.0000
Hz/cm: 188.614 ppm/cm: 0.31422

SpinWorks 3:

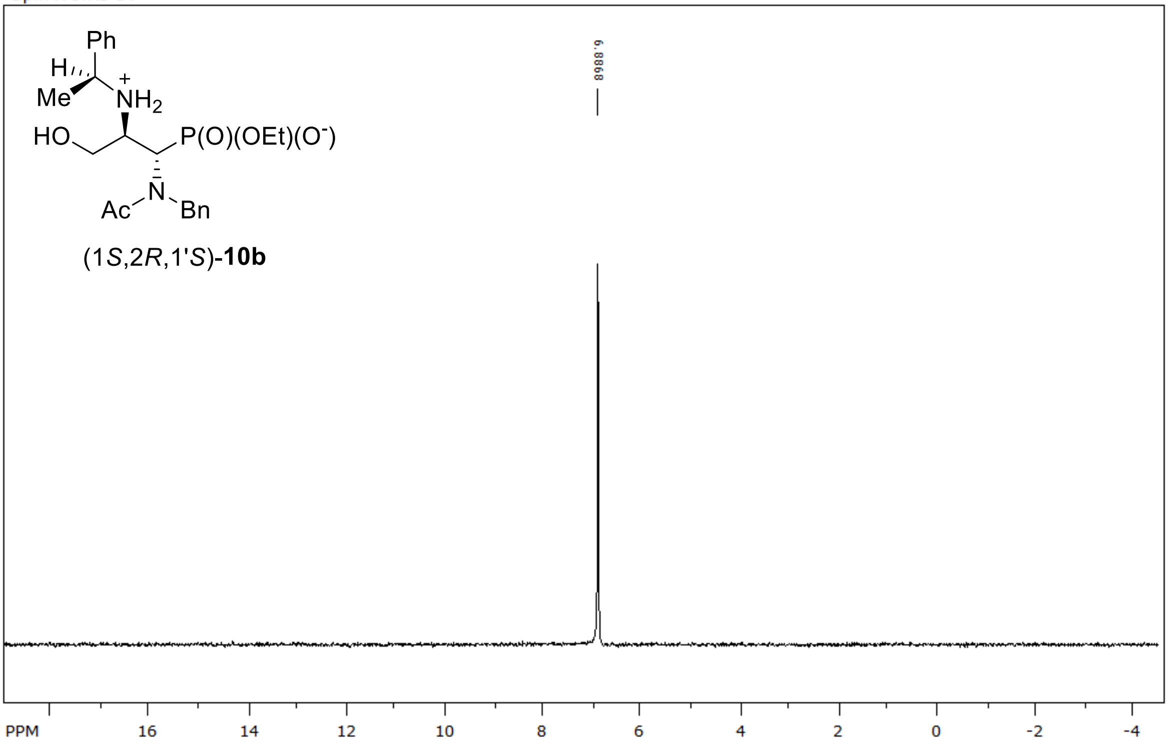

file: ...kwasy\(1S,2R,1'S)\abt-1034 31P\fid expt: <zgpg30>
transmitter freq.: 242.977552 MHz
time domain size: 65536 points
width: 96153.85 Hz = 395.7314 ppm = 1.467191 Hz/pt
number of scans: 128

freq. of 0 ppm: 242.989702 MHz
processed size: 32768 complex points
LB: 0.000 GF: 0.0000
Hz/cm: 229.131 ppm/cm: 0.94301

SpinWorks 3:

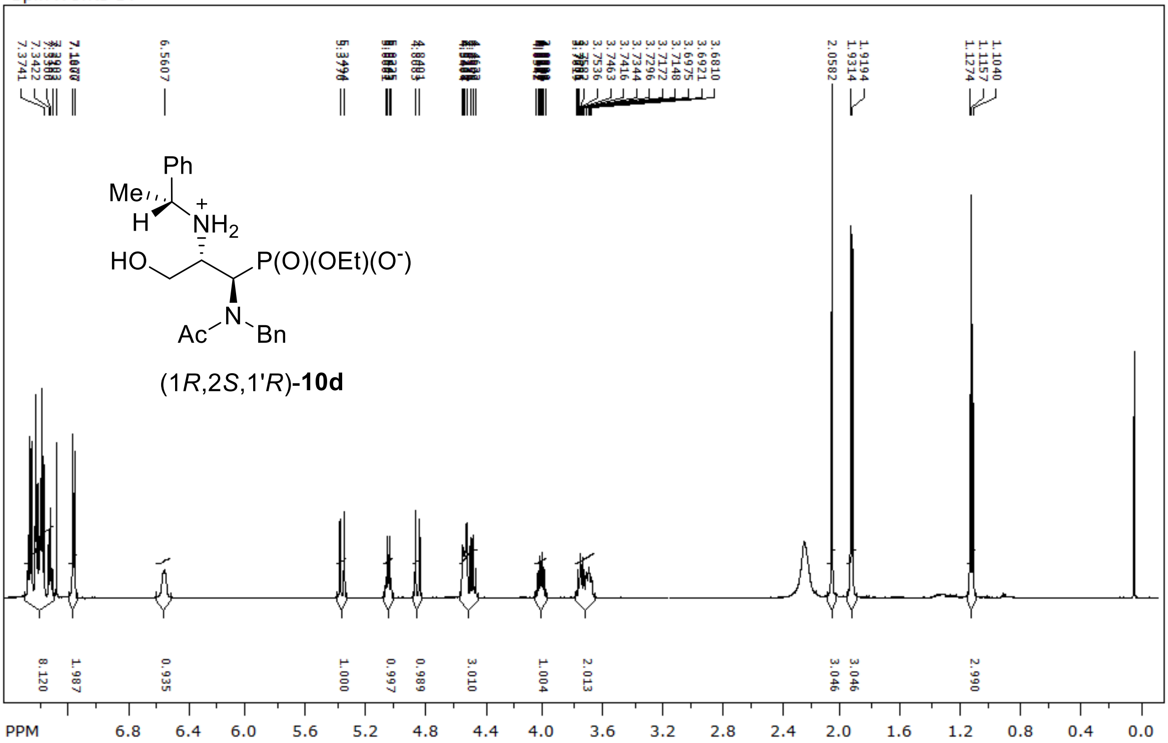

file: ...okwasy\(1R,2S,1'R)\abt-1030 1H\fid expt: <zg30>
transmitter freq.: 600.263707 MHz
time domain size: 65536 points
width: 12335.53 Hz = 20.5502 ppm = 0.188225 Hz/pt
number of scans: 16

freq. of 0 ppm: 600.260000 MHz
processed size: 32768 complex points
LB: 0.000 GF: 0.0000
Hz/cm: 187.420 ppm/cm: 0.31223

SpinWorks 3:

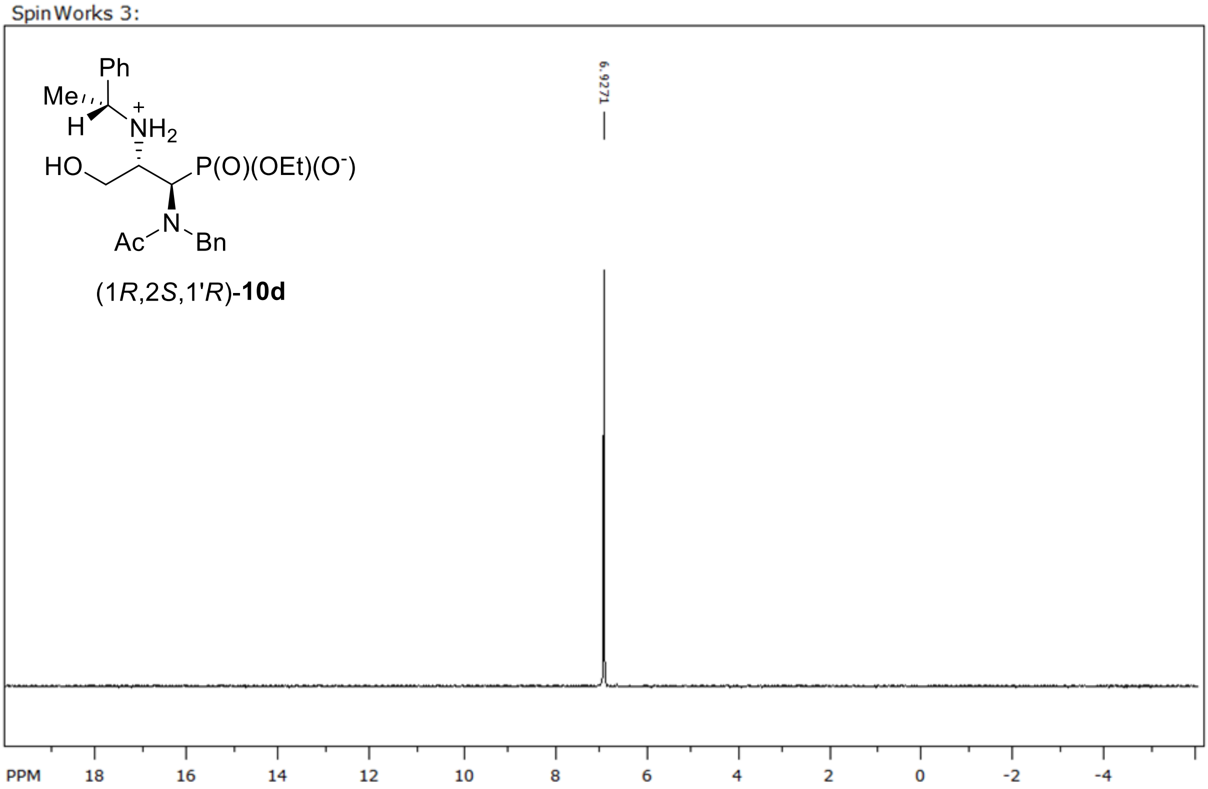

file: ...kwasy\(1R,2S,1'R)\abt-1030 31P\fid expt: <zgpg30>
transmitter freq.: 242.977552 MHz
time domain size: 65536 points
width: 96153.85 Hz = 395.7314 ppm = 1.467191 Hz/pt
number of scans: 128

freq. of 0 ppm: 242.989702 MHz
processed size: 32768 complex points
LB: 0.000 GF: 0.0000
Hz/cm: 254.342 ppm/cm: 1.04677

SpinWorks 3: no title

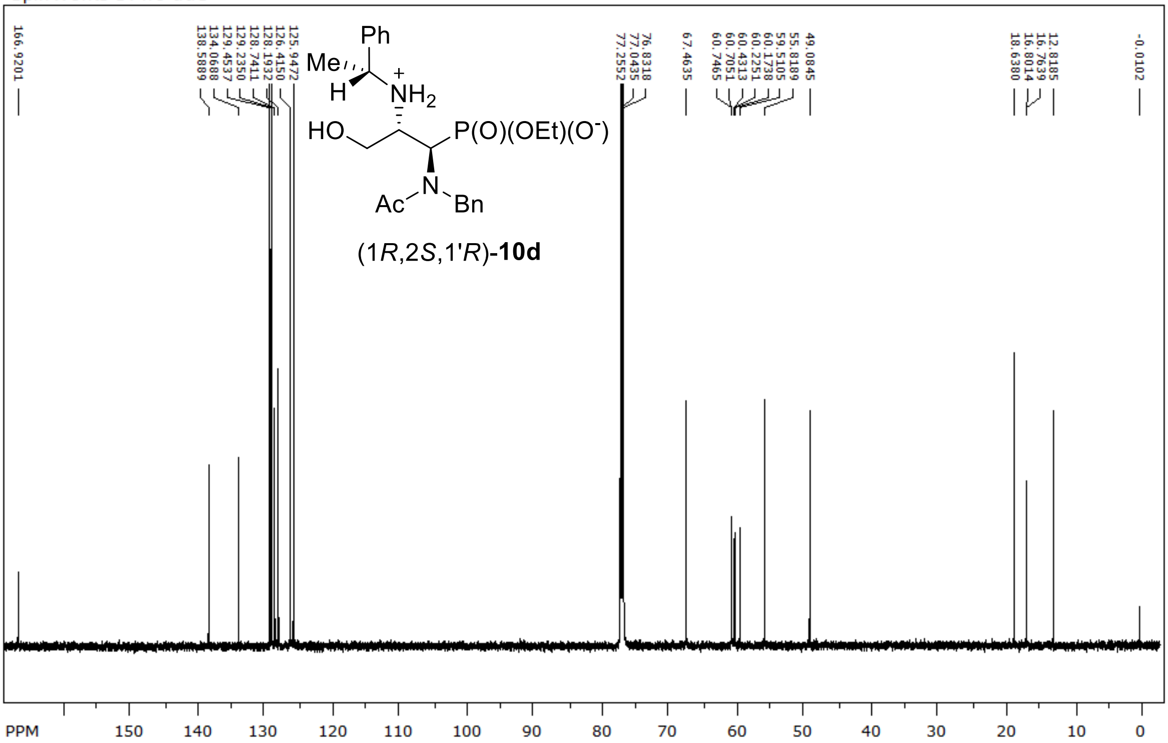

file: ...kwasy\(1R,2S,1'R)\abt-1030 13C\fid expt: <zgpg30>
transmitter freq.: 150.950591 MHz
time domain size: 65536 points
width: 36057.69 Hz = 238.8708 ppm = 0.550197 Hz/pt
number of scans: 4096

freq. of 0 ppm: 150.935497 MHz
processed size: 32768 complex points
LB: 0.000 GF: 0.0000
Hz/cm: 1039.857 ppm/cm: 6.88873

SpinWorks 3:

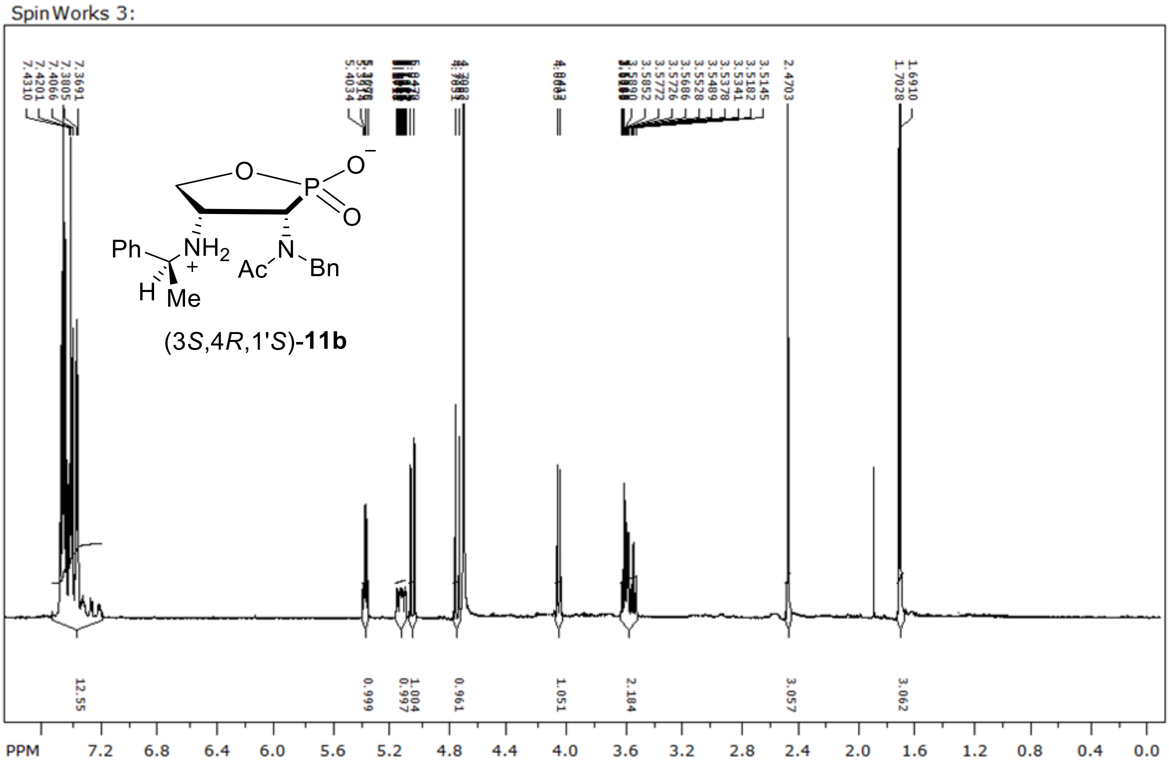

file: ...lany\z (1S,2R,1'S)\abt-1033 1H\fid expt: <zg30>
transmitter freq.: 600.263707 MHz
time domain size: 65536 points
width: 12335.53 Hz = 20.5502 ppm = 0.188225 Hz/pt
number of scans: 16

freq. of 0 ppm: 600.260000 MHz
processed size: 32768 complex points
LB: 0.000 GF: 0.0000
Hz/cm: 191.798 ppm/cm: 0.31952

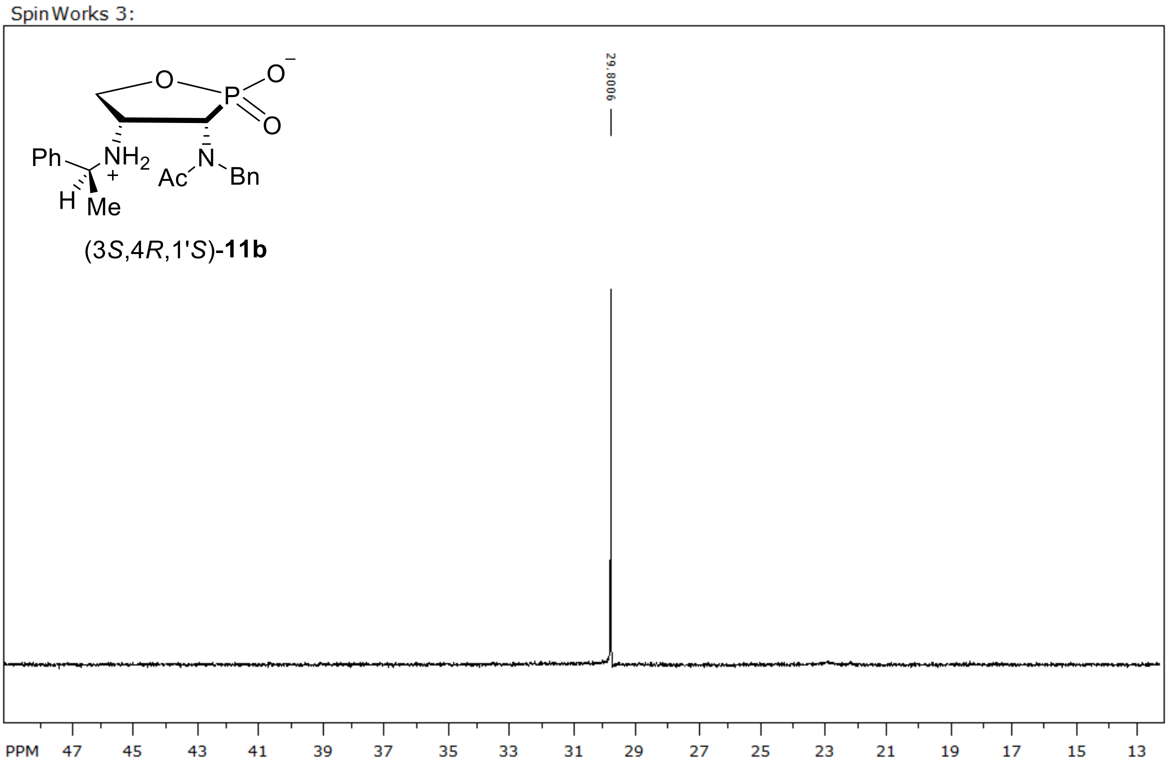

file: ...any\z (1S,2R,1'S)\abt-1033 31P\fid expt: <zgpg30>
transmitter freq.: 242.977552 MHz
time domain size: 65536 points
width: 96153.85 Hz = 395.7314 ppm = 1.467191 Hz/pt
number of scans: 128

freq. of 0 ppm: 242.989702 MHz
processed size: 32768 complex points
LB: 0.000 GF: 0.0000
Hz/cm: 359.801 ppm/cm: 1.48080

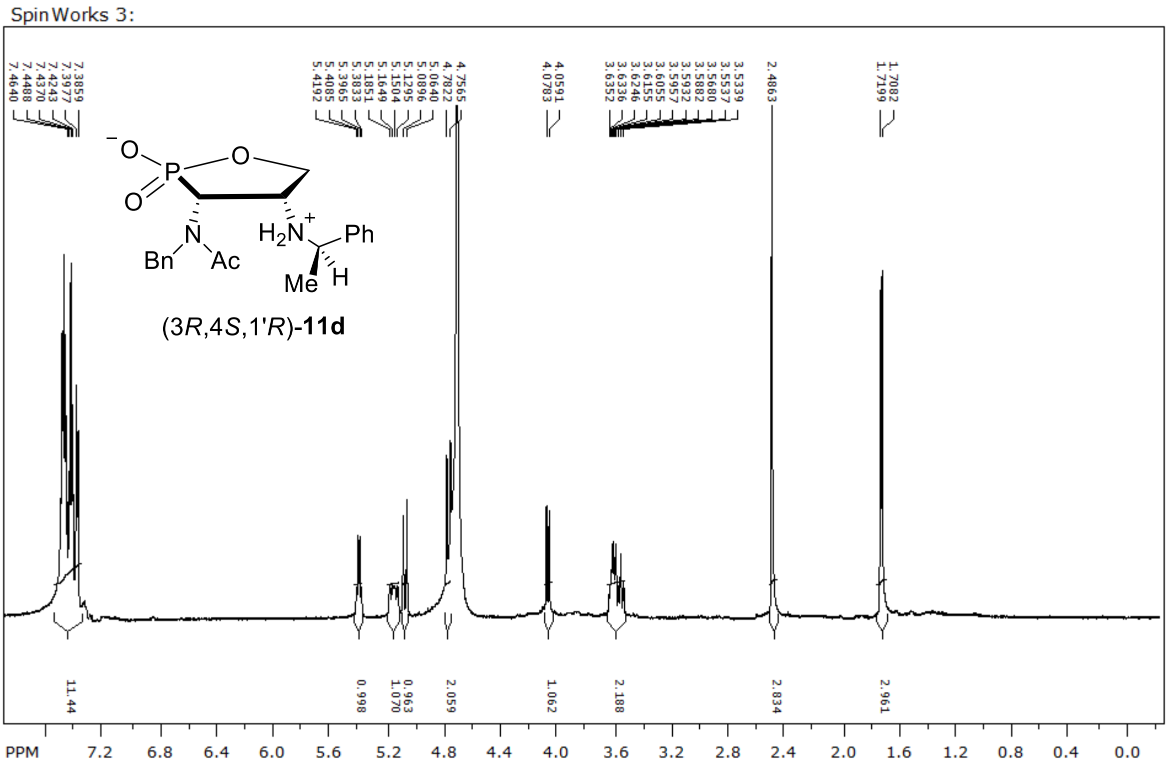

file: ...R,2S,1'R)\abt-1029\abt-1029 1H\fid expt: <zg30>
transmitter freq.: 600.263707 MHz
time domain size: 65536 points
width: 12335.53 Hz = 20.5502 ppm = 0.188225 Hz/pt
number of scans: 16

freq. of 0 ppm: 600.260000 MHz
processed size: 32768 complex points
LB: 0.000 GF: 0.0000
Hz/cm: 196.175 ppm/cm: 0.32681

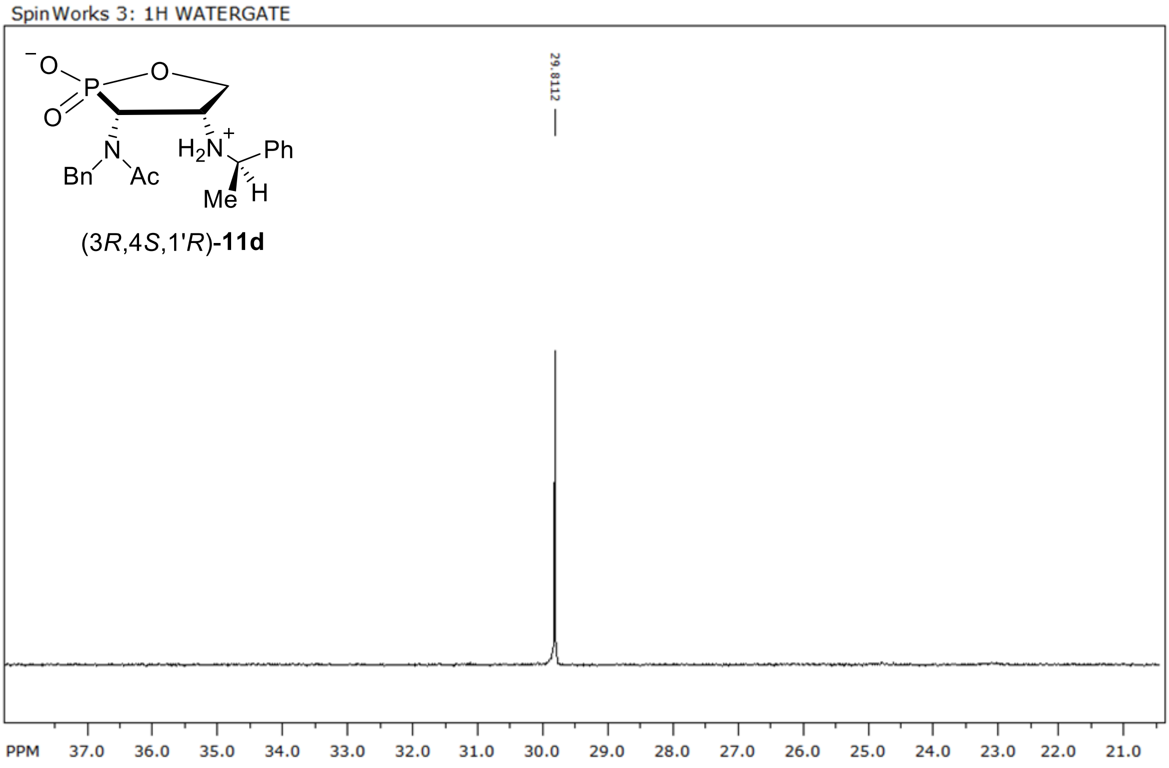

file: ...,2S,1'R)\abt-1029\abt-1029 31P\fid expt: <zgpg30>
transmitter freq.: 242.977552 MHz
time domain size: 65536 points
width: 96153.85 Hz = 395.7314 ppm = 1.467191 Hz/pt
number of scans: 64

freq. of 0 ppm: 242.989702 MHz
processed size: 32768 complex points
LB: 0.000 GF: 0.0000
Hz/cm: 174.298 ppm/cm: 0.71734

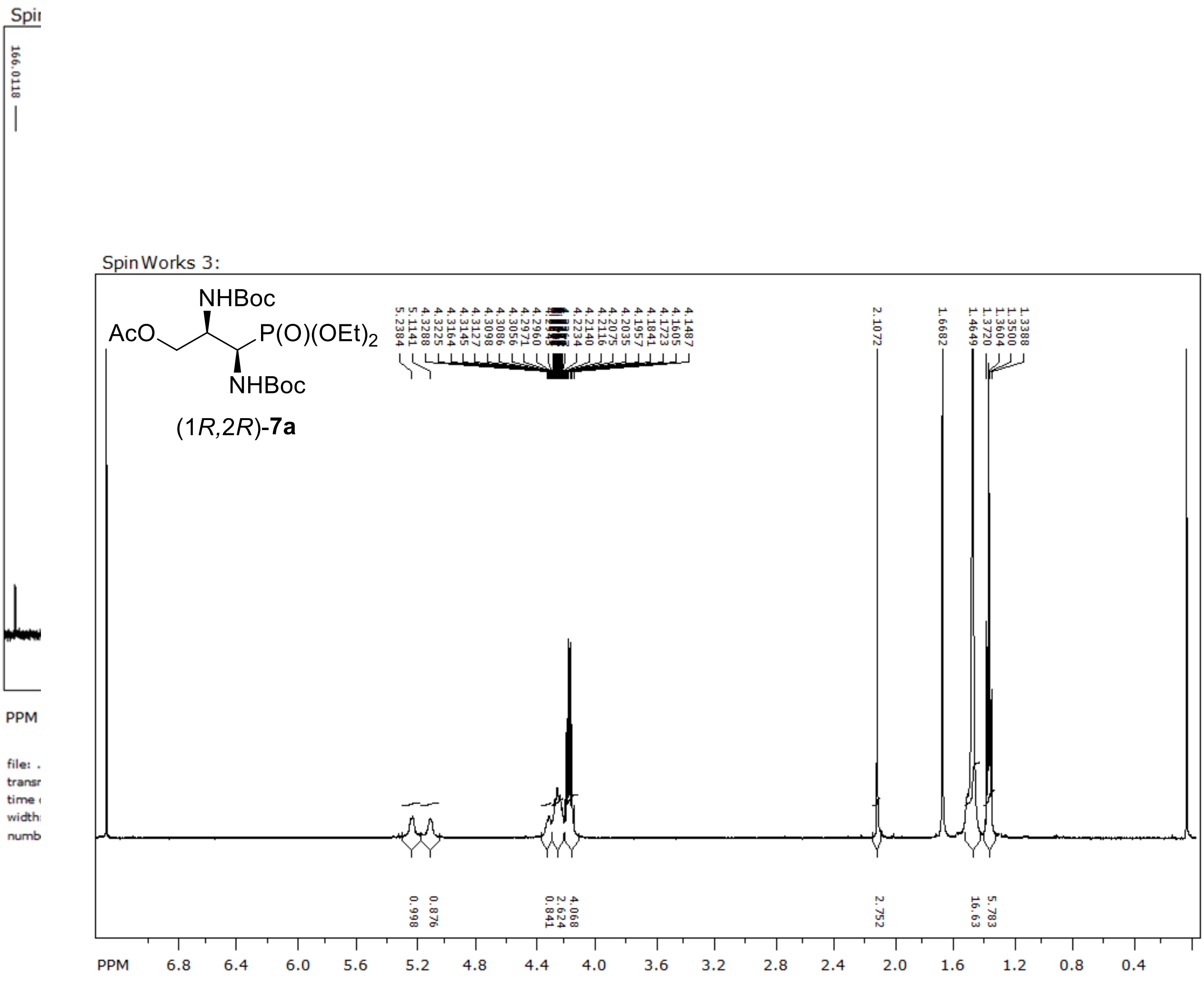

file: ...odorolizie\(1R,2R)\abt-1295 1H\fid expt: <zg30>
transmitter freq.: 600.263707 MHz
time domain size: 65536 points
width: 12335.53 Hz = 20.5502 ppm = 0.188225 Hz/pt
number of scans: 16

freq. of 0 ppm: 600.260000 MHz
processed size: 32768 complex points
LB: 0.000 GF: 0.0000
Hz/cm: 177.870 ppm/cm: 0.29632

SpinWorks 3:

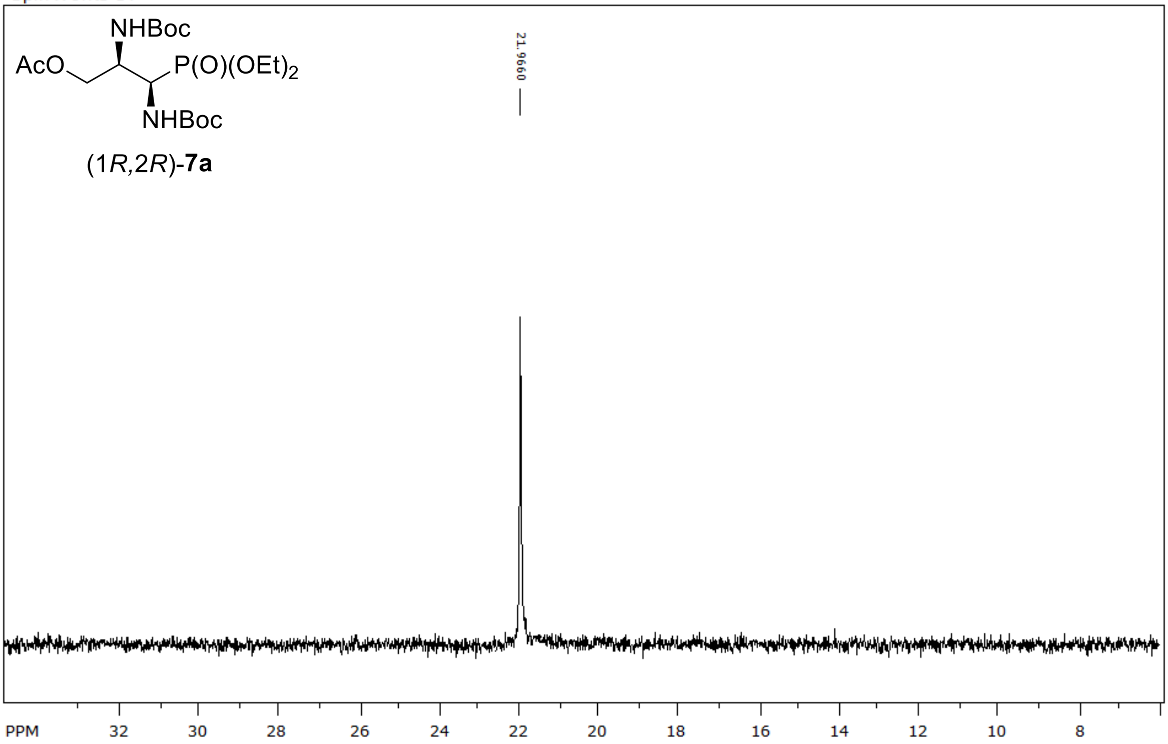

file: ...dorolizie\(1R,2R)\abt-1295 31P\fid expt: <zgpg30>
transmitter freq.: 242.977552 MHz
time domain size: 65536 points
width: 96153.85 Hz = 395.7314 ppm = 1.467191 Hz/pt
number of scans: 128

freq. of 0 ppm: 242.989702 MHz
processed size: 32768 complex points
LB: 0.000 GF: 0.0000
Hz/cm: 282.258 ppm/cm: 1.16166

SpinWorks 3: no title

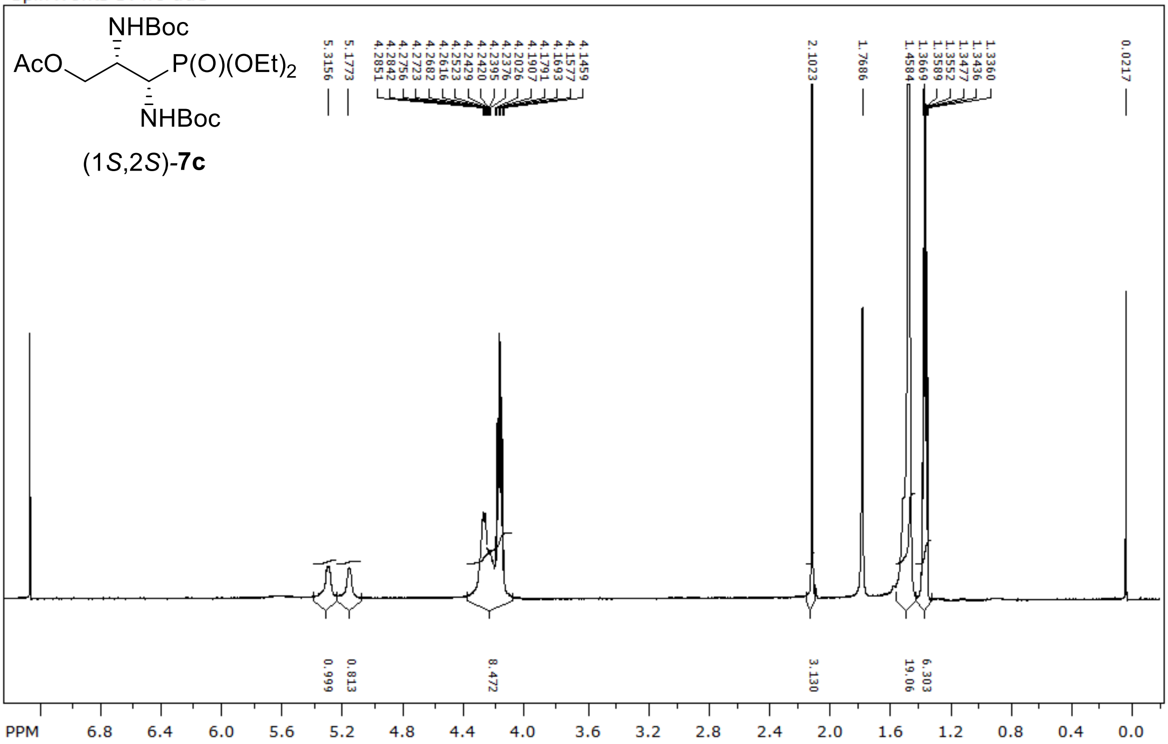

file: ...odorolizie\(1S,2S)\abt-1285 1H\fid expt: <zg30>
transmitter freq.: 600.263707 MHz
time domain size: 65536 points

freq. of 0 ppm: 600.260000 MHz
processed size: 32768 complex points
LB: 0.000 GF: 0.0000

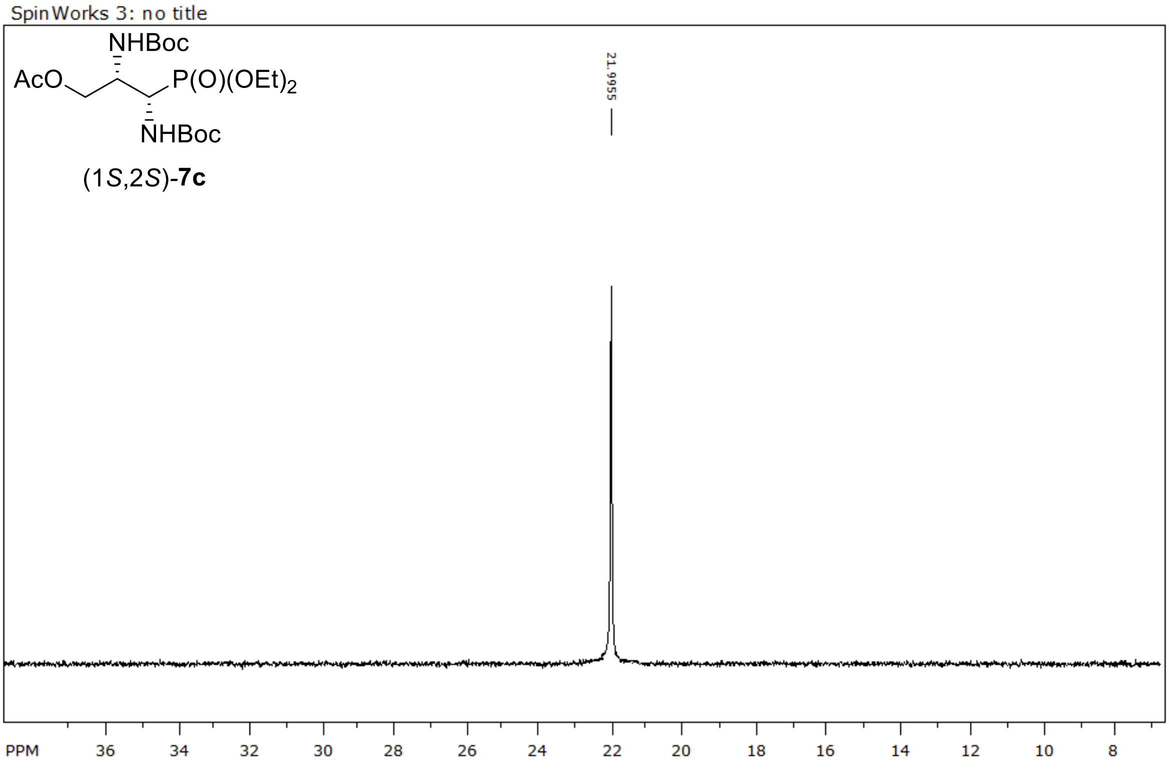

file: ...dorolizie\(1S,2S)\abt-1285 31P\fid expt: <zgpg30>
transmitter freq.: 242.977552 MHz
time domain size: 65536 points
width: 96153.85 Hz = 395.7314 ppm = 1.467191 Hz/pt
number of scans: 128

freq. of 0 ppm: 242.989702 MHz
processed size: 32768 complex points
LB: 0.000 GF: 0.0000
Hz/cm: 313.275 ppm/cm: 1.28932

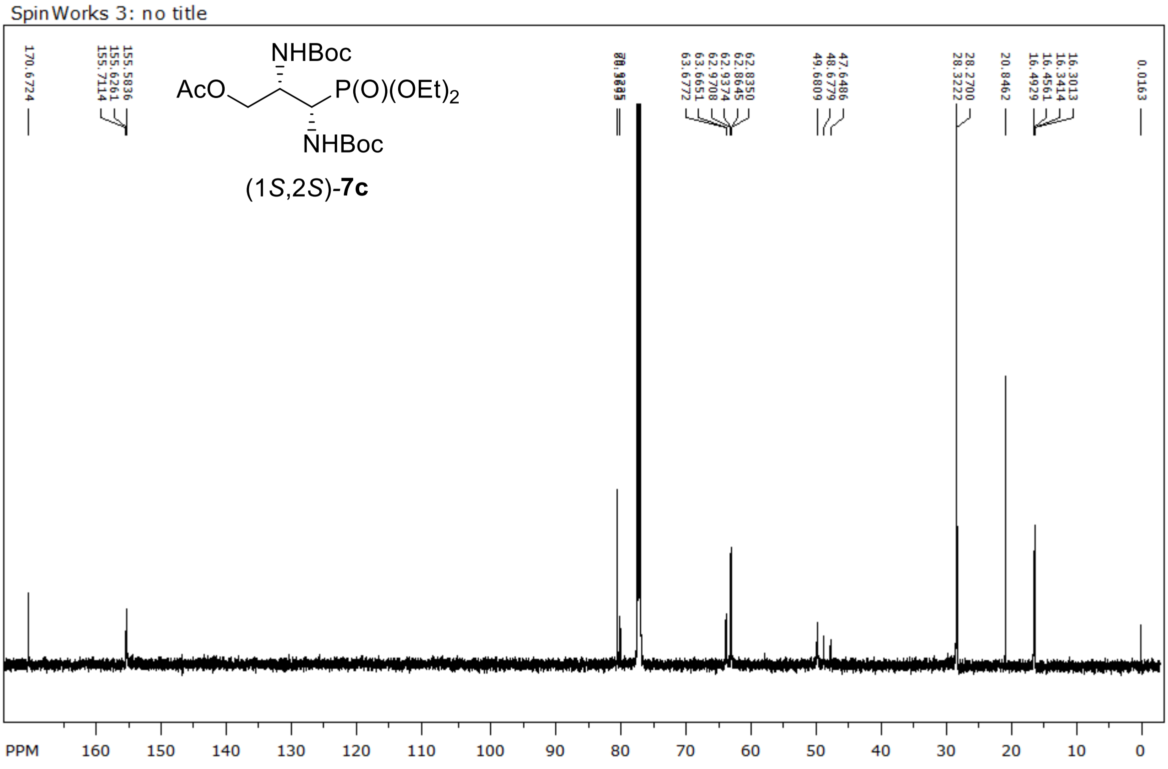

file: ...dorolizie\(1S,2S)\abt-1285 13C\fid expt: <zgpg30>
transmitter freq.: 150.950591 MHz
time domain size: 65536 points
width: 36057.69 Hz = 238.8708 ppm = 0.550197 Hz/pt
number of scans: 5120

freq. of 0 ppm: 150.935497 MHz
processed size: 32768 complex points
LB: 0.000 GF: 0.0000
Hz/cm: 1071.262 ppm/cm: 7.09678

SpinWorks 3: no title

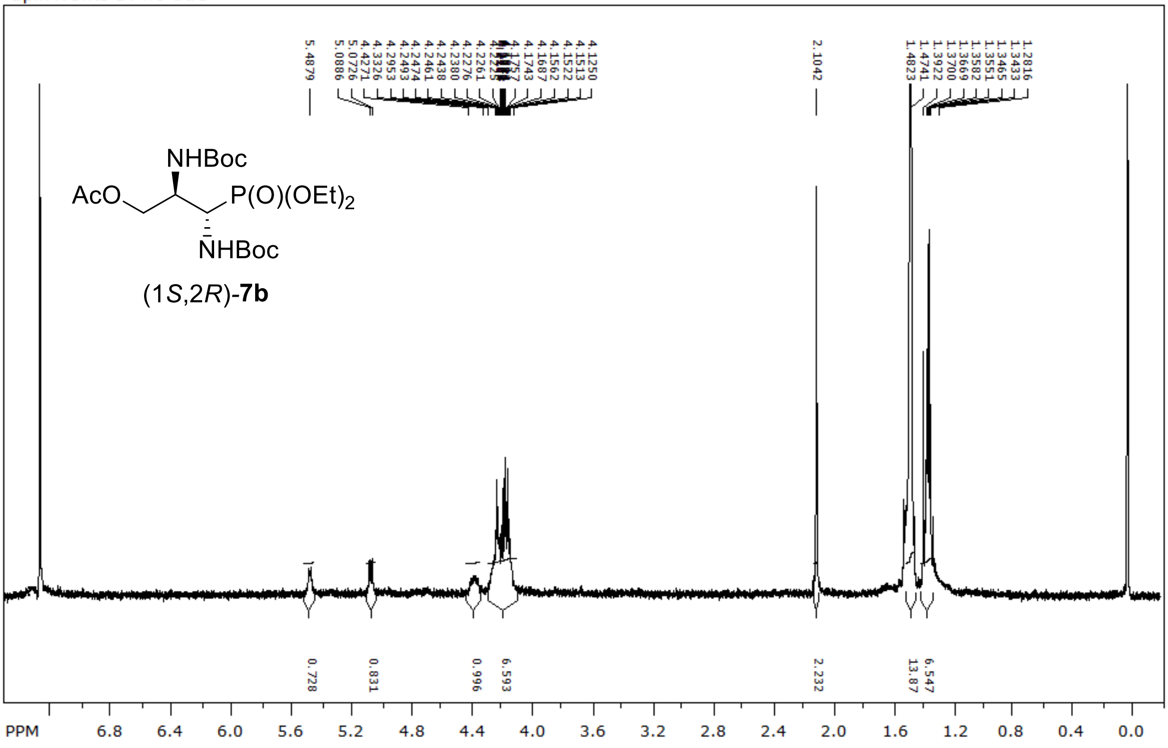

file: ...odorolizie\(1S,2R)\abt-1328 1H\fid expt: <zg30>
transmitter freq.: 600.263707 MHz
time domain size: 65536 points
width: 12335.53 Hz = 20.5502 ppm = 0.188225 Hz/pt
number of scans: 16

freq. of 0 ppm: 600.260000 MHz
processed size: 32768 complex points
LB: 0.000 GF: 0.0000
Hz/cm: 185.431 ppm/cm: 0.30892

SpinWorks 3: no title

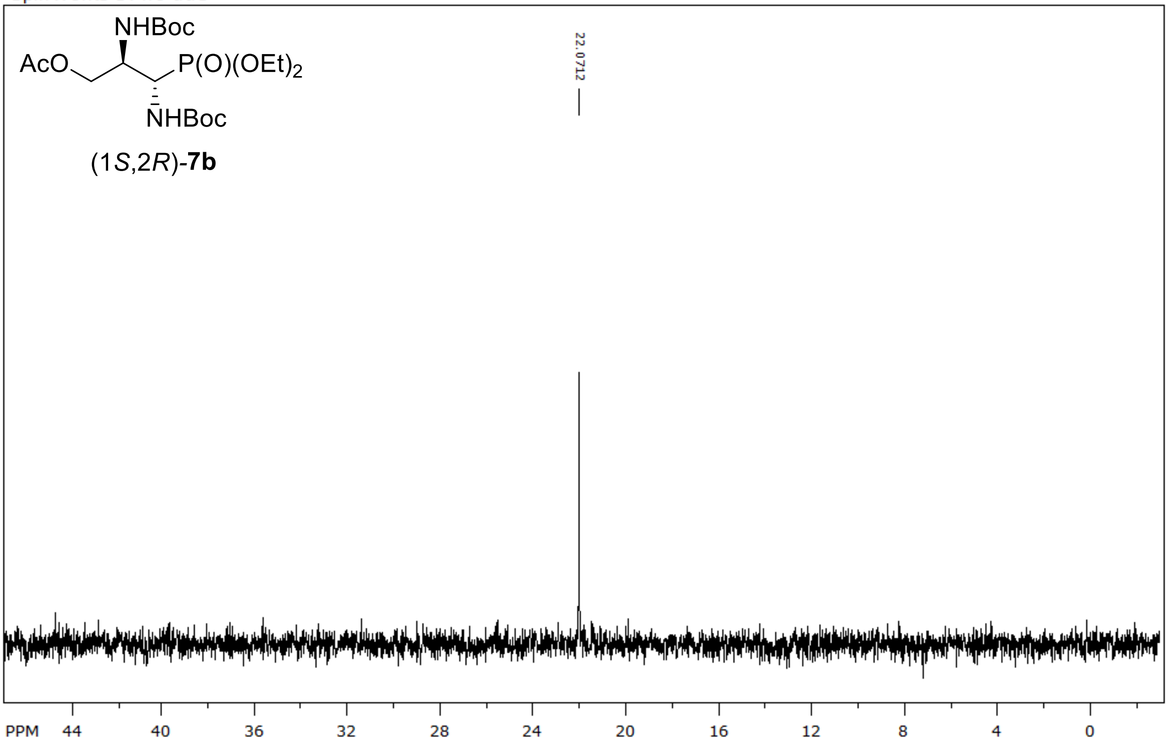

file: ...dorolizie\(1S,2R)\abt-1328 31P\fid expt: <zgpg30>
transmitter freq.: 242.977552 MHz
time domain size: 65536 points
width: 96153.85 Hz = 395.7314 ppm = 1.467191 Hz/pt
number of scans: 128

freq. of 0 ppm: 242.989702 MHz
processed size: 32768 complex points
LB: 0.000 GF: 0.0000
Hz/cm: 486.973 ppm/cm: 2.00419

SpinWorks 3: 13C.stan

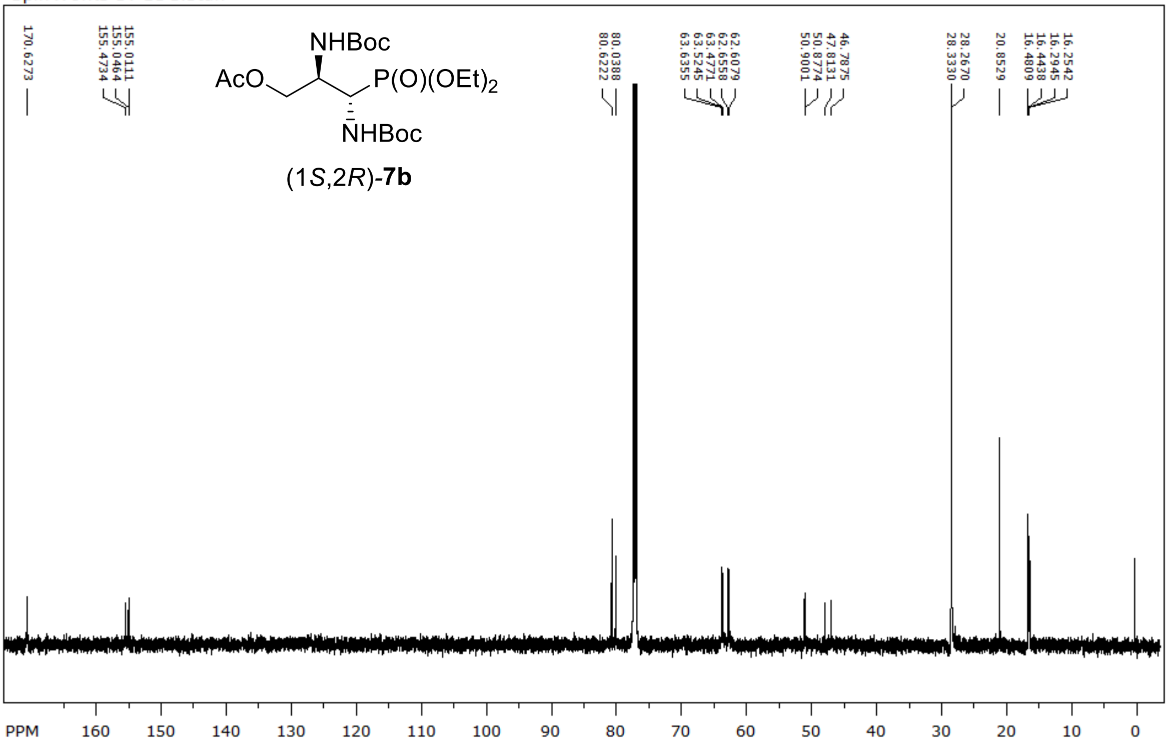

file: ...dorolizie\(1S,2R)\abt-1311 13C\fid expt: <zgpg30>
transmitter freq.: 150.950591 MHz
time domain size: 65536 points
width: 36057.69 Hz = 238.8708 ppm = 0.550197 Hz/pt
number of scans: 5120

freq. of 0 ppm: 150.935497 MHz
processed size: 32768 complex points
LB: 0.000 GF: 0.0000
Hz/cm: 1075.915 ppm/cm: 7.12760

SpinWorks 3: no title

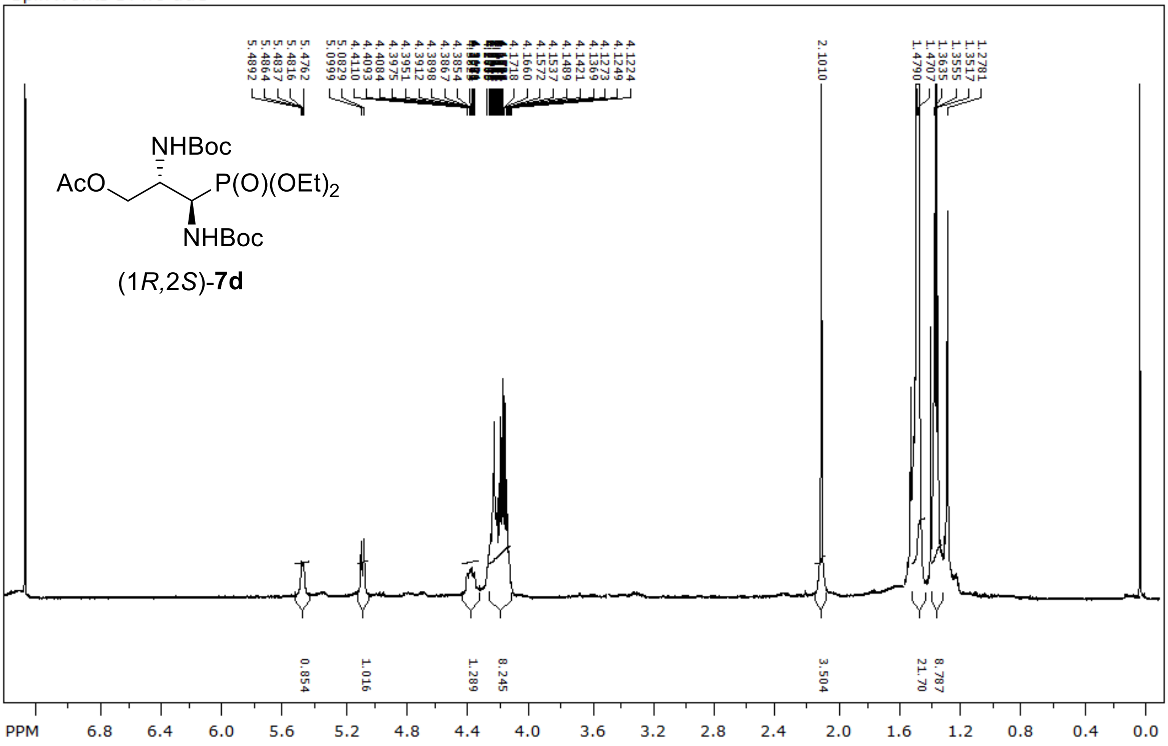

file: ...odorolizie\(1R,2S)\abt-1324 1H\fid expt: <zg30>
transmitter freq.: 600.263707 MHz
time domain size: 65536 points
width: 12335.53 Hz = 20.5502 ppm = 0.188225 Hz/pt
number of scans: 16

freq. of 0 ppm: 600.260000 MHz
processed size: 32768 complex points
LB: 0.000 GF: 0.0000
Hz/cm: 181.054 ppm/cm: 0.30162
